# Single Molecule Eu^2+/3+^ Complex Platform for Optical and Magnetic Resonance Imaging In Vivo

**DOI:** 10.1021/jacs.6c08257

**Published:** 2026-06-06

**Authors:** Carter B. Rodgers, Morgan P. Deal, Leah C. Garman, Ilia A. Guzei, Matthew J. Allen, Eszter Boros

**Affiliations:** † Department of Chemistry, 5228 University of Wisconsin, 1101 University Avenue, Madison, Wisconsin 53706, United States; ‡ Department of Chemistry, 2954 Wayne State University, 5101 Cass Avenue, Detroit, Michigan 48202, United States

## Abstract

Lanthanide coordination complexes are harnessed for biological imaging due to their oxidative stability in aqueous media, favorable relaxometric behavior, and accessible luminescence emissions within biomedically relevant, optical-imaging wavelength ranges. In contrast with multimodal imaging strategies that rely on exchanging lanthanide ions to access distinct modalities, we exploit the unique redox chemistry of the Eu^2+/3+^ pair to demonstrate the feasibility of multimodal imaging within a single chelate scaffold. We constructed a series of polypyridine-containing macrocyclic ligands that readily coordinate both Eu^2+^ and Eu^3+^ ions. Characterization of the corresponding chelates by X-ray crystallography, cyclic voltammetry, electron paramagnetic resonance spectroscopy, and photophysical measurements were conducted. The Eu^2+^-containing complexes of acetamide-functionalized, polypyridine-containing 18-membered macrocycles exhibit relaxometric properties comparable to clinical Gd^3+^ contrast agents for magnetic resonance imaging. Upon oxidation to Eu^3+^, the complexes display characteristic luminescence with quantum yields ranging from 1.3 to 13.9%. In situ sensitization with a Cherenkov-emitting radionuclide efficiently produces Eu^3+^ emission at concentrations comparable to those employed for Eu^2+^-enhanced magnetic resonance imaging. The complex that provided the greatest signal-to-noise ratio in magnetic resonance in vitro imaging studies, and when oxidized, produced a detectable, optical imaging signal with as little as 5 nmol of complex. A subsequent study in a murine xenograft tumor model demonstrated the feasibility of conducting sequential magnetic resonance and optical imaging experiments following single dose administration of the redox–switchable complex.

## Graphical abstract

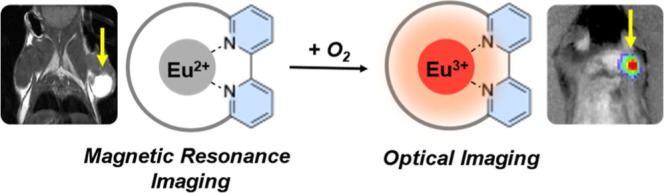

## Introduction

Trivalent lanthanides have attractive properties for biomedical applications, including radiotherapy (^177^Lu^3+^ for beta therapy),^1^ magnetic resonance imaging (MRI, Gd^3+^),^2^ and optical imaging (Eu^3+^, Tb^3+^, Yb^3+^, validated in preclinical models).^3^ With respect to optical imaging, lanthanides display large Stokes shifts, long luminescence lifetimes, and characteristic narrow emission bands (<150 cm^–1^) that provide advantages over organic fluorophores.^4–6^ However, lanthanide-based optical imaging remains challenging to implement on a clinical scale. Because of the Laporte and spin-forbidden nature of f–f transitions, lanthanides exhibit low molar absorptivity values. The installation of organic chromophores (antenna) in the immediate vicinity of Ln^3+^ ions that undergo Dexter-mediated energy transfer from the triplet state of the chromophore to the excited state of the lanthanide ions is a common strategy to enhance the sensitization efficiency of relevant excited states.^7^ For lanthanide ions that emit in the near-IR-I (NIR-I) window, effective triplet sensitization energies are generally >22,000 cm^–1^. To achieve efficient short-wavelength sensitization without quenching by surrounding tissues and proteins, our group has previously established the in situ excitation of lanthanide complexes using Cherenkov radiation (CR).^8–13^ CR is produced by radioisotopes that emit charged particles that move faster than the speed of light in a dielectric medium, such as water, emitting light with increased intensity in the UV–blue spectral region. For instance, the clinically employed isotope ^68^Ga (*t*_1/2_ = 68 min, β_avg_ = 0.89 MeV, 89% positron decay) is well suited to produce CR for the sensitization of luminescent Tb^3+^ and Eu^3+^ complexes.

Although lanthanides have been extensively studied in their trivalent state for biological applications, complexes of all of the lanthanides, expect *Pm*, have been reported as the Ln^2+^ ion, including Eu^2+^.^14–17^ The electronic structure of divalent Eu results in fundamentally different photophysical and magnetic properties than Eu^3+^.^18–23^ Unlike the narrow f–f emissions of Eu^3+^, Eu^2+^ is associated with 4f^7^ → 4f^6^5d^1^ transitions that are accessible with molar absorptivities greater than 1000 M^–1^ cm^–1^, negating the need for sensitization with an antenna. As a consequence of the valence d-orbital involvement, emissions are dependent on ligand field and solvent environment, with emissions appearing as characteristically broad bands >5000 cm^–1^.^24,25^

The optical and relaxometric properties of the Eu^2+/3+^ pair have the potential to enable MRI and luminescence imaging applications within the same chelate. However, to date, lanthanide-based multimodality combining optical and magnetic resonance imaging has generally been achieved using different lanthanide complexes with the same ligand scaffold. For instance, the Tóth group used 1,4,7-tris­(carboxymethyl)-1,4,7,10-tetraazacyclododecane (DO3A)-pyridine compounds to combine the relaxometric properties of gadolinium with the NIR emission and chemical exchange saturation transfer (CEST) MRI activity of Yb^3+^ (Figure 1).^26^ Similarly, Senèque and co-workers used DO3A-acetophenone ligands to combine Gd^3+^ MRI with Eu^3+^-based, two-photon microscopy imaging (Figure 1).^27^ Only a few examples have probed imaging applications that involve both oxidation states of europium, but with in vivo imaging restricted to the divalent state.^28–33^

**Figure 1:**
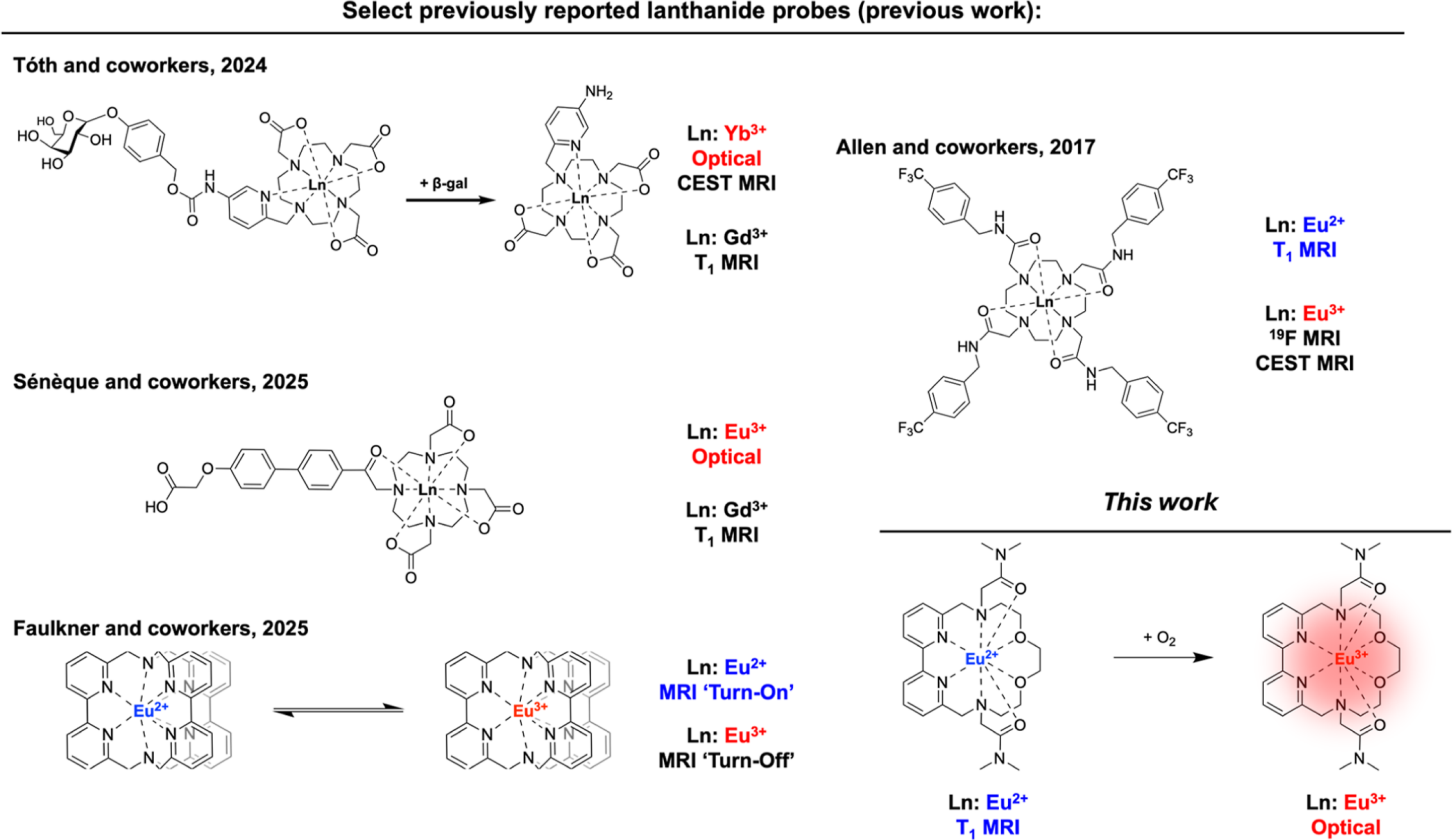
Select, previously reported lanthanide probes for optical imaging applications. Conventional bimodal imaging approaches often require the use of two different lanthanides, whereas few reports leverage the Eu­(^2+/3+^) couple for more than a turn-on/turn-off probe. This work reports a single ligand scaffold to allow magnetic resonance imaging with Eu^2+^ and optical imaging with Eu^3+^.

Here, we leverage the Eu^2+/3+^ pair to toggle between magnetic and optical imaging modes within the same chelate. Taking into consideration the distinct coordinative preferences of the Eu^2+/3+^ pair,^34^ we demonstrate that an aromatic, 18-membered macrocycle ligand scaffold with flexible acetamide donor arms forms inert complexes with europium in both the +2 and +3 oxidation states. The Eu^2+^-containing complexes are explored for their relaxometric properties and their ability to recover characteristic Eu^3+^ luminescence when oxidized.

## Results and Discussion

### Chemical Synthesis

We first examined precedent reporting ligand scaffolds that flexibly incorporate the Eu^2+/3+^ pair. Indeed, cryptands have been extensively explored to stabilize Eu^2+^,^35–38^ but they lack affinity for the Eu^3+^ ion.^39,40^ Tetra-azamacrocyclic compounds provide a suitable alternative in this regard, with the Eu^2+^ complexes exhibiting lower thermodynamic stability; steric crowding has been used to enhance kinetic inertness, preventing dechelation.^41^

To balance the disparate coordinative and electronic preferences of the two ions, we proposed a flexible, diaza-18-crown-6 functionalized ligand architecture, where the sensitizing polypyridyl ligand is integrated into the backbone of the crown (Figure 2).^12,42^ We posited that incorporation of acetamide donor arms would provide the flexibility necessary to accommodate both Eu^2+^ and Eu^3+^ ions.

**Figure 2:**
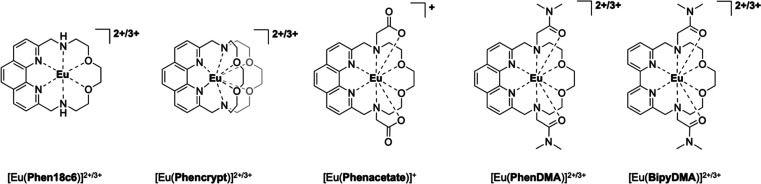
Chemical structures and nomenclature of the synthesized complexes. The structures drawn only depict the metal and the title ligand, omitting inner-sphere ternary ligands and counterions.

The 18-membered macrocycle **Phen18c6** was synthesized following precedent.^43,44^ In brief, oxidation of commercially available neocuproine using selenium dioxide affords dialdehyde **1** that undergoes a reductive amination with the commercially available diamino-dioxa-octane to produce **Phen18c6** (Scheme S1). This compound was reported to be air-sensitive;^44^ and our observations affirmed the reactive nature of this intermediate by transformation of freshly isolated **Phen18c6** from a yellow powder to a dark oil upon prolonged exposure to ambient conditions. The secondary amines were further alkylated with bromo-*N*,*N*-dimethylacetamide to form **PhenDMA**. Alternatively, alkylation with ethyl bromoacetate under analogous conditions, followed by deprotection of the ethyl protecting groups, afforded **Phenacetate**. We also explored the synthesis of a phenanthroline-containing cryptand, **Phencrypt**, that was synthesized using a macrocyclization under high dilution conditions (Scheme S1).^45^ Additionally, dialdehyde **2** was subjected to reductive amination to afford **Bipy18c6** followed by alkylation with bromo-*N*,*N*-dimethylacetamide to furnish **BipyDMA**.

Complexation reactions to afford Eu^2+^-containing complexes were conducted with divalent halide salts, EuX_2_ (X = Br, I) in the absence of oxygen. [Eu­(**Phen18c6**)]^2+^ and [Eu­(**Phencrypt**)]^2+^ formed as dark precipitate in acetonitrile and were isolated in moderate yields. The acetamide-bearing ligands were soluble in water, enabling complexation reactions in methanolic and aqueous solutions. [Eu­(**PhenDMA**)]^2+^ and [Eu­(**BipyDMA**)]^2+^ produce dark-red solutions with complexes that precipitated as red–brown solids upon addition of tetrahydrofuran. The corresponding Eu^3+^-containing complexes were formed under similar conditions, with **Phen18c6** and **Phencrypt** requiring reflux in acetonitrile to achieve complexation.^46^

### Structural Characterization

Solid-state single crystal X-ray structural analysis enabled comparison of structural parameters across the ligand series and between different redox states (Figure 3).^47^

**Figure 3:**
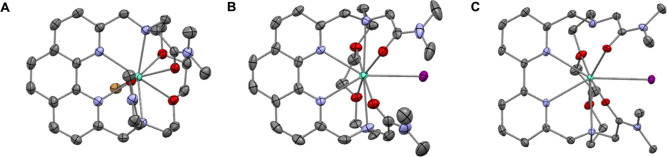
Molecular structures in crystals of (A) [Eu­(**PhenDMA**)­Br]­OTf_2_; (B) [Eu­(**PhenDMA**)­I]­I; (C) [Eu­(**BipyDMA**)­I]­I. Hydrogens, outer-sphere counterions, and solvent molecules are omitted for clarity, thermal ellipsoids are drawn at 50%. Atom color: dark gray = carbon, blue = nitrogen, red = oxygen, purple = iodide, orange = bromide.

The divalent [Eu­(**Phencrypt**)­(H_2_O)_2_]­I_2_ crystallizes as a ten-coordinate complex with a distorted coordination polyhedron most similar to a staggered dodecahedron (*D*_*2*_ symmetry, 6.588% deviation) as determined by SHAPE analysis.^48^ The bond distances are in good agreement with reported divalent europium-containing complexes (Table S5).^35,49,50^ The corresponding Eu^3+^-containing complex, [Eu­(**Phencrypt**)­Br]­Br_2_, exhibited a coordination number of nine with a hula-hoop geometry (C_*s*_ symmetry, 2.892% deviation) along with contraction of the Eu–N and Eu–O bond lengths relative to the divalent counterpart (see Table 1).

**Table 1:** Key Bond Distances (Å) and Angles in [Eu­(**BipyDMA**)­I]­I; [Eu­(**PhenDMA**)­I]­I; and [Eu­(**PhenDMA**)­Br]­OTf_2_

	[Eu(PhenDMA)Br]OTf_2_	[Eu(PhenDMA)I]I	[Eu(BipyDMA)I]I
Eu–N (N/Phen, Bipy)	2.550 (1)	2.678 (2)	2.7180 (9)
Eu–O	2.5252 (13)	2.756 (4)	2.7951 (8)
Eu–amide	2.3829 (18)	2.585 (2)	2.5710 (8)
Eu–X (X: Br, I)	2.9008 (4)	3.2936 (9)	3.3341 (5)
amide–Eu–amide	69.59°	66.875°	68.049°

The crystal structures of [Eu­(**PhenDMA**)­Br]­OTf_2_ and [Eu­(**PhenDMA**)­I]I both reveal nine-coordinate environments with inner-sphere halides. The divalent structure exhibits a more distorted muffin geometry (C_*s*_ symmetry, 8.648% deviation) compared to the trivalent structure with a muffin polyhedron (C_*s*_ symmetry, 3.055% deviation). [Eu­(**PhenDMA**)­Br]­OTf_2_ reveals bond lengths consistent with Eu^3+^.^51^ Comparison of the acetamide structures highlights the structural flexibility of these chelates to accommodate Eu in both the +2 and +3 oxidation states (Figure S94). Comparing the bond lengths (Table 2), an elongation of the Eu–O and Eu–N^phen^ bonds supports the presence of Eu in the divalent oxidation state.

**Table 2:** Photophysical Characterization of the Trivalent Europium Compounds in H_2_O and D_2_O

	τ D_2_O (ms)	τ H_2_O (ms)	*q* ^a^	ϕ (%)^b^	ε (M^–1^ cm^–1^) at λ_max_
[Eu(**PhenDMA**)]^3+^	1.67 ± 0.02	0.816 ± 0.007	0.45	13.9 ± 0.4	31,720
[Eu(**Phenacetate**)]^+^	1.66 ± 0.02	0.615 ± 0.007	0.93	10 ± 1	25,297
[Eu(**Phencrypt**)]^3+^	1.38 ± 0.04	0.62 ± 0.01	0.76	4 ± 1	10,206
[Eu(**Phen18c6**)]^3+^	1.708 ± 0.001	0.28 ± 0.03	3.16	1.4 ± 0.1	32,958
[Eu(**BipyDMA**)]^3+^	1.629 ± 0.005	0.812 ± 0.004	0.43	1.3 ± 0.1^c^	15,927
				9 ± 2^b^	

a *q* = inner-sphere hydration number, calculated using the modified Horrocks method.

b Determined using gradient based method against tris­(dipicolinato)­europium­(III) (λ_ex_ = 279 nm, φ = 13.5 ± 1.5%).

c Referenced against quinine sulfate (λ_ex_ = 317 nm, φ = 54.6%).

We note that previous reports proposed an intermediate valence within europium–phenanthroline complexes, rationalized by a decreased Eu–N^phen^ bond distance compared to other reported Eu^2+^–N^aliph^ bond distances.^52^ However, our data does not support this notion. The Eu–N^phen^ bonds that we observe are consistently shorter than Eu–N^aliph^, and although the numerical values obtained for the Eu–N^phen^ bonds support the previous report, suggesting an intermediate valence, our structural and electronic characterization data (vide infra, electron paramagnetic resonance, EPR) do not support this notion.

Other reports of divalent lanthanide complexes, particularly ytterbium,^53^ have shown that electron transfer to bipyridine or phenanthroline can be characterized by X-ray crystallography.^54^ Thus, we synthesized and crystallized the bipyridine adduct [Eu­(**BipyDMA**)­I]I to investigate this effect. However, our data remains consistent with a divalent oxidation state of europium. An overlay of the [Eu­(**BipyDMA**)­I]I and [Eu­(**PhenDMA**)­I]I structures reveals near identical geometries, with the bipyridine Eu–N^bipy^ bond lengths elongated by ∼0.04 Å compared to the phenanthroline lengths (Figure S95).

### Spectroscopic and Electrochemical Characterization

To characterize the electronic properties of the complexes, we conducted X-band EPR spectroscopy at 15 K in frozen aqueous media. Although Eu^3+^ is EPR silent,^22,55,56^ Eu^2+^ is suitable for EPR due to its ^8^S_7/2_ ground state configuration, and slow electronic relaxation. The EPR spectra showed features with *g*-values ∼4.7 for both complexes (Figures S41 and S42). This value, while affirming the presence of the Eu^2+^ ion, deviates from the expected signal for the isotropic S-state ion of ∼2. This observation can be attributed to significant zero-field splitting of the Eu^2+^-containing complexes, and rhombicity induced by the low symmetry of the complexes that complicates the spectral features.

Cyclic voltammetry was performed in methanol using LiCl (100 mM) as the supporting electrolyte with analyte concentrations of ∼1 mM (for voltammograms, see Supporting Information, Figures S45–S48). Peak anodic potentials are reported against ferrocene/ferrocenium (Fc/Fc^+^) as an internal standard. Peak anodic potentials were more negative than the Eu^2+^ aqua ion (Figure 4).^52^ [Eu­(**Phencrypt**)]^2+^ exhibits the most positive peak anodic potential of the series (−0.83 V) and is in good correlation with a related, Eu-containing cryptand.^37,41,57^ The phenanthroline and bipyridine acetamide derivatives have a more negative peak anodic potential (−0.87 and −0.86 V, respectively, Figure 4).^52,58^ These results highlight, that both electronic and steric properties of ligands influence the redox potential of the corresponding Eu-containing complexes. Although these potentials are more negative than the free ion and are likely to be readily oxidized in normoxic tissues, they are not negative enough to reduce water; therefore, we hypothesized that the Eu^2+^ complexes would persist in hypoxic tumors, characterized by low oxygen content, to enable contrast enhancement in MRI.^59,60^

**Figure 4:**
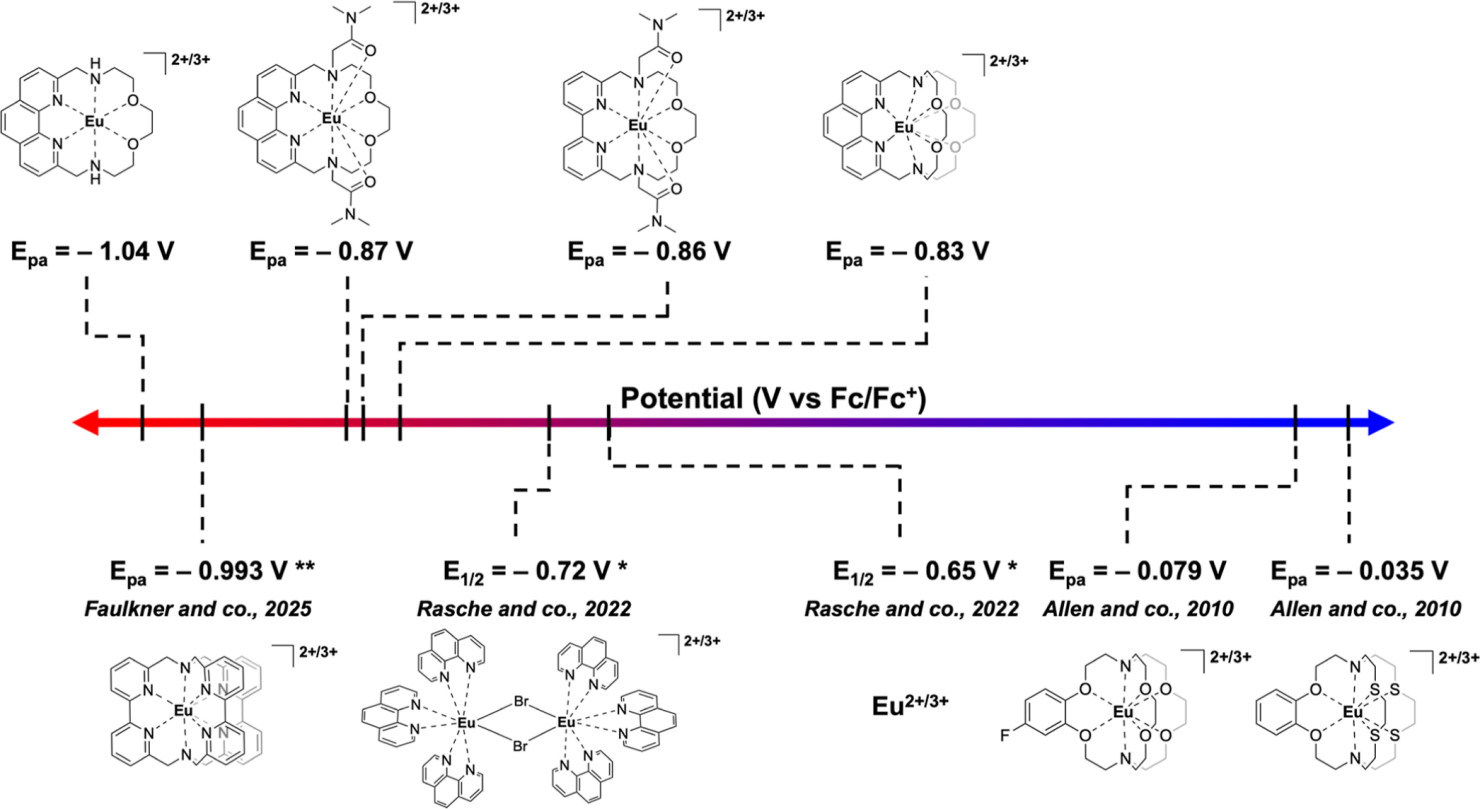
Summary of electrochemical data of the experimental compounds (top) compared with relevant previously reported compounds. Experimental compounds were measured against ferrocene/ferrocenium (Fc/Fc^+^) in a solution of LiCl (100 mM) in methanol. All experimentally determined potentials are reported relative to Fc/Fc^+^. Reported potentials were reported against Fc/Fc^+^ unless otherwise noted.* = approximated by converting from Ag/AgCl to Fc/Fc^+^.^50^** = approximated by converting from standard hydrogen electrode to Fc/Fc^+^.^50^

### Photophysical Characterization

Phenanthroline and bipyridine are known to sensitize lanthanide luminescence either as pendant, coordinating ligands or ternary coligands.^12^ Our ligand design incorporates the polypyridine ligand directly into the coordinating macrocycle.^61^ The UV–visible spectra of the phenanthroline compounds (λ_max_ = 279 nm) were invariant upon complexation, and the bipyridine chelates exhibited a 4200 cm^–1^ bathochromic shift (λ_max_ = 315 nm) compared to the free ligand.

To probe the luminescence properties of the trivalent complexes, emission spectra were collected and were normalized to the ^5^D_0_ → ^7^F_1_ band because this magnetic dipole transition is invariant to changes in the ligand field, enabling visualization of the change in the hypersensitive ^5^D_0_ → ^7^F_2_ transition (Figure 5A). The complexes were excited employing the π → π* transitions of phenanthroline (279 nm) and bipyridine (315 nm) to sensitize Eu^3+^ luminescence. All compounds displayed the ^5^D_0_→^7^F_0_ band that is specific to Eu^3+^ ions in C_s_, C_n_, or C_nv_ symmetries.^55^ This observation is consistent with the symmetry in the crystal structures.

**Figure 5:**
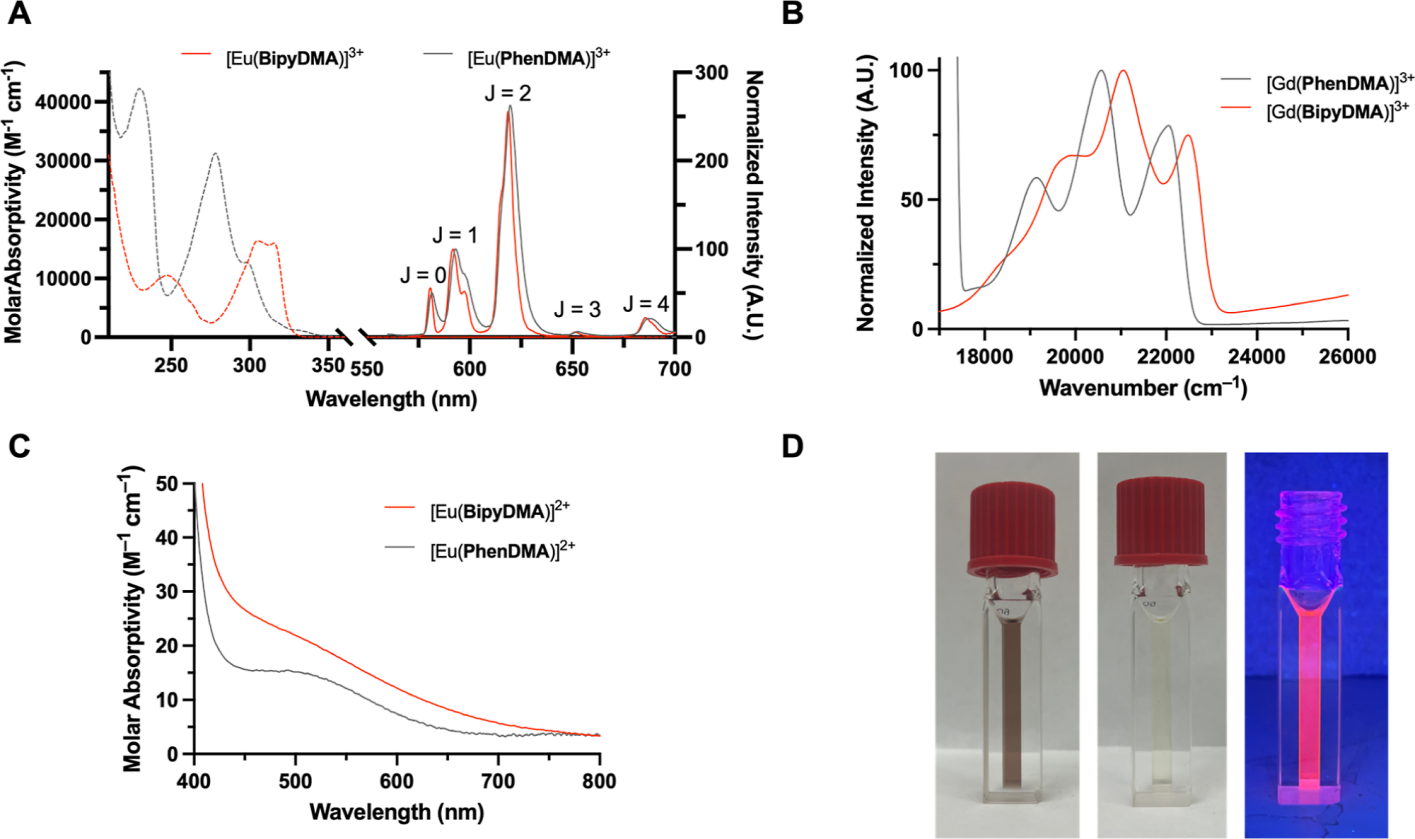
Photophysical characterization of trivalent and divalent lanthanide complexes. (A) Absorbance (dashed lines) spectra and steady-state emission (solid lines) spectra after excitation of the antenna (λ_ex_ = 279 and 315 nm for phenanthroline and bipyridine, respectively) in Tris buffer (10 mM, pH 7.4); (B) determination of triplet energy levels from the Gd^3+^ low-temperature emission spectra measured at 77 K in 3-(*N*-morpholino)­propanesulfonic acid (MOPS, 10 mM, pH 7.4)/glycerol (2:1). (C) Absorbance spectra showing the broad absorbance in the visible region of the divalent europium complexes. (D) Solution (∼3 mM) in methanol of [Eu­(BipyDMA)]^2+^ before (left) and after (middle) exposure to air and red luminescence emitted from the oxidized species under a hand-held UV lamp (right). A.U. = arbitrary units.

The inner-sphere hydration number, q, was determined by measuring the luminescence lifetimes in H_2_O and D_2_O and applying the Horrocks equation^62^

1 $$q=A\left[\frac{1}{\tau_{H_{2}O}}-\frac{1}{\tau_{D_{2}O}}\right]-B$$

where $\tau_{H_{2}O}$ is the luminescence lifetime in H_2_O, $\tau_{D_{2}O}$ is the luminescence lifetime in D_2_O, *A* is the proportionality constant, which is 1.2 ms for Eu^3+^ and *B* a correction for the deexcitation of the ^5^D_0_ excited state caused by solvent molecules in the second-coordination sphere, which is 0.25 ms^–1^ for Eu^3+^.

Although none of the Eu^3+^-containing crystal structures contain inner-sphere water, solution measurements suggest displacement of the halide by aqua ligands. Indeed, lifetime measurements indicate that the Eu^3+^-containing complexes with acetamide bearing ligands exhibit q values of 0.43 and 0.45, which would likely indicate that an equilibrium between a *q* = 0 and a *q* = 1 species in solution, which has been reported for similar functionalized 18-membered macrocycles.^63,64^

The [Eu­(**Phenacetate**)]^+^ complex has a *q* value of 0.93, which indicates one inner-sphere water. While this initially concludes that the decreased steric hindrance of the carboxylate donors compared to the dimethylacetamide donors increases the inner-sphere hydration, it is important to note that these empirically determined *q*-values are approximations from equations based on polyaminocarboxylates with an estimated 10% error.^61^ Furthermore, acetamide complexes may exhibit modulated luminescence lifetime properties that are not fully captured by the empirical Horrocks–Beeby methods. [Eu­(**Phencrypt**)]^3+^ has a comparable inner-sphere hydration with *q* = 0.78, comparable to previously reported lanthanide cryptates, and [Eu­(**Phen18c6**)]^3+^ has a *q* value of 3 that is consistent with the ligand with the fewest donor atoms (Table 2).

Quantum yields were determined to assess the sensitization of the antenna and quenching effects of the inner-sphere water-molecules by comparison with tris­(dipicolinato)­europium­(III) (ϕ = 13.5 ± 1.5%, λ_ex_ = 279 nm, Phen derivatives), and quinine (ϕ = 54.6%, λ_ex_ = 317 nm, for Bipy).^65^ The Phen-series shows quantum yields ranging from 1.4 to 13.9%, correlating well with increasing inner-sphere hydration that quenches luminescence via O–H oscillations of coordinated water (Table 2).

[Eu­(**BipyDMA**)]^3+^ exhibited the smallest quantum yield of the series (1.3 ± 0.1%). Low-temperature (77 K) phosphorescence spectra of [Gd­(**PhenDMA**)]^3+^ and [Gd­(**BipyDMA**)]^3+^ (Figure 5B) revealed triplet energies of 22,051 and 22,484 cm^–1^, respectively, eliminating the possibility that the small quantum yield arises from a discrepancy of triplet levels. Instead, we posit that referencing against quinine sulfate underestimates quantum yield due to a mismatch in emission profiles. Indeed, when quantum yield measurements were acquired with tris­(dipicolinato)­europium­(III) (ϕ = 13.5 ± 1.5%, λ_ex_ = 279 nm) as the reference, a quantum yield of 9 ± 2% was obtained for [Eu­(**BipyDMA**)]^3+^ (Table 2, Figures S40–S44).

In contrast to the near colorless, red-emissive Eu^3+^-containing complexes, Eu^2+^-containing complexes exhibit broad absorbance features that stretch into the visible region at wavelengths longer than 500 nm (Figure 5C), producing distinct, red–brown solutions in water/methanol. The corresponding absorbances exhibited molar absorptivity values of 11–280 M^–1^ cm^–1^ (Figures S24–S28). Emission spectra obtained using excitation wavelengths of 340 nm, in accordance with reported Eu^2+^-containing complexes,^52^ indicated a lack of Eu^2+^-based luminescence (Figures S6–S12). Upon exposure to air, the 4f–4f emissions of Eu^3+^ were recovered (Figure S12). Furthermore, the divalent complexes displayed no difference in their photophysical properties in the presence of different halides as counterions (Figure S8), affirming that 5d–4f emissions are not observed and thus quenched in this compound class.

### *T*_1_-Relaxivity and *T*_1_-Weighted Imaging

Longitudinal (*T*_1_) relaxation experiments were conducted at 1.4 T to determine the potential of Eu^2+^ complexes as contrast agents for MRI. To provide relevant relaxometrically active, oxidatively insensitive reference compounds, we probed the corresponding Gd^3+^-containing complexes. [Gd­(**PhenDMA**)]^3+^ exhibited the greatest relaxivity (3.17 mM^–1^ s^–1^, Figure S55) that compares well with small molecular Gd^3+^-containing complexes with *q* ≤ 1. [Eu­(**BipyDMA**)]^2+^ and [Eu­(**PhenDMA**)]^2+^ (relaxivity = 2.43 and 2.49 mM^–1^ s^–1^ respectively, Figure S55) displayed comparable relaxivity values. [Gd­(**BipyDMA**)]^3+^ exhibited the lowest relaxivity of the series, (1.07 mM^–1^ s^–1^, Figure S55). Because we determined near identical q values for the corresponding Eu^3+^-containing complexes, differences in inner-sphere hydration do not rationalize this discrepancy. However, we note that crystallographic characterization of [Eu­(**BipyDMA**)]^2+^ and [Eu­(**PhenDMA**)]^2+^ revealed elongation of the Eu–I bond for the bipy complex by 0.04 Å. This difference, if further amplified for the smaller ionic radius Gd^3+^, could rationalize the diminished relaxivity for both **BipyDMA** complexes in comparison with their **PhenDMA** analogues. We can expect that the lower charge density of Eu^2+^ compared to Gd^3+^ gives rise to faster water exchange rates but likely has a minor effect on the overall relaxivity.^66,67^ Further comparison between the relaxivity values of the Gd^3+^ and Eu^2+^-containing complexes is made complicated due to the redox-activity between the polypyridine ligands and Eu^2+^ that is not found for the Gd^3+^ analogues, however, further mechanistic insight is subject to future work.

Because these relaxivity values were considered suitable for MRI, we probed the efficacy in *T*_1_-weighted, in vitro imaging experiments. Eu^2+^ and Gd^3+^-containing complexes were prepared with concentrations ranging from 0.05 to 0.25 mM in MOPS buffer (10 mM, pH 7.4) and imaged at 4.7 T on a small animal MR scanner. Indeed, [Eu­(**BipyDMA**)]^2+^ and [Eu­(**PhenDMA**)]^2+^ enhanced contrast in *T*_1_-weighted imaging, with [Eu­(**BipyDMA**)]^2+^ producing comparable signal-to-noise (SNR) ratios to the Gd^3+^-containing analogues (Figure 6). Exposure to air results in complete disappearance of *T*_1_ enhancement within 30 min, as anticipated from oxidation of Eu^2+^ to Eu^3+^ (Figure 6).

**Figure 6:**
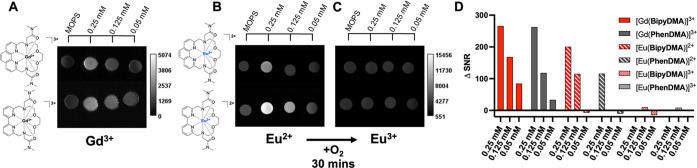
(A–C) Concentration gradient MR images acquired at 4.7 T of (A) [Gd­(**PhenDMA**)]^3+^ (top) and [Gd­(**BipyDMA**)]^3+^ (bottom); (B) divalent europium complexes [Eu­(**PhenDMA**)]^2+^ (top) and [Eu­(**BipyDMA**)]^2+^ (bottom); (C) divalent europium complexes after being exposed to air [Eu­(**PhenDMA**)]^3+^ (top), and [Eu­(**BipyDMA)**]­3+ (bottom). (D) Region-of-interest analysis quantifying the ΔSNR compared to the blank samples.

### Optical Imaging Experiments

The favorable luminescent properties of the Eu^3+^-containing complexes warranted optical imaging experiments. As such, we probed if in situ CR energy transfer (CRET) sensitization could produce detectable optical signals. Imaging experiments were performed using in situ excitation with [^68^Ga]­GaCl_3_ (∼10 μCi) in analogy to our established experimental workflows. Solutions containing [Eu­(**PhenDMA**)]^3+^ and [Eu­(**BipyDMA**)]^3+^ (0.1 to 50 nmol) were doped with [^68^Ga]­GaCl_3_ and imaged using a small animal imaging scanner with blocked excitation and collection of emission at 620 nm, monitoring the high intensity ^5^D_0_ → ^7^F_2_ emission band. Concurrent analysis of the nonemissive Gd^3+^-containing complexes was performed as a control experiment.

The optical imaging experiment revealed no visible luminescence from the Gd^3+^-containing complexes (Figure S65). In contrast, the Eu^3+^-containing complexes were emissive with signal above the limit of detection (LOD) between 1 and 5 nmol (0.005 and 0.025 mM, Figure 7). Despite the initially lower quantum yield measured for [Eu­(**BipyDMA**)]^3+^ in comparison with the phenanthroline analogue [Eu­(**PhenDMA**)]^3+^, the bipyridine complex exhibited no difference in LOD, further supporting validity of quantum yield values measured using the tris­(dipicolinato)­europium­(III) reference compound.

**Figure 7:**
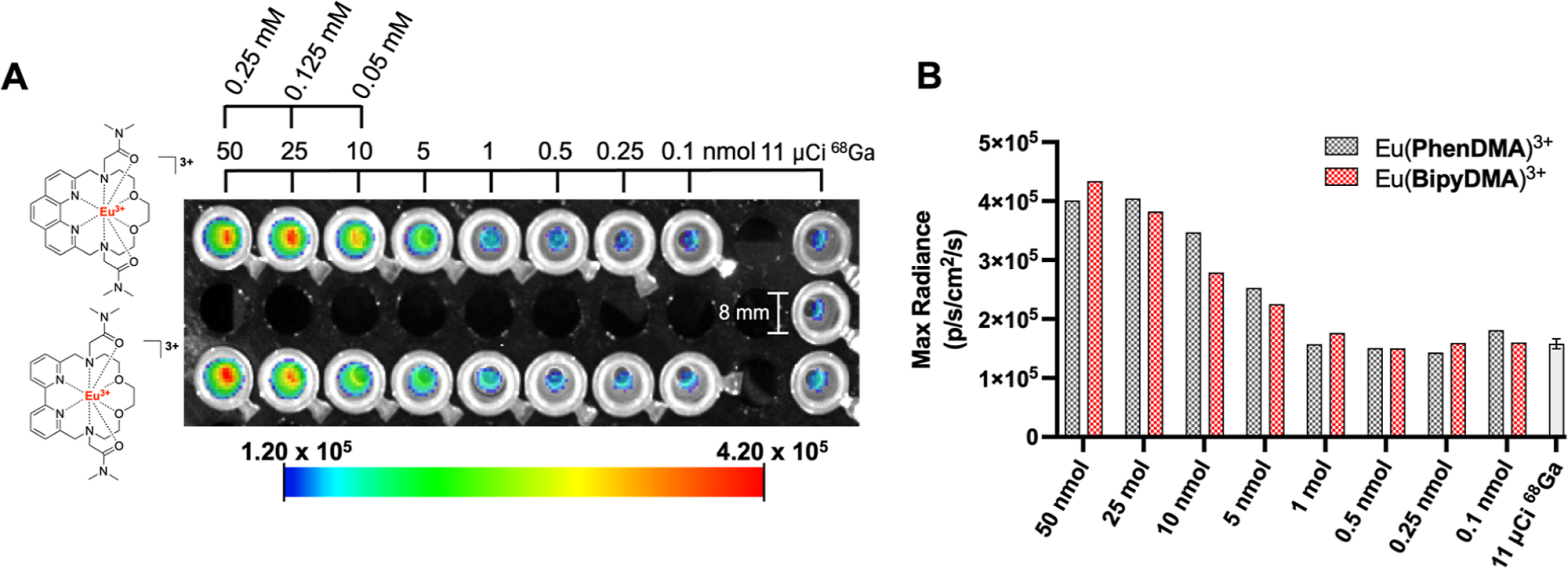
Demonstration of a multimodal imaging application. (A) In vitro image of [Eu­(**PhenDMA**)]^3+^ (top) with a well diameter of 8 mm. and [Eu­(**BipyDMA**)]^3+^ (bottom) in MOPS buffer (10 mM, pH 7.4) with [^68^Ga]­GaCl_3_ (11 μCi per well, 620 nm filter). (B) Region-of-interest analysis of CRET image, the region of interest were selected as the entire 8 mm well.

### In Vivo Validation of Bimodal Imaging

With in vitro optical and MR imaging experiments producing encouraging results, we determined that in vivo experiments with the [Eu­(**BipyDMA**)]^2+/3+^ pair were warranted. Employing mice with subcutaneous, RM1-PGLS prostate cancer xenografts on their right shoulder, we devised a sequential, bimodal imaging experiment using a single-dose administration of [Eu­(**BipyDMA**)]^2+^ (Figure 8A). To this end, [Eu­(**BipyDMA**)]^2+^ (1.0 mM, 100 μL, 100 nmol) was prepared under anoxic conditions and injected intratumorally following the precontrast, *T*_1_ weighted baseline scan at 4.7 T. Sequential, *T*_1_-weighted scans were performed in 4 min intervals immediately following injection. We observed bright, *T*_1_-weighted contrast in the lower half of the tumor (Figure 8B) that returned to background levels by 16 min post injection (Figure S66), indicating oxidation of the MR-active Eu^2+^ species, consistent with other in vivo reports of imaging with Eu^2+^.^30,31,33,68,69^ Subsequently, we administered [^68^Ga]­GaCl_3_ (40 μL, 52 μCi) as the in situ CRET source and conducted optical imaging (Figure 8). Efficient sensitization and emission of characteristic luminescence by the retained, the oxidized [Eu­(**BipyDMA**)]^3+^ complex was evident when compared to the [^68^Ga]­GaCl_3_ and saline controls (Figure S67).

**Figure 8:**
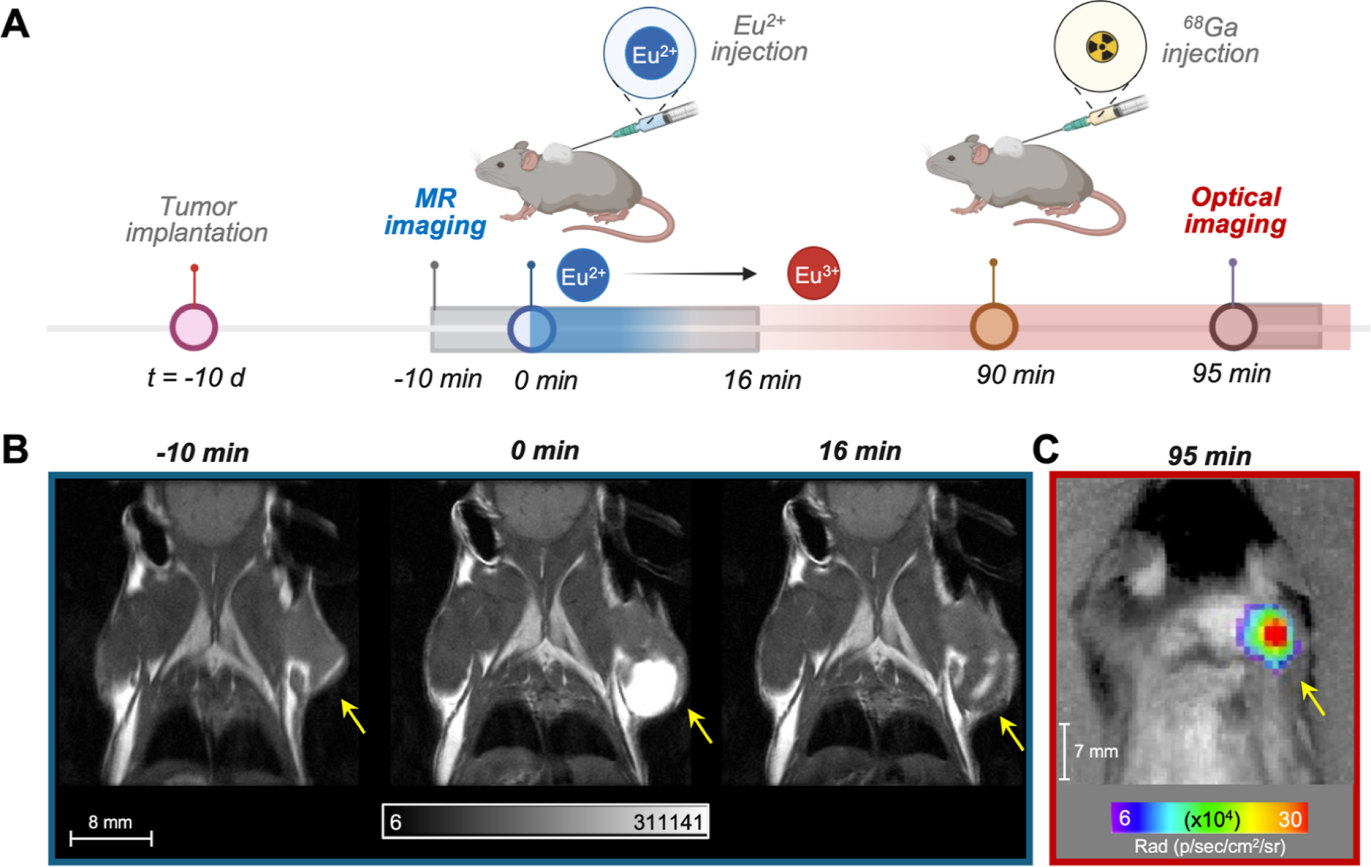
Demonstration of bimodal imaging application in a tumor-bearing mouse model using [Eu­(**BipyDMA**)]^2+/3+^. (A) Descriptive timeline of the animal study. (B) *T*_1_-weighted images preinjection (left) immediately after injection of [Eu­(**BipyDMA**)]^2+^ (middle), and 16 min after injection (right). (C) Luminescence imaging at 620 nm after administration of [^68^Ga]­GaCl_3_. Yellow arrows indicate the site of the RM-1 tumor xenograft, the tumor dimensions were 8.11 mm × 6.92 mm (∼194 mg).

## Conclusions

We designed, synthesized, and characterized an 18-membered macrocyclic crown either with flexible acetamide donor arms, **BipyDMA**, that coordinates Eu in both the +2 and +3 oxidation states. Although coordinating polypridine ligands quench 4f–5d luminescence emissions associated with Eu^2+^, crystallographic and EPR data affirm the presence of the divalent oxidation state. Notably, these states do not appear to perturb the magnetic and electrochemical properties of Eu^2+^ nor the photophysical properties of the Eu^3+^-containing complex.

The Eu^2+^-containing complex [Eu­(**BipyDMA**)]^2+^ produces relaxivities and *T*_1_-contrast enhancement comparable to Gd^3+^-based contrast agents, and the corresponding Eu^3+^-containing complex, [Eu­(**BipyDMA**)]^3+^, generates luminescence suitable for CRET-induced optical imaging at concentrations typical for Gd-enhanced MRI. A corresponding, single probe, bimodal imaging experiment in a mouse xenograft tumor model affirmed the in vivo feasibility of employing the [Eu­(**BipyDMA**)]^2+/3+^ complexes as a single dose contrast agent for sequential MRI and optical imaging.

Conclusively, our findings establish a framework in leveraging the Eu^2+/3+^ redox pair as a redox-responsive MRI/optical imaging agent for the noninvasive detection of hypoxic environments and persistence of chelate structure in both oxidation states in vivo. While [Eu­(**BipyDMA**)]^2+^ exhibits oxidation observable on the imaging time scale upon intratumoral injection, future work will focus on increasing the persistence of the Eu^2+^-containing complexes to allow for systemic injection and monitoring of redox status.

## Supplementary Material



## Notes

Detailed synthetic procedures, spectroscopic and spectrometric characterization, as well as detailed imaging procedures and controls can be found within the Supporting Information (PDF)
