# Supplementary material for: Single Molecule Eu2+/3+ Complex Platform for Optical and Magnetic Resonance Imaging In Vivo
Source: J Am Chem Soc. 2026 Jun 6;148(23):24520–30. doi: 10.1021/jacs.6c08257 (PMC13281538; doi:10.1021/jacs.6c08257)
Supplement: Supplementary file 1 [file ja6c08257_si_001.pdf]

Supporting Information for:

Single Molecule $Eu^{2+/3+}$ Complex Platform for Optical and Magnetic Resonance Imaging In Vivo.

Carter B. Rodgers,[a] Morgan P. Deal,[b] Leah C. Garman,[a] Ilia A. Guzei,[a] Matthew J. Allen,[b] and Eszter Boros*[a]

[a] Department of Chemistry, University of Wisconsin, 1101 University Avenue, Madison, Wisconsin 53706, United States E-mail: eboros@wisc.edu

[b] Department of Chemistry, Wayne State University, 5101 Cass Avenue, Detroit, Michigan 48202, United States

Table of Contents

1. General Procedures and Experimental Methods **2**

2. Synthesis of Ligands and Complexes **6**

3. Photophysical Characterization **12**

4. Electron Paramagnetic Resonance **32**

5. Cyclic Voltammetry **34**

6. Relaxivity Measurements **37**

7. T_1-weighted in vitro Images **38**

8. CRET Plate in vitro Images **42**

9. Bimodal *in vivo* Imaging **44**

10. NMR Spectra **46**

11. Solid-State Characterization **67**

12. LCMS Data **100**

13. Mass Spectrometry Data **103**

1. General Procedures and Experimental Methods

Electrochemical grade LiCl was purchased from Sigma Aldrich and used without further purification. Ferrocene was purified by sublimation. Methanol used for cyclic voltammetry was purified by refluxing 600 mL over magnesium turnings (5 g) activated with iodine (0.5 g), then distilling under an inert atmosphere before being stored over activated molecular sieves (3 Å) in the glovebox under an atmosphere of N_2. Solvents described as dry were either purchased as anhydrous or were stored in the glovebox over activated molecular sieves (3 Å).

All solvents used within air-sensitive applications were degassed by 3–5 freeze-pump-thaw cycles before being stored in the glovebox. Any solid samples were pumped in the antechamber for at least 16 h before being brought into the glovebox.

Tris buffer was prepared by dissolving Tris-HCl in water and pH adjusting using 1 M NaOH and 1 M HCl. Then water was added to obtain a total volume of 100 mL at a concentration of 100 mM. A ten-fold dilution was performed to obtain 10 mM buffer. 3-(N-morpholino)propanesulfonic acid (MOPS) buffer was prepared by dissolving MOPS in water and pH adjusting to pH 7.4 using aqueous NaOH (1 M) and HCl (1 M). Then water was added to obtain a total volume of 100 mL at a concentration of 100 mM. A ten-fold dilution with water was performed to obtain 10 mM buffer.

Cyclic voltammetry was performed using a standard three-cell unit composed of a glassy carbon working electrode, a Pt wire counter electrode, and a silver pseudo reference electrode. A Pine Research WaveNow^{XV} potentiostat was used. Blank scans were first acquired, then solutions of complexes in methanol were added to obtain final concentrations between 1 and 3 mM in 5 mL of total volume. Representative voltammograms were recorded at 200 mV/s. Ferrocene was then added and another scan at 200 mV/s was acquired to enable referencing to ferrocene/ferrocenium.

UV–visible data was collected on a NanoDrop 1C instrument (AZY1706045). Spectra were collected from 190 to 850 nm in quartz cuvettes with 1 cm path lengths.

ICP–OES analysis was used to determine europium and gadolinium concentrations throughout experimentation. Analysis was performed on an Agilent 5110 inductively coupled plasma optical emission spectrometer. A 6-point standard curve with minimum R^2 values of 0.999 were used to determine unknown metal concentrations.

High-resolution ESI mass spectrometry was performed at the University of Wisconsin–Madison Department of Chemistry Paul Bender Chemical Instrumentation Center (CIC) using a Thermo Scientific Q Exactive Focus Orbittrap MS system.

Luminescence measurements were collected on a Hitachi F-7100 FL spectrophotometer. Wavelength scans were collected by exciting at the appropriate wavelength for antenna-mediated excitation and minimization of scattering interference. Emission spectra were generally collected

from 300 to 800 nm with 1,200 s scan time, 0.05 s response, and photomultiplier tube voltage of 400–700 V.

Quantum Yield and Luminesence Lifetime Measurements

Quantum yields were determined with a relative method comparing to a known standard. The complex was prepared to absorbance value of 0.1 at the respective excitation wavelength, and an emission spectrum was collected. The solution was diluted and the absorbance and emission spectra was collected again. The dilution series was repeated from 0.1 to 0.03 abs, and the relationship between the absorbance and the integrated emission was determined using a linear regression in Graphpad Prism. The quantum yield was determined with equation 1:

$$\phi_u = \phi_{\mathrm{st}}(\frac{Grad_u}{Grad_{st}})(\frac{\eta_u^2}{\eta_s^2}) \quad \text{Eq. 1}$$

Where ϕ_u is the quantum yield of the unknown, ϕ_{st} is the quantum yield of the standard, $Grad_u$ is the slope of the unkown, $Grad_{st}$ is the slope of the standard, η_u is the refractive index of the unknown solution, and η_{st} is the refractive index of the solvent of the standard.

Quinine sulfate used for quantum yield standards was recrystallized three times from water then dried under vacuum to yield white needles. The quinine sulfate as a quantum yield reference was performed in aqueous H_2SO_4 (0.5 M, $\eta = 1.346$) with excitation wavelengths of either 316 or 350 nm depending on the compound being measured because either of these wavelengths gives the same result ($\phi = 54.6\%$.[1,2] $Eu(dpa)_3$ was used as a reference ($\phi = 13.5\%$) with an excitation wavelength of 279 nm in tris buffer (pH 7.4, $\eta = 1.33$) and was used as the reference for phenanthroline-containing compounds.

Lifetime values were determined by fitting the luminescence-decay curves with equation 2:

$$I_t = I_0 * e^{-\frac{x}{\tau}} \quad \text{Eq. 2}$$

Where I_t is the initial luminscence emission intensity, I_0 is the intensity at time $x = 0$, x is the time, and τ is the luminescence lifetime. Data was fit using GraphPad Prism. Inner-sphere hydration numbers were then calculated using Horrocks method:[3]

$$q = A[\frac{1}{\tau_{H_2O}} - \frac{1}{\tau_{D_2O}} - B] \quad \text{Eq. 3}$$

Where q is the inner-sphere hydration number, A is 1.2 ms^{-1} and B is 0.25 for Eu^{3+}.

X-band electron paramagnetic resonance (EPR) spectra were collected using a Bruker ELEXSYS E500 spectrometer. The sample temperatures for liquid He measurements were maintained at 15 K by an Oxford ESR 900 continuous-flow liquid He cryostat regulated by an Oxford ITC-503S temperature controller.

Nuclear Magnetic Resonance ^{1}H, ^{13}C, homonuclear correlation spectroscopy (COSY), heteronuclear single quantum coherence (HSQC), and heteronuclear multiple bond correlation (HMBC) experiments were performed at the University of Wisconsin-Madison Department of Chemistry Paul Bender Chemical Instrumentation Center using a Bruker Avance III 500 (Bender Fund) and a Bruker Avance III 400 (UW Madison Instructional Laboratory Modernization Award). Deuterated solvents ($CDCl_3$, D_2O, and DMSO-d_6) where purchased from Sigma Aldrich. Chemical shifts (δ) are reported in parts per million (ppm) and referenced to tetramethylsilane (0.00 ppm) for spectra collected in $CDCl_3$ or the residual solvent peaks for spectra collected in D_2O (4.79 ppm) or DMSO-d_6 (2.50 ppm). The apparent multiplicity is reported as "s" = singlet, "d" = doublet, "t" = triplet, and "q" = quartet.

Radioactive and Optical Imaging Experiments ^{68}Ga was received from the University of Wisconsin Radiopharmaceutical Production Facility from a $^{68}Ge/^{68}Ga$-Generator and eluted in aqueous HCl (0.1 M, pH 2).

In vitro Image Assays and images were recorded on an IVIS Lumina series III from Caliper LifeSciences or Lago optical imaging system from Spectral Instruments Imaging small animal imager. The collection time was five minutes. All wells were doped with Cerenkov emitter (10 mL) resulting in a total volume of 200 µL. Image analysis was completed with Aura software.

Magnetic Resonance Imaging (MRI) was performed by the University of Wisconsin Carbone Cancer Center's (UWCCC) Small Animal Imaging and Radiotherapy Facility (SAIRF) using a horizontal bore, 4.7 T MRI, small-animal scanner (Agilent, Palo Alto, CA, USA, 20 cm diameter bore). T_1-weighted, fast-spin-echo (FSE) images (TR/TE_{eff} = 520/11 ms, 90° flip angle, matrix 196 × 196, FOV 35 mm × 15 mm, and slice thickness 1.0 mm) were acquired in coronal plane using a 38 mm diameter radio-frequency volume coil.

Reverse-Phase Purification Methods
Chromatographic Methods

Method A: Gradient: 0–1 min: 5% B; 1–2 min: 5–20% B; 2–7 min: 20–60% B; 7–11 min: 60% B; 11 min: 95% B; 11–12 min: 95% B; 12 min: 5% B; 12–14 min: 5% B. A = water + trifluoroacetic acid (TFA, 0.1%); B = acetonitrile + TFA (0.1%).

Method B: Gradient: 0–3 min: 5% B; 3–4min: 5–15% B; 4–20 min: 15–65% B; 20–21 min: 65–95% B; 21–25 min: 95% B; 25–27 min: 95–5% B; 27–30 min: 5% B. A = water + TFA (0.1%); B = acetonitrile + TFA (0.1%).

Method C: Gradient: 0–3 min: 5% B; 3–4min: 5–15% B; 4–20 min: 15–65% B; 20–21 min: 65–95% B; 21–25 min: 95% B; 25–27 min: 95–5% B; 27–30 min: 5% B. A = water + formic acid (0.1%); B = acetonitrile + formic acid (0.1%).

C18 Seppak Cartridges were purchased from Waters™. Cartridges were conditioned with 5 mL of acetonitrile, followed by 5 mL of 50/50 acetonitrile/water, then 5 mL of water. Product was loaded and first eluted with 2 mL of water followed by increasing concentrations of acetonitrile.

Liquid Chromatography–Mass Spectrometry (LC–MS) was performed on a Phenomenex Luna C18 column (5 µm, 150 mm × 3 mm, 100 Å, AXIA packed) at a flow rate of 0.8 mL/min using a single quadrupole Agilent 1200 Infinity II LC/MSD system equipped with a binary gradient pump, UV–visible detector, automatic injector, and an atmospheric pressure electrospray ionization (AP-ESI) source. Ultraviolet absorption was recorded at 220 and 254 nm, and positive and negative mass spectra were collected from m/z = 100 to 1,500. LC–MS was used to monitor reaction progress and for low resolution mass spectrometry (LR-MS).
Method A: Gradient: 0–3 min: 5% B; 3–4 min: 5–20% B; 4–9 min: 20–60% B; 9–13 min: 60% B; 13–13.5 min: 5% B: 13.5–16 min: 5% B. A = water + formic acid (0.1%); B = acetonitrile + formic acid (0.1%).

Method B: Gradient: 0–3 min: 10% B; 3–10 min: 10–90% B; 10–13 min: 90% B; 13–13.5 min: 90–10% B; 13.5–16 min: 10% B. A = aqueous ammonium formate (10 mM); B = acetonitrile.

Method C: Gradient: 0–3 min: 5% B; 3–10 min: 5–95% B; 10–12 min: 95% B; 12–12.5 min: 95-5% B; 12.5–16 min: 5% B. A = water + formic acid (0.1%); B = acetonitrile + formic acid (0.1%).

2. Synthesis of Ligands and Complexes

General Schematic for Phenanthroline Containing Compounds

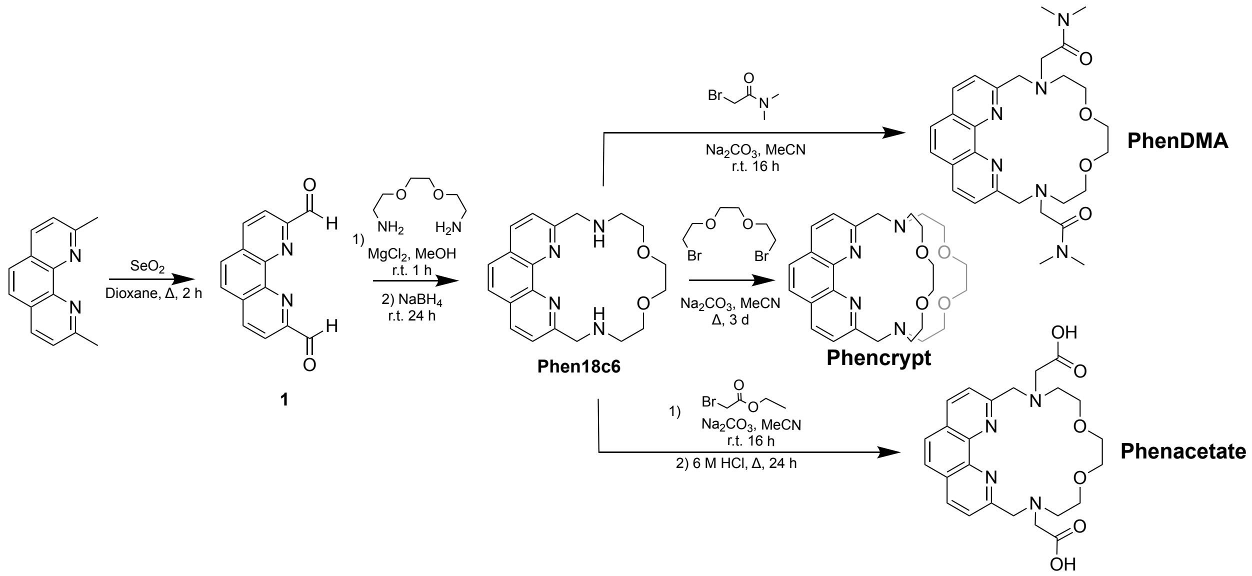

Scheme S1 – Synthesis of ligand precursors and title ligands.

Synthesis of Phenanthroline dialdehyde (1)

Selenium dioxide (5.95 g, 53.6 mmol) was brought to reflux in a mixture of dioxane (200 mL) and water (10 mL). A solution of neocuproine (5.00 g, 24.0 mmol) in dioxane (50 mL) was added dropwise over the course of 2 h. The red reaction mixture was heated at reflux for an additional 2 h before being filtered while hot through hot celite. The filtrate was cooled in an ice bath for 4 h, and the resulting solid was filtered and rinsed with diethyl ether. To separate selenium impurities, the product was redissolved in dimethylformamide (~ 80 mL) and left to stand for 24 h. The resulting black solid was removed by filtration, and chilled water (~ 40 mL) was added dropwise to the orange filtrate causing the formation of a yellow precipitate that was collected by filtration and dried under reduced pressure to yield **1** (yield: 2.394 g, 41%) as a yellow solid. NMR matched previous reports.[4] ^{1}H NMR (500 MHz, DMSO-d_6) δ 10.36 (s, J = 0.8 Hz, 1H), 8.80 (d, J = 8.2, 0.8 Hz, 1H), 8.32 (d, J = 8.2 Hz, 1H), 8.30 (s, 1H). ^{13}C NMR (126 MHz, DMSO) δ 194.20, 152.69, 145.76, 138.92, 131.94, 129.73, 120.62.

Synthesis of Phen18c6

Under an atmosphere of N_2, dialdehyde **1** (277 mg, 1.17 mmol) and anhydrous $MgCl_2$ (122 mg, 1.19 mmol) were suspended in dry methanol (30 mL), and the mixture was stirred for 45 min. To the resulting golden solution was added the diamine (174 µL, 1.19 mmol). Stirring continued for 1 h then $NaBH_4$ (218 mg, 5.76 mmol) was added slowly as gas evolved. The reaction was stirred for 25 h. The reaction was quenched with H_2O (20 mL), then the solvent was concentrated to 20 mL under reduced pressure. The suspension was transferred to a separatory funnel and extracted with dichloromethane (3 × 30 mL). The combined organic layers were dried over $MgSO_4$. $MgSO_4$ was removed by filtration through celite, and solvent was evaporated under reduced pressure to afford a yellow solid (yield: 0.318 g, 77%) that was stored in the glovebox freezer without further purification due to it being slightly air sensitive. NMR matched previous reports.[5] ^{1}H NMR (400 MHz, $CDCl_3$) δ 8.16 (d, *J* = 8.1 Hz, 2H), 7.73 (s, 2H), 7.50 (d, *J* = 8.2 Hz, 2H), 4.23 (s, 4H), 3.79–3.71 (m, 4H), 3.69 (s, 4H), 3.02–2.95 (m, 4H). ^{13}C NMR (126 MHz, $CDCl_3$) δ 159.48, 145.69, 136.30, 127.68, 125.82, 122.34, 70.73, 70.67, 55.64, 49.47.

Synthesis of Phen Bisacetamide (PhenDMA)

Under an atmosphere of N_2, Phen18c6 (97 mg, 0.28 mmol) was added to a 20-mL scintillation vial and dissolved in dry acetonitrile. Oven-dried Na_2CO_3 (0.090 g, 0.83 mmol) was added, and the reaction mixture was stirred for 5 min before 2-bromo-N,N-dimethylacetamide (0.050 mL, 0.46 mmol) was added. The reaction was stirred for 16 h, filtered, and solvent was evaporated under reduced pressure. The crude residue was purified using reverse-phase chromatography using **Method A**, affording a yellow oil that was triturated with Et_2O in the vial. Ether was removed with a pipette, and the resulting white solid was dried under reduced pressure (yield: 40 mg, 28%). ^{1}H NMR (400 MHz, D_2O) δ 8.71 (d, *J* = 8.3 Hz, 2H), 8.13 (d, *J* = 1.7 Hz, 2H), 7.97 (d, *J* = 8.3 Hz, 2H), 5.03 (s, 4H), 4.49 (s, 4H), 3.93 (t, *J* = 4.5 Hz, 4H), 3.75 (t, *J* = 3.9 Hz, 4H), 3.60 (s, 4H), 2.93 (s, 6H), 2.82 (s, 6H). ^{13}C NMR (101 MHz, D_2O) δ 165.27, 150.59, 141.81, 140.18, 129.36, 127.48, 124.15, 69.88, 64.67, 59.19, 55.76, 55.27, 35.86, 35.24 ESI–HRMS: $[M+H]^+$ = calc. 523.3027, found: 523.3027; $[M+Na]^+$ = calc. 545.2847, found: 545.2842.

Synthesis of Phen bisacetate (Phenacetate)

Under an atmosphere of N_2, phen18c6 (220 mg, 0.62 mmol) was dissolved in dry acetonitrile (10 mL). Oven-dried Na_2CO_3 (395 mg, 3.73 mmol) and the yellow slurry were combined and the resulting reaction mixture was stirred for 10 min before adding ethyl bromoacetate (0.150 mL, 1.36 mmol). The reaction was stirred for 20 h. The reaction was filtered through a 0.45 µm PTFE syringe filter, and solvent was removed from the yellow filtrate under reduced pressure to afford a wet yellow solid. The crude mixture was purified by reverse phase chromatography using **Method A** to afford a yellow solid that was heated at reflux in aqueous HCl (6 M, 20 mL) for 24 h. Solvent was evaporated under reduced pressure and the residue was purified by reverse phase chromatography using **Method B**. The purified fractions were combined and evaporated, then

redissolved in dichloromethane (10 mL) three times to remove residual trifluoroacetic acid, the final product was dissolved in H_2O and lyophilized to afford a colorless powder (yield: 113 mg, 39% yield over the two steps). ^{1}H NMR (400 MHz, D_2O) δ 8.76 (d, J = 8.3 Hz, 1H), 8.12 (d, J = 1.9 Hz, 1H), 8.00 (d, J = 8.4 Hz, 1H), 5.09 (s, 2H), 4.03 (s, 2H), 3.93–3.86 (m, 2H), 3.76–3.69 (m, 2H), 3.64 (s, 2H). ^{13}C NMR (126 MHz, D_2O) δ 170.30, 150.28, 141.44, 140.21, 129.61, 127.63, 124.86, 117.50, 115.19, 69.91, 64.68, 58.30, 56.13, 55.92. ESI-HRMS: $[M+H]^+$ = calc. 469.2082, found: 469.0292.

Synthesis of Phencrypt

To an oven-dried roundbottom flask (500 mL) was added oven-dried Na_2CO_3 (306.7 mg, 2.894 mmol), phen18c6 (100 mg, 0.3 mmol), and dry acetonitrile (250 mL). The reaction was brought to reflux then 1,2-bis(2-bromoethoxy)ethane (85.2 mg, 0.309 mmol) in acetonitrile (25 mL) was added dropwise via syringe pump over the course of 16 h. The yellow solution was heated at reflux for an additional 4 days, then cooled to ambient temperature and filtered. Solvent was evaporated under reduced pressure to afford a dark-yellow, wet solid. The crude product was passed through a neutral alumina column (dichloromethane→60/40 dichloromethane/acetonitrile) to afford a slightly yellow powder (yield: 26.2 mg, 19%). NMR matched previous reports.[6] ^{1}H NMR (400 MHz, $CDCl_3$) δ 8.29 (d, J = 8.2 Hz, 2H), 7.80 (s, 2H), 7.61 (d, J = 8.2 Hz, 2H), 4.07 (s, 4H), 3.76–3.50 (m, 17H), 2.75 (m, 8H). ^{13}C NMR (101 MHz, $CDCl_3$) δ 158.71, 145.39, 137.63, 128.18, 126.22, 123.66, 68.56, 66.23, 60.15, 53.12.

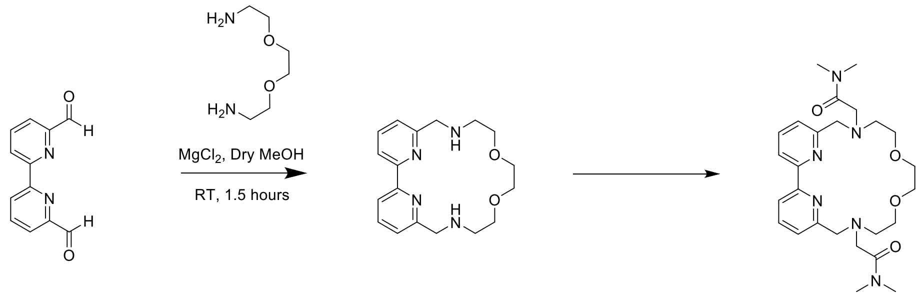

Synthesis of Bipy18c6

The compound was synthesized according to a published procedure.[7] Under an atmosphere of N_2, 2,2'-bipyridine-6,6'-dicarbaldehyde (272 mg, 1.28 mmol) and anhydrous $MgCl_2$ (122 mg, 1.28 mmol) were suspended in dry methanol (20 mL) and stirred for 30 min. To the resulting mixture was added 1,8-diamino-2,6-dioxaoctane (190 mL, 1.3 mmol). The clear and faint yellow reaction was stirred for 1 h before $NaBH_4$ (240 mg, 6.4 mmol) was added slowly followed by stirring for 16 h. The reaction was quenched by the addition of water (20 mL), and the solvent was concentrated to 20 mL under reduced pressure. The product was extracted with

dichloromethane (3 × 35 mL), and the combined organic layers were dried over $MgSO_4$ and filtered through celite. Solvent was evaporated under reduced pressure to afford a faint yellow oil (yield: 331 mg, 79%). The product was stored in the glovebox freezer due to being slightly air sensitive. NMR matched reported spectra.[8]

Synthesis of BipyDMA

Under an atmosphere of N_2, bipy18c6 (112.6 mg, 0.3429 mmol) was dissolved in dry acetonitrile (7 mL). Di-isopropylethylamine (179 µL, 1.03 mmol) was added followed by 2-bromo-*N,N*-dimethylacetamide (74 µL, 0.69 mmol). The solution was stirred for 4 h, then solvent was evaporated under reduced pressure, and the resulting residue was purified using basic alumina (acetonitrile→55/45 acetonitrile/H_2O) affording the product as a white powder (yield: 43 mg, 25%) ^{1}H NMR (400 MHz, D_2O) δ 7.96–7.83 (m, 4H), 7.39 (d, *J* = 7.1 Hz, 2H), 3.92 (s, 4H), 3.57 (s, 4H), 3.39 (d, *J* = 6.2 Hz, 8H), 2.91 (s, 6H), 2.87 (s, 4H), 2.81 (s, 6H). ^{13}C NMR (101 MHz, D_2O) δ 154.65, 138.75, 123.77, 120.64, 68.80, 67.24, 60.30, 55.11, 36.14, 35.16. ESI–HRMS: $[M+H]^+$ = calc. 499.3027, found: 499.3017; $[M+Na]^+$ = calc. 521.2847, found: 521.2847.

Synthesis of Metal Complexes

Eu(Phen18c6)I_2
Phen18c6 (69.0 mg, 0.196 mmol) was dissolved in dry acetonitrile (3 mL) , EuI_2 (80.1 mg, 0.197 mmol) was dissolved in acetonitrile (1 mL). The two solutions were mixed, and a red-brown precipitate formed that was collected by filtration. The solid was rinsed with acetonitrile (3 mL) then dried under reduced pressure affording a red-brown solid in quantitative yield.

Eu(Phen18c6)Br_2
Phen18c6 (62.5 mg, 0.177 mmol) and $EuBr_2$ (55.4 mg, 0.178 mmol) were dissolved in dry acetonitrile with a drop of methanol to aid in dissolution of $EuBr_2$. A deep red color formed, and the solution was left to stand for 30 min before a red-brown precipitate formed that was collected by filtration, rinsed with acetonitrile (3 mL), and dried under reduced pressure to yield a red-brown solid (yield: 80.4 mg, 67%).

[Eu(Phencrypt)(H_2O)$_2$]I_2
Phencrypt (9.9 mg, 0.021 mmol) was dissolved in acetonitrile (500 µL) with a drop of methanol for solubility. A EuI_2 stock in acetonitrile (518 µL, 40.9 mM, 0.0212 mmol) was added to the solution of ligand, and the resulting dark-orange solution was stirred for 2 h. Tetrahydrofuran (5 mL) was added, and the resulting reaction mixture was left to stand for 24 h in a –20 °C freezer. The solid was collected, then dark-red crystals suitable for X-ray diffraction were grown from tetrahydrofuran diffused into a methanolic solution (yield: 8.0 mg, 61%).

Eu(PhenDMA)I_2
PhenDMA (21.5 mg, 41.1µmol) was dissolved in dry acetonitrile (1 mL) and mixed with a

solution of EuI_2 in acetonitrile (64 mM, 640 µL, 40.0 mmol) resulting in the formation and redissolving of a dark red precipitate. The resulting solution was filtered and placed under vapor diffusion with tetrahydrofuran to afford maroon crystals suitable for X-ray diffraction. A few crystals were separated for characterization, and the remaining mother liquor was removed by decanting. The solids were dried under reduced pressure to yield a maroon solid (yield: 24.6 mg, 75%).

Eu(BipyDMA)I_2

BipyDMA (22.1 mg, 44.3µmol) was dissolved in dry acetonitrile (1 mL), and EuI_2 (18.0 mg, 44.3 µmol) was added, immediately forming a dark-brown solution. The solution became cloudy with the addition of tetrahydrofuran and was evaporated under reduced pressure. The red oily solid was resuspended in tetrahydrofuran (1 mL) with a few drops of acetonitrile to aid in dissolution and was layered under hexanes. Single crystals suitable for X-ray diffraction formed over the course of a week. The dark purple crystals were isolated, and the supernatant was decanted, the remaining solid was dried under reduced pressure to yield a dark red solid (yield: 9.8 mg, 28%).

[Eu(PhenDMA)Br]OTf_2

PhenDMA (10.4 mg, 19.9µmol) was dissolved in methanol (2 mL), and to the solution was added a solution of $Eu(OTf)_3$ in methanol (164 mM, 121 µL, 19.7 µmol) and was immediately placed under vapor diffusion with tetrahydrofuran. Colorless crystals suitable for X-ray diffraction grew over the course of two days. (yield: 6.3 mg, 40%).

[Eu(PhenDMA)Br]Br_2

To a solution of **PhenDMA** in H_2O (14.6 mM, 103 µL, 1.50 µmol) was added a solution of $EuBr_2$ in H_2O (15.79 mM, 95.0 µL, 1.50 µmol), and the resulting solution was diluted to a total volume of 1,500 µL with MOPS buffer (10 mM, pH 7.4) and stirred for 15 min to afford the product in quantitative yield. LC–MS (**Method A**): retention time = 2.60 min. ESI–HRMS: $[EuL \cdot 2Br]^+$ = calc. 833.0533, found: 833.1975.

[Eu(BipyDMA)Br]Br_2

To a solution of **BipyDMA** in H_2O (55.1 mM, 1.50 mL, 82.7 µmol) was added a solution of $EuBr_2$ in H_2O (87.1 mM, 949 µL, 82.7 µmol), the solution was stirred for 15 mins and then concentrated to 0.5 mL under reduced pressure before being purified via reverse phase chromatography (**Method C**). The product was then lyophilized to afford a light-yellow powder. (yield: 51.0 mg, 85%). LC–MS (**Method A**): retention time = 1.27 min. ESI-HRMS: $[EuL \cdot (CHO_2)]^{2+}$ = calc. 348.1072, found. 348.1066.

Eu(phenacetate)Br

Phenacetate (32.0 mg, 68.3 µmol) was dissolved in methanol (5 mL) and mixed with $EuBr_3$ (26.8 mg, 68.3 µmol). Bright red luminescence was observed when illuminated under a UV light. The product was precipitated with diethyl ether (3 mL), supernatant was removed, and the

resulting white powder was dried under reduced pressure for 4 h affording a white powder (yield: 35.0 mg, 83%). LC–MS (**Method A**): retention time = 5.80 min. ESI-HRMS: $[Eu \cdot L]^+$ = calc. 617.1045, found: 617.1032.

Eu(phen18c6)Br$_3$

Phen18c6 (10.0 mg, 28.4 μmol) and $EuBr_3$ (17.0 mg, 34.1 μmol) was suspended in dry acetonitrile (15 mL) and brought to reflux under an atmosphere of N_2 for 20 h. The reaction mixture was cooled to ambient temperature then filtered to remove solids. The filtrate was concentrated to 3 mL under reduced pressure, then the product was precipitated upon addition of Et_2O. The product was collected as a white powder that exhibited red luminescence under UV light (yield: 8.0 mg, 50%). LC–MS (**Method B**): retention time = 1.65 min. ESI–HRMS: $[EuL \cdot 2Br]^+$ = calc. 664.9481, found: 664.9414.

[Eu(phencrypt)Br]Br$_2$

Phencrypt (17.9 mg, 38.4 μmol) was dissolved in dry acetonitrile (10 mL), and $EuBr_3$ (21.1 mg, 42.2 μmol) was added. The reaction was heated at reflux under an atmosphere of N_2 for 16 h. The reaction was cooled to ambient temperature and filtered and evaporated, affording a yellow residue. The residue was redissolved in 50/50 acetonitrile/water (0.5 mL) and purified through a C18 Seppak cartridge, the desired fractions were combined and freeze dried to afford a yellow powder (yield: 10.3 mg, 43%). Single crystals suitable for X-ray diffraction grew from vapor diffusion of tetrahydrofuran into a methanolic solution. LC–MS (**Method A**): retention time = 1.04 min; LR-MS: calc. $[M \cdot 2Br]^+$ = 777.0 found. 777.0.

Complexation Procedure for Divalent Complexes used in Relaxivity and T_1-weighted Imaging Experiments

In the glovebox, solutions of (**PhenDMA**) and (**BipyDMA**) were prepared to a known concentration (9.47–17.5 mM) in MOPS buffer (10 mM, pH 7.4). Separately, an aqueous solution of $EuBr_2$ (13.49 mM) was prepared. The metal and ligand were combined in a 1.0/1.0 molar ratio then diluted to a total volume of 1,000 μL at a final concentration of 1.0 mM of Eu. The resulting red solutions were stirred for 20 min before being serially diluted for further analysis.

[Gd(PhenDMA)]$^{3+}$

A solution of **PhenDMA** in H_2O (14.89 mM, 1,900 μL, 28 μmol) was mixed with a solution of $GdCl_3$ in H_2O (117.2 mM, 241.0 μL, 28.25 μmol). The reaction was stirred for 16 h then concentrated under reduced pressure before being purified via semi-preparative HPLC using **method B**. The desired fraction was concentrated under reduced pressure to afford a white solid (yield: 11.4 mg, 56%); LC–MS (**Method C**): retention time = 2.63 min; ESI–HRMS: $[GdL \cdot 2(C_2O_2F_3)]^+$ = calc. 906.1896, found: 906.1832.

[Gd(BipyDMA)]$^{3+}$

A solution of **BipyDMA** in H_2O (74.8 mM, 500.0 µL, 37.4 µmol) was mixed with an aqueous solution of $GdCl_3$ (117.2 mM, 319.0 µL, 37.39 µmol), and the resulting reaction mixture was stirred for 16 h then purified through a C18 SepPak cartridge. The desired fractions were concentrated under reduced pressure to afford a white solid (yield: 20.6 mg, 72%). LC–MS (**Method A**): retention time = 1.24 min. ESI-HRMS: $[GdL \cdot 2Cl]^{+}$ = calc. 726.1573, found. 726.1523; $[GdL \cdot Cl]^{2+}$ = calc. 345.5942, found. 345.5920.

3. Photophysical Data

Luminescence Spectra

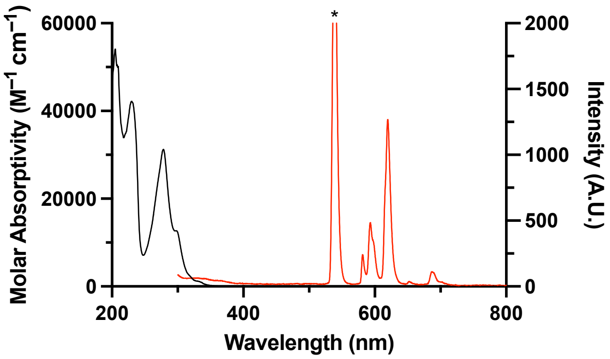

Figure S1 – Absorption (black trace) and emission spectrum (λ_{ex}: 279 nm, orange trace) of [Eu(**PhenDMA**)]$^{2+}$ (2.90 µM) in tris buffer (10 mM, pH 7.4). (* = second-order excitation peak)

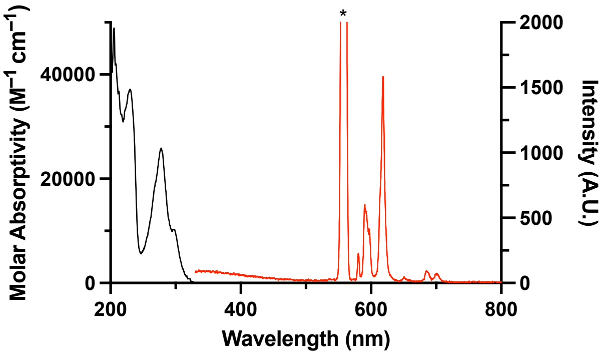

Figure S2 – Absorption (black trace) and emission spectrum (λ_{ex}: 279 nm, orange trace) of [Eu(**Phenacetate**)]$^{+}$ (3.71 µM) in tris buffer (10 mM, pH 7.4). (* = second-order excitation peak)

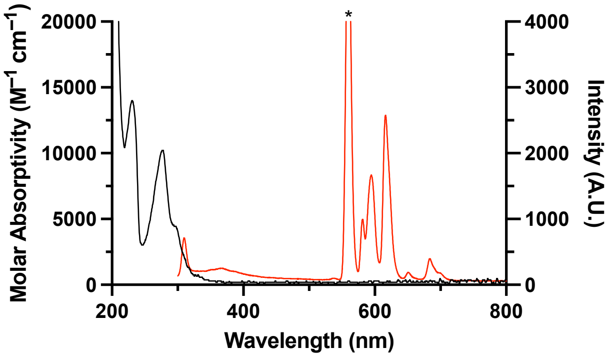

Figure S3 – Absorption (black trace) and emission spectrum (λ_{ex}: 279 nm, orange trace) of [Eu(**Phencrypt**)]$^{3+}$ in tris (10 mM, pH 7.4). (* = second-order excitation peak)

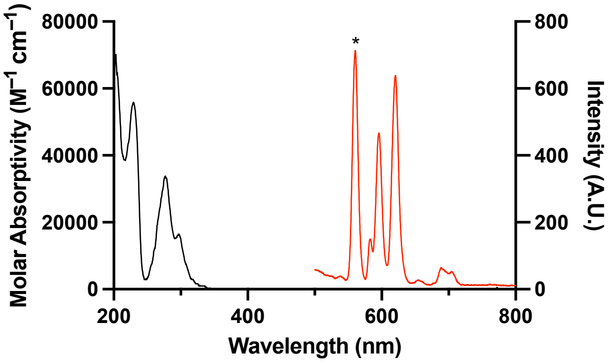

Figure S4 – Absorption (black trace) and emission spectrum (λ_{ex}: 279 nm, orange trace) of [Eu(**Phen18c6**)]$^{3+}$ (3.10 μM) in tris buffer (10 mM, pH 7.4). (* = second-order excitation peak)

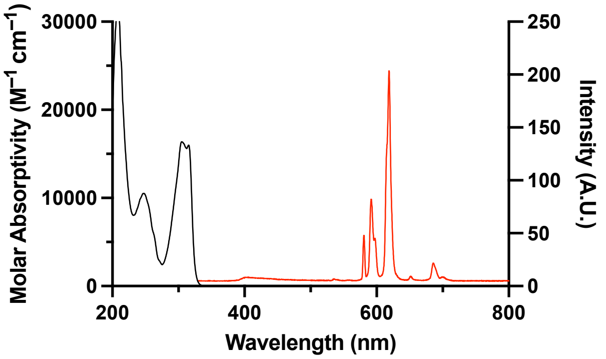

Figure S5 – Absorption (black trace) and emission spectrum (λ_{ex}: 316 nm, orange trace) of [Eu(**Bipydma**)]$^{3+}$ (5.46 μM) in tris buffer (10 mM, pH 7.4).

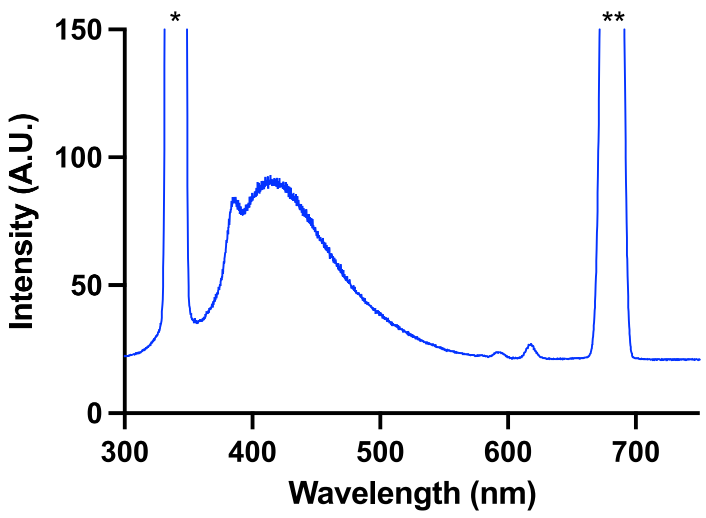

Figure S6 – Emission Spectra of [Eu(**PhenDMA**)]$^{2+}$ in methanol using λ_{ex} = 340 nm as a reasonable excitation for Eu(II) compounds. (* = first-order excitation peak; ** = second-order excitation peak). The peak centered at 430 nm is ligand based phosphoresence. *4f–4f* transitions are ~600 nm.

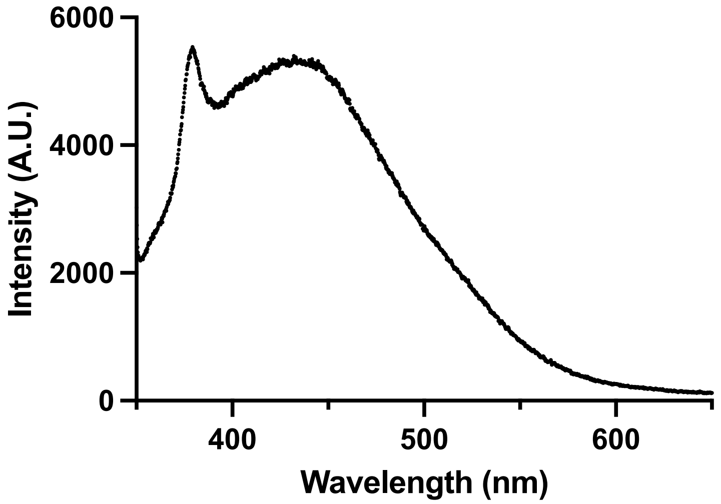

Figure S7 – Emission spectra of [Eu(**BipyDMA**)]$^{2+}$ in methanol with λ_{ex} = 340 nm, broad peak centered at ~430 nm is consistent with the ligand phosphorescence.

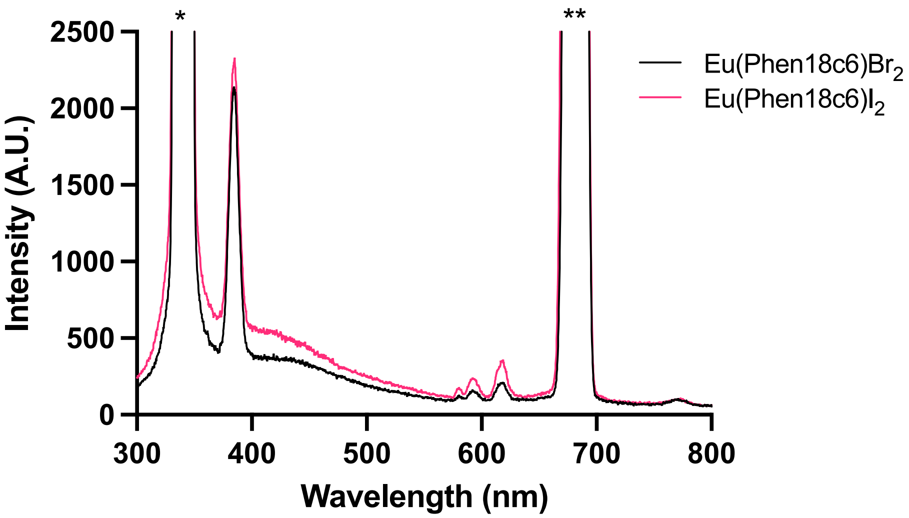

Figure S8 – Emission spectra of a solution of Eu(**Phen18c6**)Br_2 (2 mM) in methanol (Black line) and a solution of Eu(**Phen18c6**)I_2 (2 mM) in methanol (pink line) using λ_{ex} = 340 nm. The peaks near 380 and 400 nm are consistent with ligand fluorescence. *4f–4f* transitions are at ~620 nm. (* = first-order excitation peak; ** = second-order excitation peak)

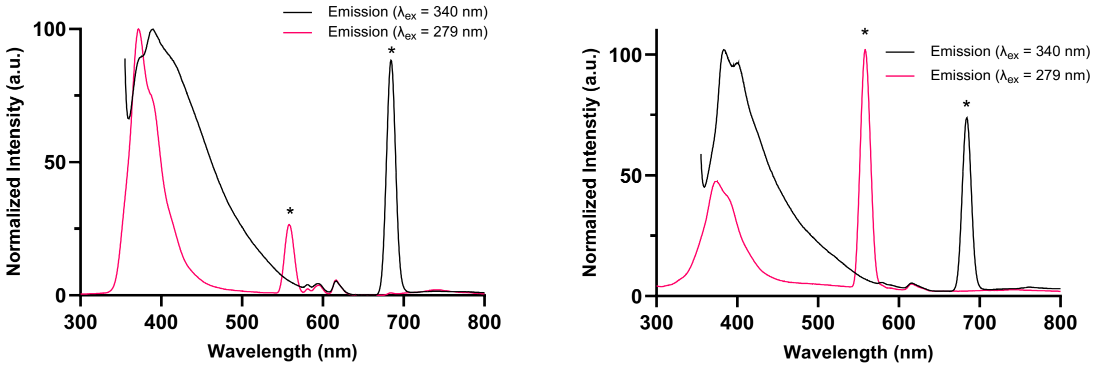

Figure S9 – Normalized emission spectra of [Eu(**Phencrypt**)]$^{2+}$ in H_2O (left) and methanol (right) at various excitation wavelengths, λ_{ex} = 279 (pink) to excite the phenanthroline antenna and λ_{ex} = 340 nm (black) to excite *4f–5d* transitions. (* = second-order excitation peak)

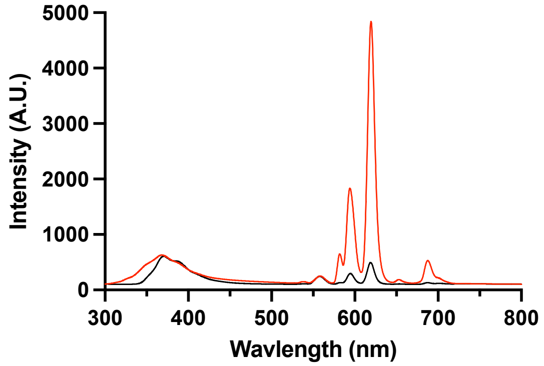

Figure S10 – Emission spectra to observe *4f–4f* transitions of a solution (1.0 mM) of [Eu(**PhenDMA**)]$^{2+}$ (black line) in MOPS buffer (10 mM, pH 7.4) compared to a solution (0.25 mM) of [Eu(**PhenDMA**)]$^{3+}$ (red line) with a λ_{ex} = 279 nm.

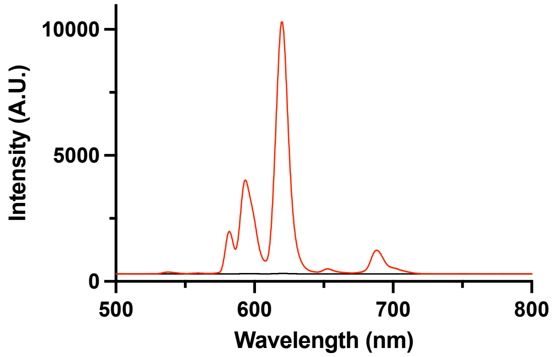

Figure S11 – Emission spectra cropped to 500–800 nm to observe *4f–4f* transitions of a solution (1.0 mM) of [Eu(**BipyDMA**)]$^{2+}$ (black line) in MOPS buffer (10 mM, pH 7.4) compared to a solution (1.0 mM) of [Eu(**BipyDMA**)]$^{3+}$ (red line) with a λ_{ex} = 315 nm.

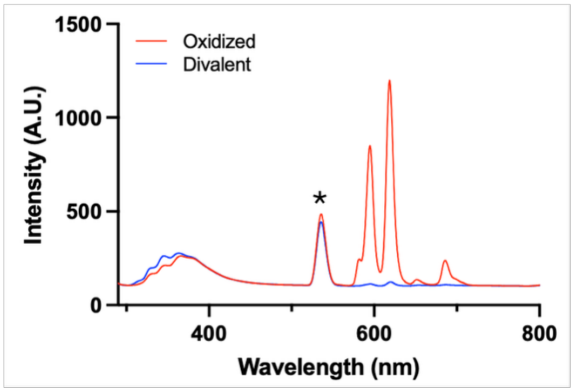

Figure S12 – Emission spectra of [Eu(**Phencrypt**)]$^{2+}$ (2 mM) in methanol before (blue) and after (red) exposure to air using a λ_{ex} = 279 nm to sensitize the antenna, showing the quenched luminescence in the divalent state and restored luminescence upon oxidation. Ligand fluorescence at 380 nm remains invariant. (* = second-order excitation peak)

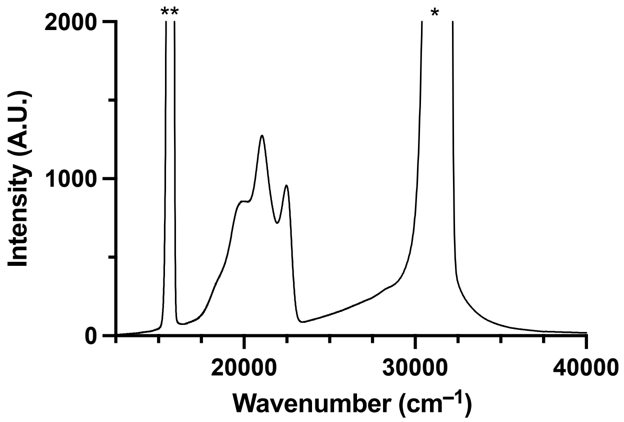

Figure S13– Low-temperature (77 K) emission spectra (λ_{ex} = 317 nm) of [Gd(**BipyDMA**)]$^{3+}$ in a 2:1 mixture of MOPS buffer (10 mM, pH 7.4) and glycerol. (* = first-order excitation peak; ** = second-order excitation peak)

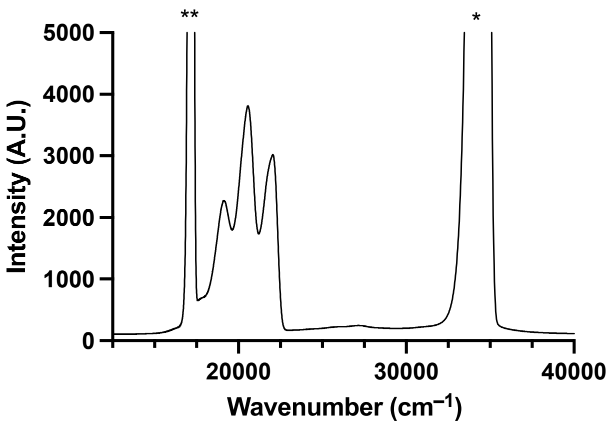

Figure S14– Low-temperature (77 K) emission spectra (λ_{ex} = 279 nm) of [Gd(**PhenDMA**)]$^{3+}$ in a 2:1 mixture of MOPS buffer (10 mM, pH 7.4) and glycerol. (* = first-order excitation peak; ** = second-order excitation peak)

Ligand and Complex Molar Absorptivity Determination

To determine the concentration and molar absorptivity of each ligand used for subsequent experiments, spectrophotometric titrations were performed with Cu^{2+}. The formation of each copper–ligand complex was monitored at 304 or 321 nm using a cuvette (1 cm path length) and a NanoDrop spectrophotometer. In a total volume of 1,000 µL, ligand solutions were titrated with aliquots (10 µL, 10 nmol) of Cu^{2+} (as determined by ICP-OES). The titration endpoint was determined by the inflection point of the absorbance intensity at 304 or 320 nm because that point is diagnostic of complex formation. Standard curves were generated using known ligand concentrations to determine the molar absorptivity at λ_{max}. Slopes of the standard curves were determined using a linear regression in Graph Pad Prism. Molar absorptivity was calculated from the slope of the appropriate standard curve using the Beer–Lambert law.

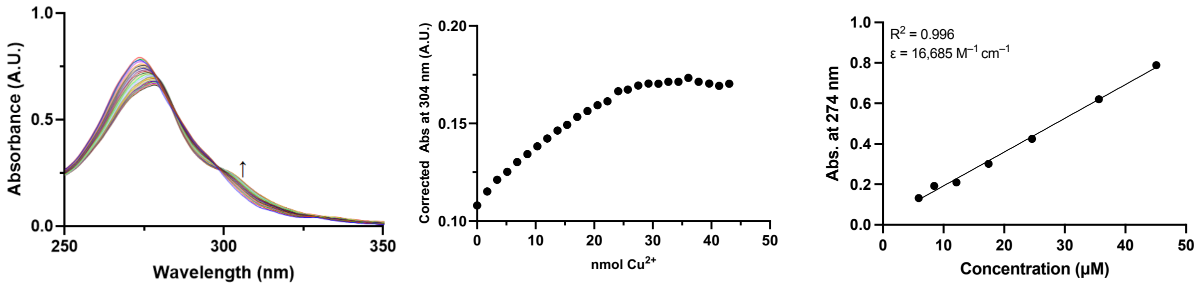

Figure S15 – Spectrophotometric ligand titrations with Cu^{2+}. UV–visible absorbance spectra of **PhenDMA** upon addition of Cu^{2+} (left) and UV–visible titration to endpoint monitored at 304 nm to determine ligand concentration (middle). Standard curve for molar absorptivity determination at 274 nm (right).

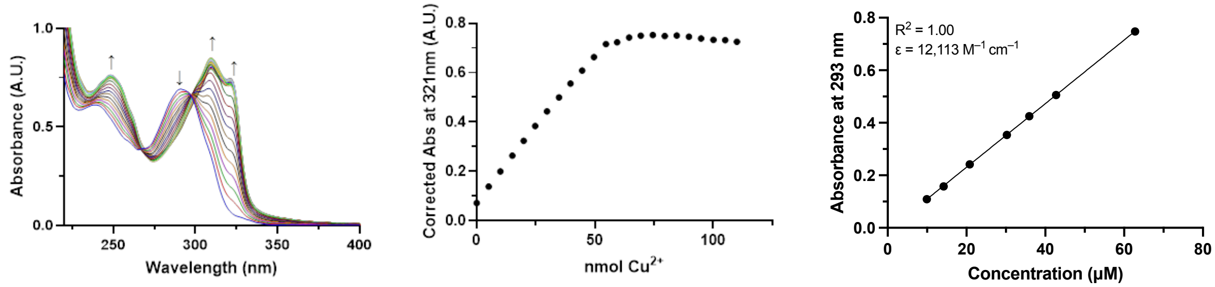

Figure **S16** – Spectrophotometric ligand titrations with Cu^{2+}. UV–visible absorbance spectra of **BipyDMA** upon addition of Cu^{2+} (left) and UV–visible titration to endpoint monitored at 321 nm to determine ligand concentration (middle). Standard curve for molar absorptivity determination at 293 nm (right).

To determine metal complex molar absorptivities, europium or gadolinium concentrations were determined using ICP–OES, then dilution series were performed in either tris buffer (10 mM, pH 7.4) or MOPS buffer (10 mM, pH 7.4). Slopes were determined using a linear regression in Graph Pad Prism. Molar absorptivities were calculated as the slopes of the appropriate standard curves using the Beer–Lambert law.

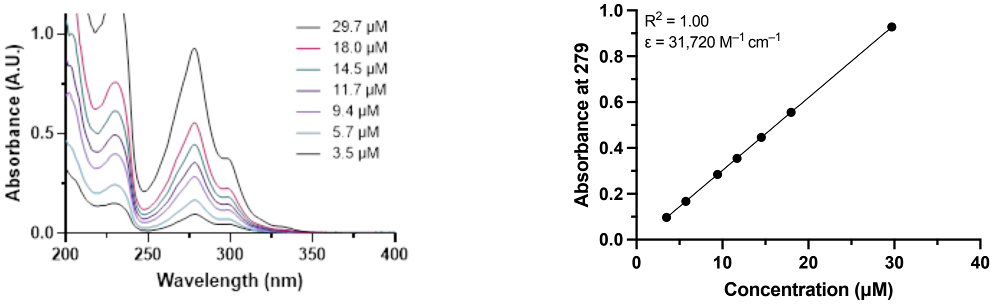

Figure S17 – UV–visible absorbance spectra of [Eu(**PhenDMA**)]$^{3+}$ (left) and standard curve for molar absorptivity determination at 279 nm (right).

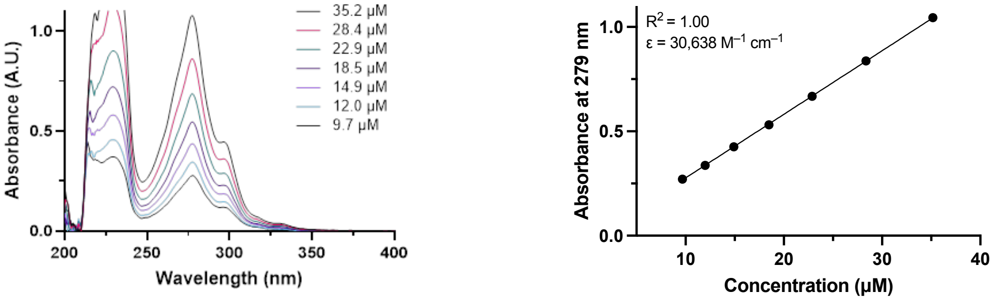

Figure S18 – UV–visible absorbance spectra of [Gd(**PhenDMA**)]$^{3+}$ (left) and standard curve for molar absorptivity determination at 279 nm (right).

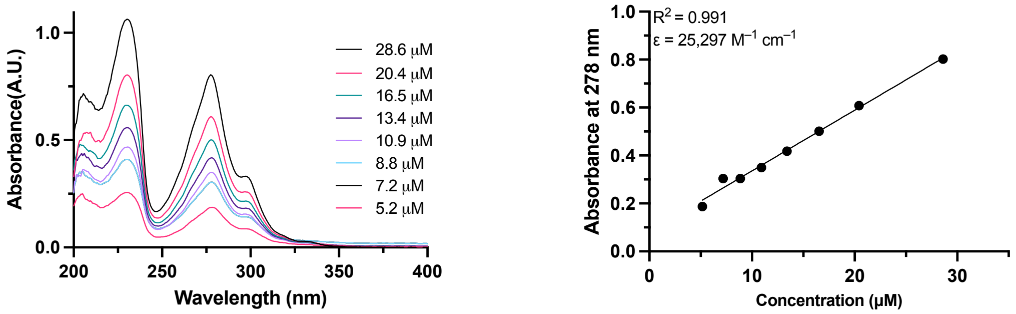

Figure S19 – UV–visible absorbance spectra of [Eu(**phenacetate**)]$^{+}$ (left) and standard curve for molar absorptivity determination at 279 nm (right).

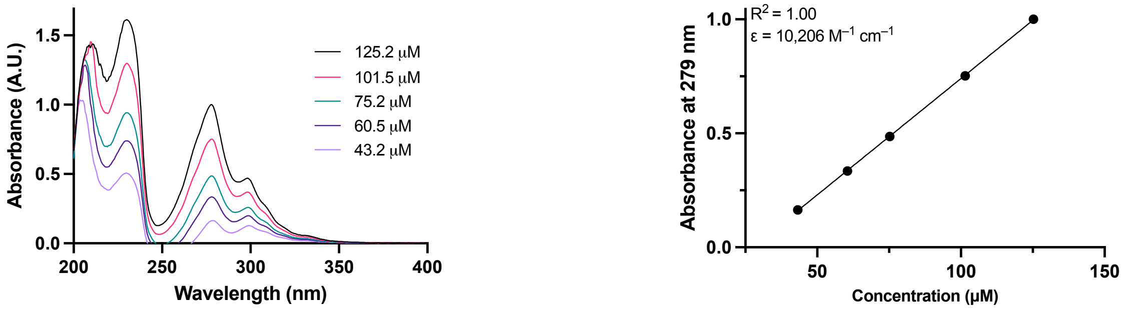

Figure S20 – UV–visible absorbance spectra of [Eu(**Phencrypt**)]$^{3+}$ (left) and standard curve for molar absorptivity determination at 279 nm (right).

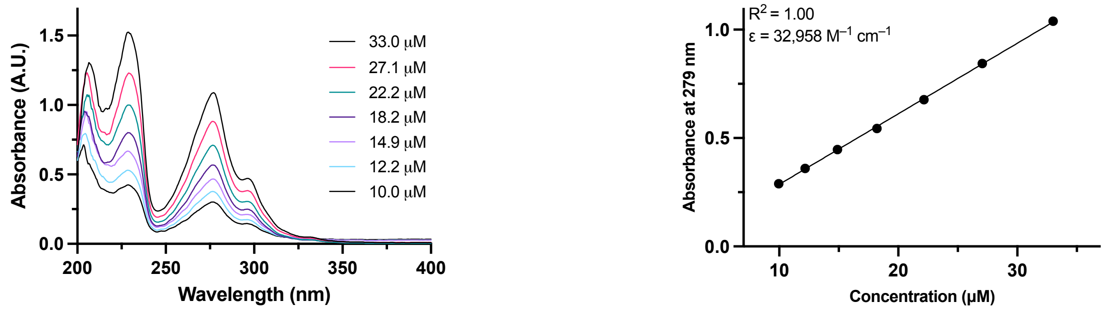

Figure S21 – UV–visible absorbance spectra of [Eu(**Phen18c6**)]$^{3+}$ (left) and standard curve for molar absorptivity determination at 279 nm (right).

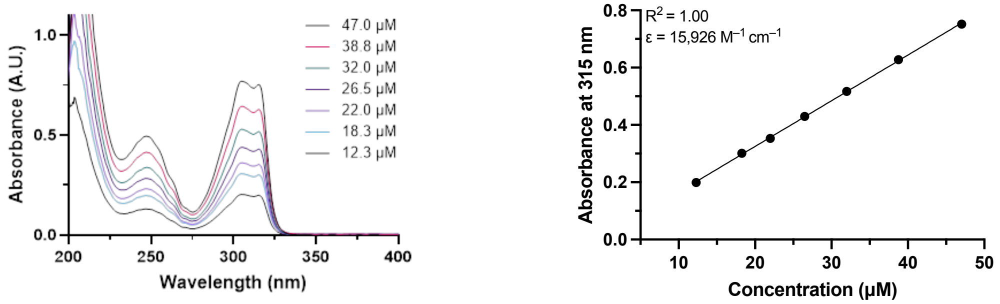

Figure S22 – UV–visible absorbance spectra of [Eu(**BipyDMA**)]$^{3+}$ (left) and standard curve for molar absorptivity determination at 315 nm (right).

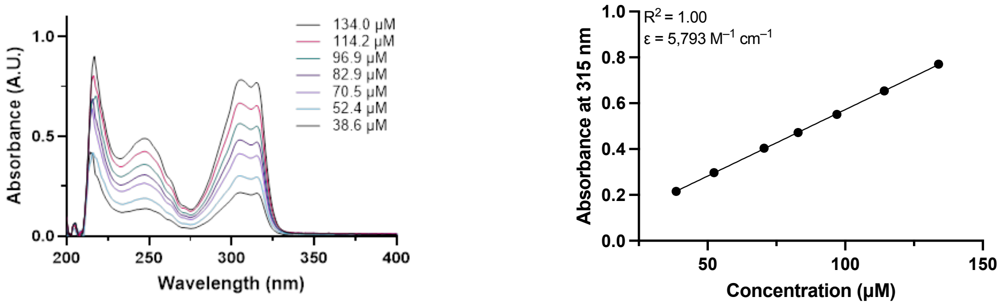

Figure S23 – UV–visible absorbance spectra of [Gd(**BipyDMA**)]$^{3+}$ (left) and standard curve for molar absorptivity determination at 315 nm (right).

Divalent Complex Molar Absorptivity Determination

To determine the molar absorptivity of metal complexes, europium concentration was determined using ICP–OES, then a Beer's Law plot was constructed to obtain the molar absorptivity from the slope of the curve.

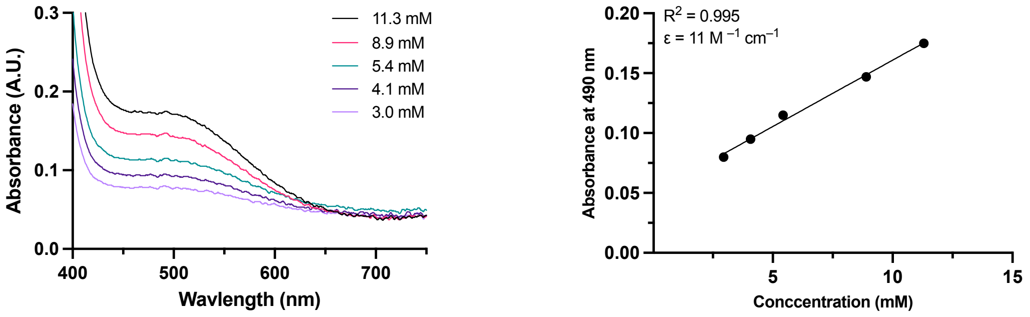

Figure S24 – UV–visible absorbance spectra of [Eu(**PhenDMA**)]$^{2+}$ (left) and standard curve for molar absorptivity determination at 490 nm (right) in MOPS buffer (10 mM, pH 7.4).

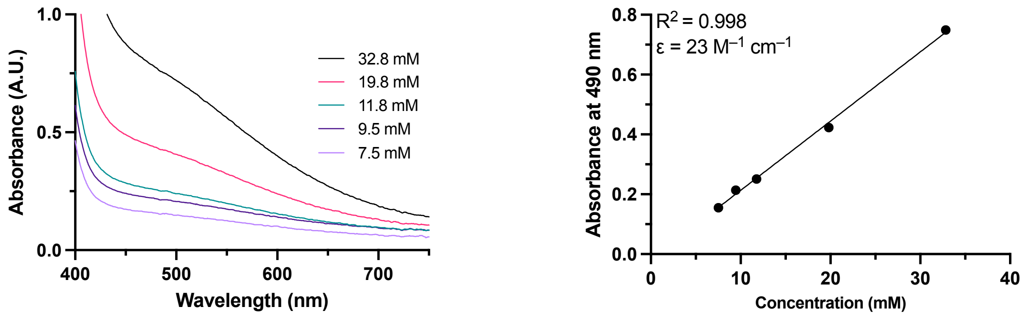

Figure S25 – UV–visible absorbance spectra of [Eu(**BipyDMA**)]$^{2+}$ (left) and standard curve for molar absorptivity determination at 490 nm (right) in MOPS buffer (10 mM, pH 7.4).

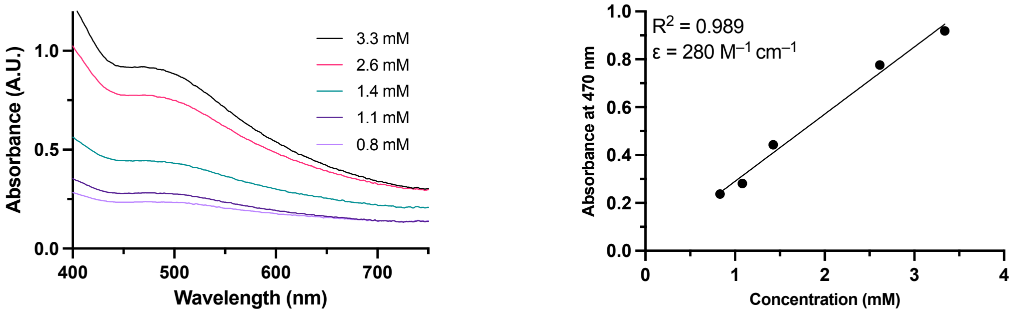

Figure S26 – UV–visible absorbance spectra of [Eu(**Phen18c6**)]$^{2+}$ (left) and standard curve for molar absorptivity determination at 470 nm (right) in methanol.

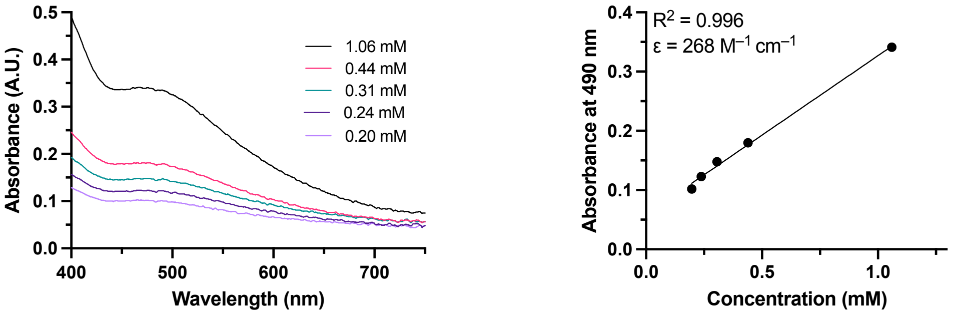

Figure S27 – UV–visible absorbance spectra of [Eu(**Phen18c6**)]$^{2+}$ (left) and standard curve for molar absorptivity determination at 470 nm (right) in MOPS buffer (10 mM, pH 7.4).

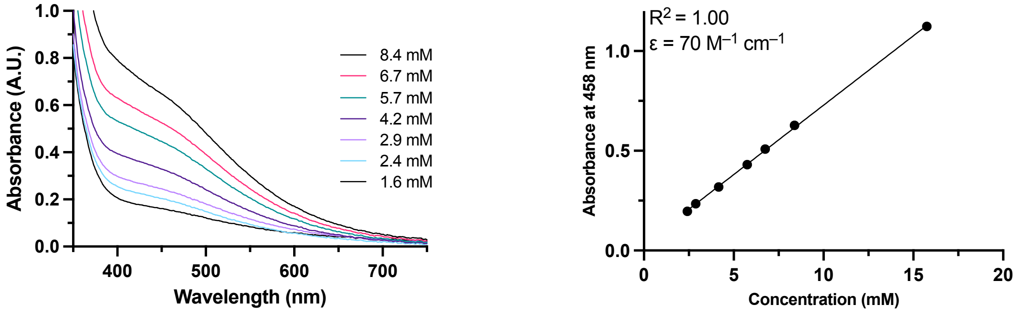

Figure S28 – UV–visible absorbance spectra of [Eu(**Phencrypt**)]$^{2+}$ (left) and standard curve for molar absorptivity determination at 470 nm (right) in methanol.

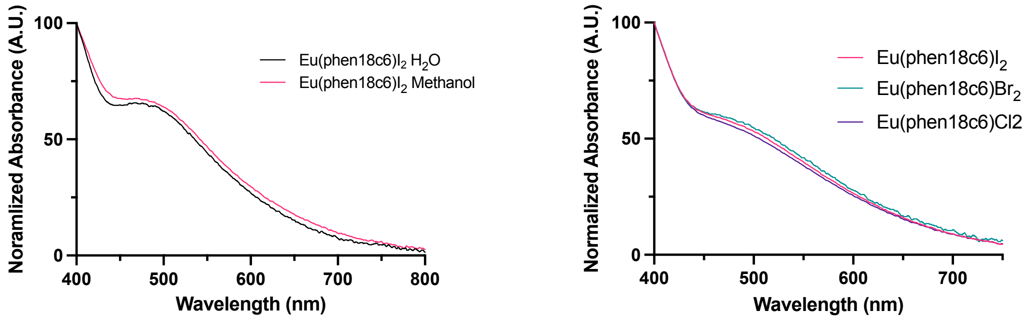

Figure S29 – Normalized absorbance spectra of (left) [Eu(**Phen18c6**)]$^{2+}$ in H_2O in methanol, showing no visible shift in absorbance band upon solvent change.

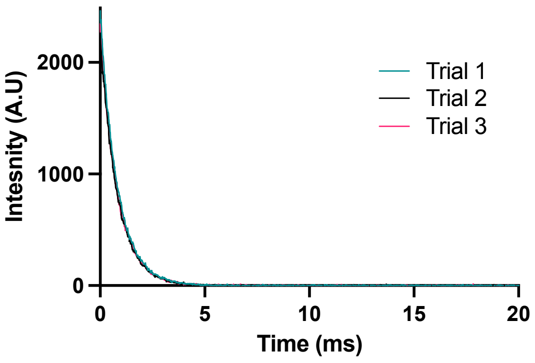

	Nonlin fit<br>Table of results	Trial 1	Trial 2	Trial 3
1	**One phase decay**			
2	**Best-fit values**			
3	Y0	2446	2241	2318
4	Plateau	-0.04114	-1.051	-0.2470
5	K	1.221	1.219	1.238
6	Half Life	0.5677	0.5686	0.5599
7	Tau	0.8191	0.8204	0.8078
8	Span	2446	2242	2318
9	**95% CI (profile likelihood)**			
10	Y0	2443 to 2449	2238 to 2244	2315 to 2321
11	Plateau	-0.3379 to 0.2556	-1.400 to -0.7010	-0.6141 to 0.1200
12	K	1.219 to 1.223	1.216 to 1.221	1.235 to 1.241
13	Half Life	0.5668 to 0.5687	0.5675 to 0.5698	0.5587 to 0.5611
14	Tau	0.8177 to 0.8204	0.8187 to 0.8221	0.8060 to 0.8095
15	**Goodness of Fit**			
16	Degrees of Freedom	1998	1998	1998
17	R squared	0.9997	0.9994	0.9994
18	Sum of Squares	76512	106228	117355
19	Sy.x	6.188	7.292	7.664
20	**Constraints**			
21	K	K > 0	K > 0	K > 0
22				
23	**Number of points**			
24	# of X values	2001	2001	2001
25	# Y values analyzed	2001	2001	2001

Figure S30 – Luminescent lifetimes of [Eu(**PhenDMA**)]$^{3+}$ in H_2O.

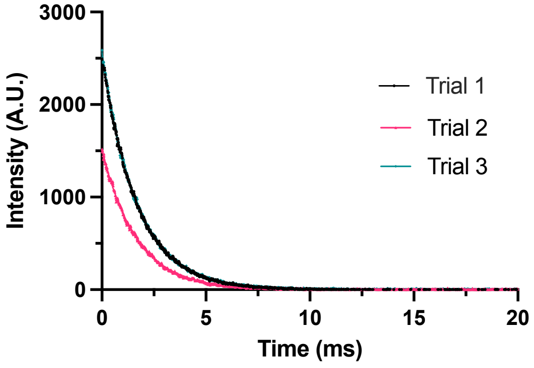

Nonlin fit<br>Table of results		Trial 1	Trial 2	Trial 3
1	One phase exponential decay			
2	Best-fit values			
3	Span	2520	1511	2558
4	K	0.5929	0.6014	0.6031
5	Plateau	-2.331	2.220	3.839
6	HalfLife	1.169	1.153	1.149
7	Tau	1.687	1.663	1.658
8	95% CI (profile likelihood)			
9	Span	2517 to 2523	1509 to 1514	2555 to 2562
10	K	0.5918 to 0.59	0.5998 to 0.60	0.6019 to 0.60
11	Plateau	-2.855 to -1.80	1.772 to 2.667	3.280 to 4.399
12	HalfLife	1.167 to 1.171	1.150 to 1.156	1.147 to 1.152
13	Tau	1.683 to 1.690	1.658 to 1.667	1.655 to 1.661
14	Goodness of Fit			
15	Degrees of Freedom	1998	1998	1998
16	R squared	0.9996	0.9991	0.9995
17	Sum of Squares	189302	139101	217130
18	Sy.x	9.734	8.344	10.42
19	Constraints			
20	K	K > 0	K > 0	K > 0
21				
22	Number of points			
23	# of X values	2001	2001	2001
24	# Y values analyzed	2001	2001	2001

Figure S31 – Luminescent lifetimes of [Eu(**PhenDMA**)]$^{3+}$ in D_2O.

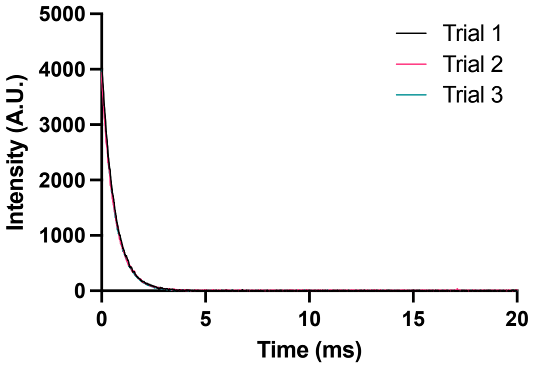

Nonlin fit<br>Table of results		A<br>Trial 1	B<br>Trial 2	C<br>Trial 3
		Y	Y	Y
1	One phase decay			
2	Best-fit values			
3	Y0	4121	3890	3996
4	Plateau	0.5483	6.316	1.450
5	K	1.619	1.613	1.647
6	Half Life	0.4282	0.4298	0.4208
7	Tau	0.6177	0.6201	0.6071
8	Span	4121	3883	3995
9	95% CI (profile likelihood)			
10	Y0	4117 to 4125	3885 to 3895	3992 to 4000
11	Plateau	0.1569 to 0.9396	5.850 to 6.783	1.083 to 1.818
12	K	1.617 to 1.621	1.610 to 1.616	1.645 to 1.650
13	Half Life	0.4275 to 0.4288	0.4291 to 0.4306	0.4202 to 0.4214
14	Tau	0.6168 to 0.6186	0.6190 to 0.6213	0.6062 to 0.6079
15	Goodness of Fit			
16	Degrees of Freedom	1998	1998	1998
17	R squared	0.9997	0.9996	0.9997
18	Sum of Squares	139503	197843	123330
19	Sy.x	8.356	9.951	7.857
20	Constraints			
21	K	K > 0	K > 0	K > 0
22				
23	Number of points			
24	# of X values	2001	2001	2001
25	# Y values analyzed	2001	2001	2001

Figure S32 – Luminescent lifetimes of [Eu(**Phenacetate**)]$^{+}$ in H_2O.

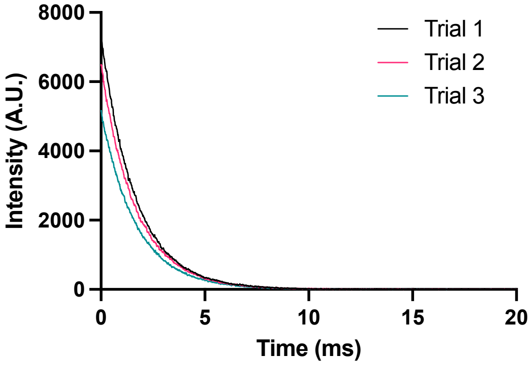

	Nonlin fit Table of results	A Trial 1	B Trial 2	C Trial 3
		Y	Y	Y
1	**One phase decay**			
2	**Best-fit values**			
3	Y0	7328	6466	5141
4	Plateau	1.456	3.257	2.208
5	K	0.6104	0.6044	0.5964
6	Half Life	1.136	1.147	1.162
7	Tau	1.638	1.655	1.677
8	Span	7327	6462	5139
9	**95% CI (profile likelihood)**			
10	Y0	7324 to 7333	6461 to 6470	5138 to 5145
11	Plateau	0.6560 to 2.256	2.446 to 4.067	1.544 to 2.873
12	K	0.6098 to 0.611(	0.6037 to 0.605	0.5957 to 0.597
13	Half Life	1.134 to 1.137	1.146 to 1.148	1.161 to 1.164
14	Tau	1.637 to 1.640	1.653 to 1.656	1.675 to 1.679
15	**Goodness of Fit**			
16	Degrees of Freedom	1998	1998	1998
17	R squared	0.9999	0.9998	0.9998
18	Sum of Squares	446959	456232	305029
19	Sy.x	14.96	15.11	12.36
20	**Constraints**			
21	K	K > 0	K > 0	K > 0
22				
23	**Number of points**			
24	# of X values	2001	2001	2001
25	# Y values analyzed	2001	2001	2001

Figure S33 – Luminescent lifetimes of [Eu(**Phenacetate**)]$^{+}$ in D_2O.

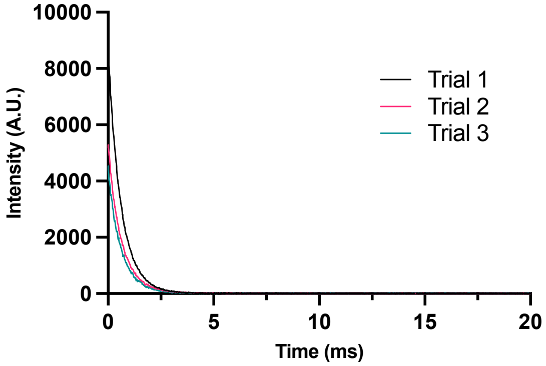

	Nonlin fit Table of results	A Trial 1	B Trial 2	C Trial 3
		Y	Y	Y
1	**One phase decay**			
2	**Best-fit values**			
3	Y0	8657	5221	4358
4	Plateau	2.036	5.405	8.118
5	K	1.633	1.584	1.619
6	Half Life	0.4245	0.4377	0.4280
7	Tau	0.6124	0.6315	0.6175
8	Span	8655	5215	4350
9	**95% CI (profile likelihood)**			
10	Y0	8650 to 8664	5213 to 5229	4351 to 4366
11	Plateau	1.364 to 2.708	4.622 to 6.188	7.397 to 8.838
12	K	1.631 to 1.635	1.580 to 1.587	1.615 to 1.624
13	Half Life	0.4240 to 0.4250	0.4367 to 0.4387	0.4269 to 0.4291
14	Tau	0.6117 to 0.6132	0.6300 to 0.6330	0.6159 to 0.6191
15	**Goodness of Fit**			
16	Degrees of Freedom	1998	1998	1998
17	R squared	0.9998	0.9993	0.9992
18	Sum of Squares	411451	555650	472135
19	Sy.x	14.35	16.68	15.37
20	**Constraints**			
21	K	K > 0	K > 0	K > 0
22				
23	**Number of points**			
24	# of X values	2001	2001	2001

Figure S34 – Luminescent lifetimes of [Eu(**Phencrypt**)]$^{3+}$ in H_2O.

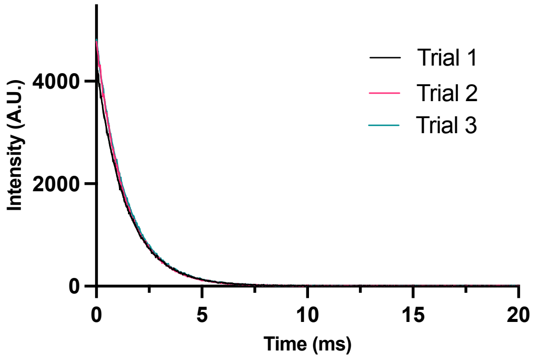

Nonlin fit Table of results	Trial 1	Trial 2	Trial 3
1 One phase decay			
2 Best-fit values			
3 Y0	4328	4799	4817
4 Plateau	2.871	4.849	3.853
5 K	0.7089	0.7469	0.7258
6 Half Life	0.9778	0.9280	0.9550
7 Tau	1.411	1.339	1.378
8 Span	4325	4794	4813
9 95% CI (profile likelihood)			
10 Y0	4324 to 4332	4795 to 4803	4813 to 4821
11 Plateau	2.272 to 3.471	4.236 to 5.462	3.268 to 4.438
12 K	0.7079 to 0.7099	0.7459 to 0.7479	0.7249 to 0.7267
13 Half Life	0.9764 to 0.9791	0.9268 to 0.9292	0.9539 to 0.9562
14 Tau	1.409 to 1.413	1.337 to 1.341	1.376 to 1.379
15 Goodness of Fit			
16 Degrees of Freedom	1998	1998	1998
17 R squared	0.9998	0.9998	0.9998
18 Sum of Squares	267852	285923	257925
19 Sy.x	11.58	11.96	11.36
20 Constraints			
21 K	K > 0	K > 0	K > 0
22			
23 Number of points			
24 # of X values	2001	2001	2001
25 # Y values analyzed	2001	2001	2001

Figure S35 – Luminescent lifetimes of [Eu(**Phencrypt**)]$^{3+}$ in D_2O.

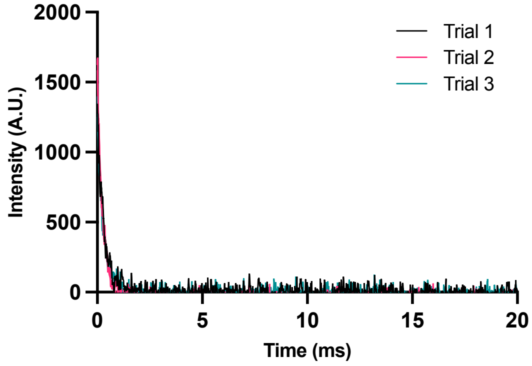

Nonlin fit Table of results	A Trial 1	B Trial 2	C Trial 3
	Y	Y	Y
1 One phase decay			
2 Best-fit values			
3 Y0	1295	1582	1248
4 Plateau	-0.7562	-51.89	-9.860
5 K	3.224	3.911	3.354
6 Half Life	0.2150	0.1772	0.2067
7 Tau	0.3102	0.2557	0.2982
8 Span	1295	1634	1258
9 95% CI (profile likelihood)			
10 Y0	1267 to 1323	1556 to 1608	1223 to 1274
11 Plateau	-2.577 to 1.064	-53.43 to -50.35	-11.49 to -8.230
12 K	3.121 to 3.330	3.817 to 4.008	3.252 to 3.460
13 Half Life	0.2082 to 0.2221	0.1730 to 0.1816	0.2004 to 0.2132
14 Tau	0.3003 to 0.3204	0.2495 to 0.2620	0.2890 to 0.3075
15 Goodness of Fit			
16 Degrees of Freedom	1998	1998	1998
17 R squared	0.8899	0.9369	0.9015
18 Sum of Squares	3221922	2326662	2586278
19 Sy.x	40.16	34.12	35.98
20 Constraints			
21 K	K > 0	K > 0	K > 0
22			
23 Number of points			
24 # of X values	2001	2001	2001
25 # Y values analyzed	2001	2001	2001

Figure S36 – Luminescence lifetimes of [Eu(**Phen18c6**)]$^{3+}$ in H_2O

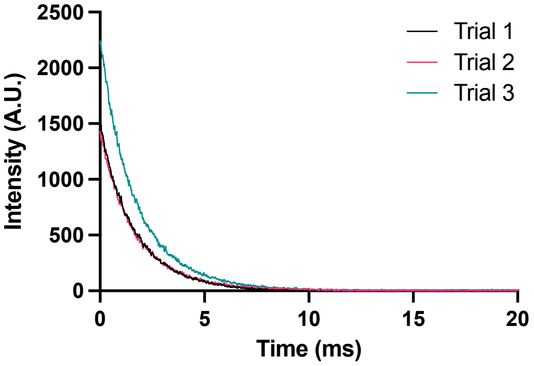

Nonlin fit Table of results	A Trial 1	B Trial 2	C Trial 3
	Y	Y	Y
1 One phase decay			
2 Best-fit values			
3 Y0	1461	1399	2207
4 Plateau	-7.685	6.012	7.676
5 K	0.5856	0.5855	0.5853
6 Half Life	1.184	1.184	1.184
7 Tau	1.708	1.708	1.709
8 Span	1469	1393	2199
9 95% CI (profile likelihood)			
10 Y0	1459 to 1464	1396 to 1402	2203 to 2211
11 Plateau	-8.183 to -7.187	5.504 to 6.520	6.964 to 8.388
12 K	0.5838 to 0.587	0.5836 to 0.587	0.5836 to 0.587
13 Half Life	1.180 to 1.187	1.180 to 1.188	1.181 to 1.188
14 Tau	1.703 to 1.713	1.703 to 1.714	1.704 to 1.714
15 Goodness of Fit			
16 Degrees of Freedom	1998	1998	1998
17 R squared	0.9989	0.9987	0.9990
18 Sum of Squares	168407	174877	343464
19 Sy.x	9.181	9.356	13.11
20 Constraints			
21 K	K > 0	K > 0	K > 0
22			
23 Number of points			
24 # of X values	2001	2001	2001
25 # Y values analyzed	2001	2001	2001

Figure S37 – Luminescent lifetimes of [Eu(**Phen18c6**)]$^{3+}$ in D_2O.

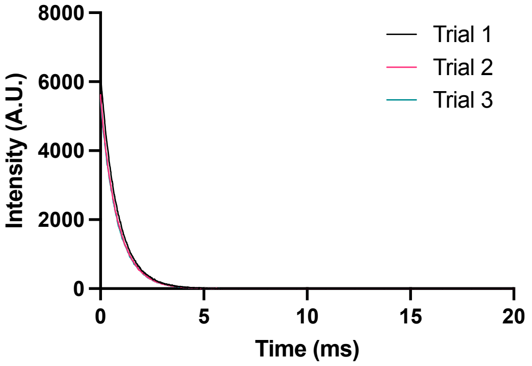

Nonlin fit Table of results	A Trial 1	B Trial 2	C Trial 3
	Y	Y	Y
1 One phase decay			
2 Best-fit values			
3 Y0	6205	5549	5429
4 Plateau	-0.2469	0.1237	0.3203
5 K	1.224	1.235	1.233
6 Half Life	0.5664	0.5615	0.5623
7 Tau	0.8172	0.8100	0.8113
8 Span	6206	5549	5429
9 95% CI (profile likelihood)			
10 Y0	6203 to 6208	5546 to 5552	5426 to 5432
11 Plateau	-0.4986 to 0.00483	-0.1929 to 0.4403	-0.003002 to 0.643
12 K	1.223 to 1.224	1.234 to 1.235	1.232 to 1.234
13 Half Life	0.5661 to 0.5667	0.5610 to 0.5619	0.5619 to 0.5628
14 Tau	0.8167 to 0.8176	0.8094 to 0.8107	0.8106 to 0.8119
15 Goodness of Fit			
16 Degrees of Freedom	1998	1998	1998
17 R squared	1.000	0.9999	0.9999
18 Sum of Squares	55089	87288	90999
19 Sy.x	5.251	6.610	6.749
20 Constraints			
21 K	K > 0	K > 0	K > 0
22			
23 Number of points			
24 # of X values	2001	2001	2001
25 # Y values analyzed	2001	2001	2001

Figure S38 – Luminescent lifetimes of [Eu(**BipyDMA**)]$^{3+}$ in H_2O.

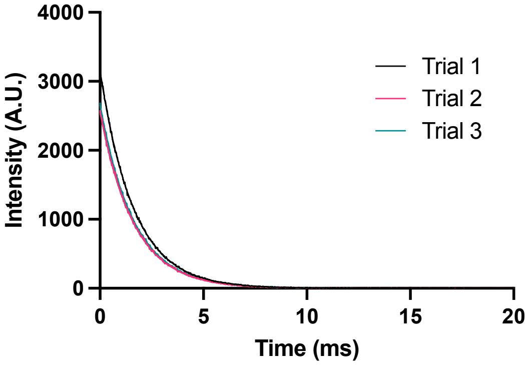

	Nonlin fit Table of results	A Trial 1	B Trial 2	C Trial 3
		Y	Y	Y
1	**One phase decay**			
2	**Best-fit values**			
3	Y0	3155	2559	2660
4	Plateau	-0.6788	-0.4676	-0.1567
5	K	0.6162	0.6130	0.6126
6	Half Life	1.125	1.131	1.131
7	Tau	1.623	1.631	1.632
8	Span	3156	2559	2660
9	**95% CI (profile likelihood)**			
10	Y0	3153 to 3157	2557 to 2561	2658 to 2662
11	Plateau	-1.051 to -0.307	-0.8035 to -0.13	-0.5015 to 0.188
12	K	0.6156 to 0.616	0.6123 to 0.613	0.6119 to 0.613
13	Half Life	1.124 to 1.126	1.129 to 1.132	1.130 to 1.133
14	Tau	1.621 to 1.624	1.629 to 1.633	1.630 to 1.634
15	**Goodness of Fit**			
16	Degrees of Freedom	1998	1998	1998
17	R squared	0.9999	0.9998	0.9998
18	Sum of Squares	97036	79023	83212
19	Sy.x	6.969	6.289	6.454
20	**Constraints**			
21	K	K > 0	K > 0	K > 0
22				
23	**Number of points**			
24	# of X values	2001	2001	2001
25	# Y values analyzed	2001	2001	2001

Figure S39 – Luminescent lifetimes of [Eu(**BipyDMA**)]$^{3+}$ in D_2O.

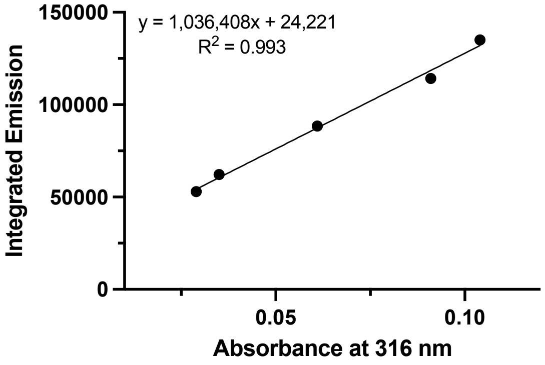

Figure S40 – Integrated emission vs absorbance curves for quinine sulfate in 0.5 M H_2SO_4.

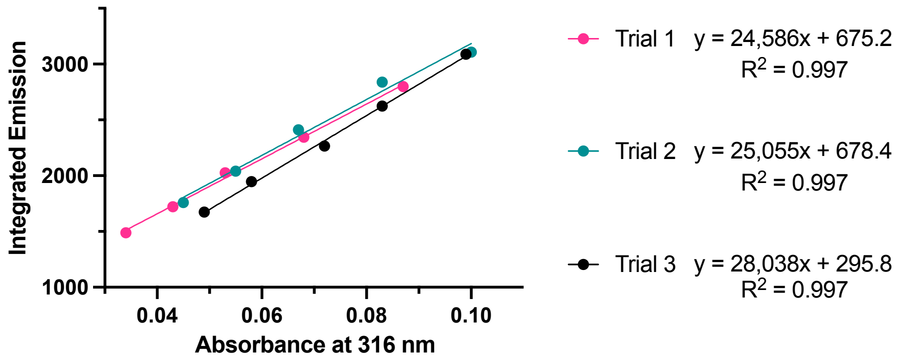

Figure S41 – Integrated emission vs absorbance curves for Eu(**BipyDMA**)$^{3+}$ in tris buffer (10 mM, pH 7.4) used in quantum yield measurement against quinine sulfate. Instrumental parameters were the following: 5.0 nm excitation slit width, a 2.5 nm emission slit width and a PMT voltage of 400 V.

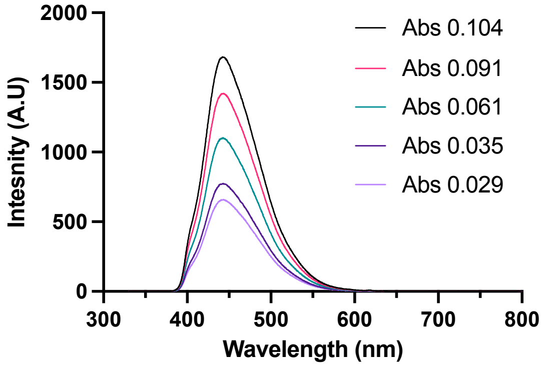

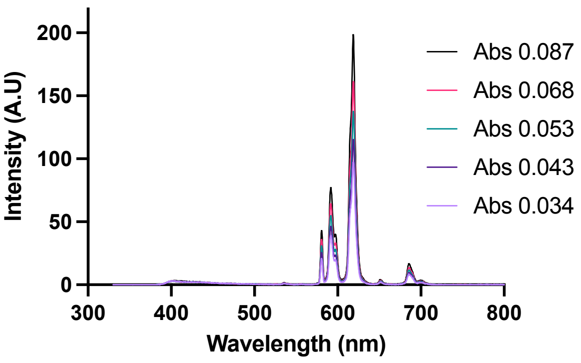

Figure S42 – Plot of emission spectra for the quantum yield measurement of quinine sulfate (left) and trial 1 of Eu(**BipyDMA**)$^{3+}$ (right) showing the distinct emission profiles between the standard and compound of interest.

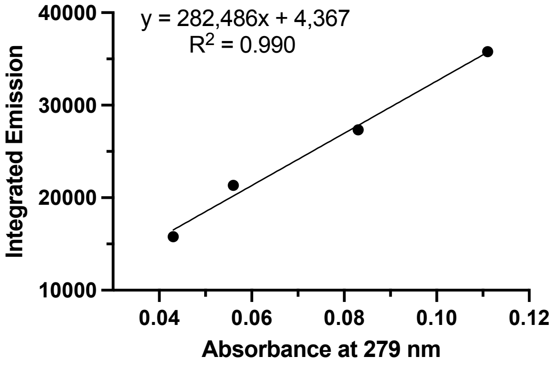

Figure S43 - Integrated emission vs absorbance curves Eu(DPA)$_3$ in tris buffer (100 mM, pH 7.4).

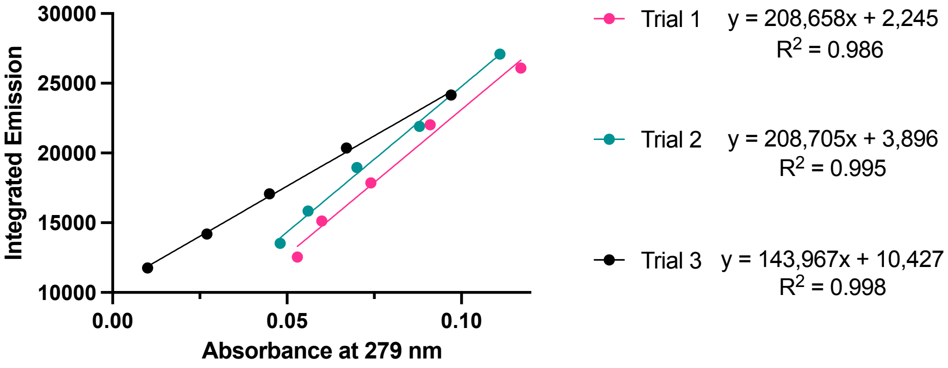

Figure S44 – Integrated emission vs absorbance curves for Eu(**BipyDMA**)$^{3+}$ in tris buffer (10 mM, pH 7.4) used in quantum yield measurement against Eu(DPA)$_3$. Instrumental parameters were the following: 2.5 nm excitation and emission slit widths and a PMT voltage of 700 V.

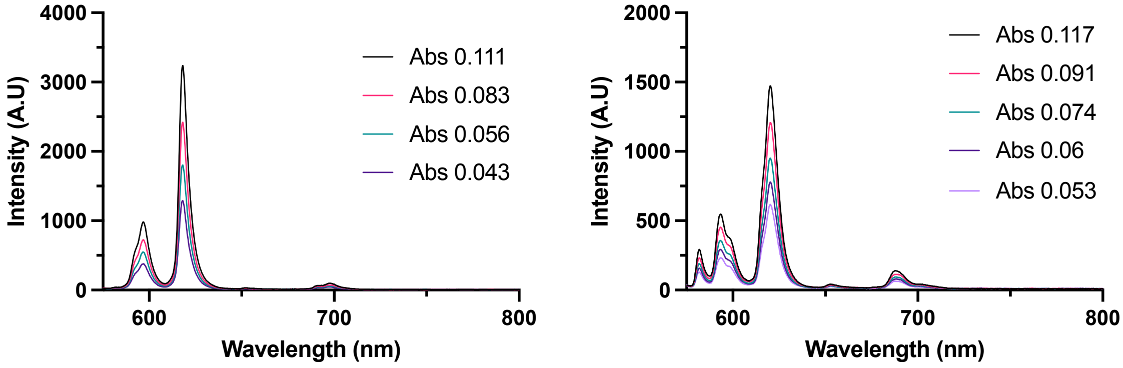

Figure S45 – Emission spectra collected for the quantum measurement of Eu(DPA)$_3$ (left) and trial 1 of Eu(**BipyDMA**)$^{3+}$ (right).

4. Electron Paramagnetic Resonance

EPR spectra of Eu(**phen18c6**) were collected between 110 and 220 K with the following experimental parameters: frequency = 9.386 GHz; microwave power = 10.02 mW; modulation amplitude = 3.0 G; modulation frequency = 100 kHz; time constant = 81.92 ms; conversion time = 40.96 ms; and receiver gain = 20 dB.

The spectra at 15 K were acquired using the following experimental parameters: frequency = 9.386 GHz; microwave power = 12.62 mW; modulation amplitude = 4.0 G; modulation frequency = 100 kHz; time constant = 20.48 ms; conversion time = 20.48 ms; and receiver gains for gadolinium and europium were set to 40 and 60 dB, respectively.

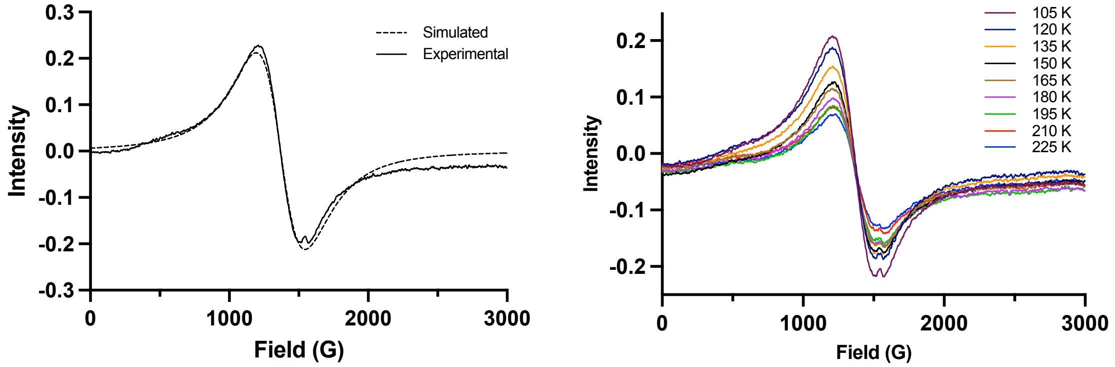

Figure S46 – X-band EPR spectra of [Eu(**Phen18c6**)]$^{2+}$ (1.0 mM) in methanol (left) collected at 105 K and (right) increasing every 15 K until 225 K.

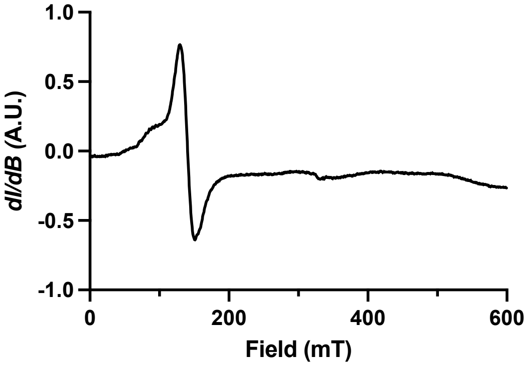

Figure S47 – X-Band EPR spectrum at 15 K of [Eu(**BipyDMA**)]$^{2+}$ (1.0 mM) in MOPS buffer (10 mM, pH 7.4).

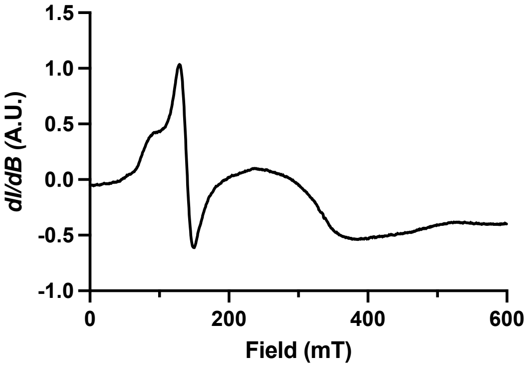

Figure S48 – X-Band EPR spectrum at 15 K of [Eu(**PhenDMA**)]$^{2+}$ (1.0 mM) in MOPS buffer (10 mM, pH 7.4).

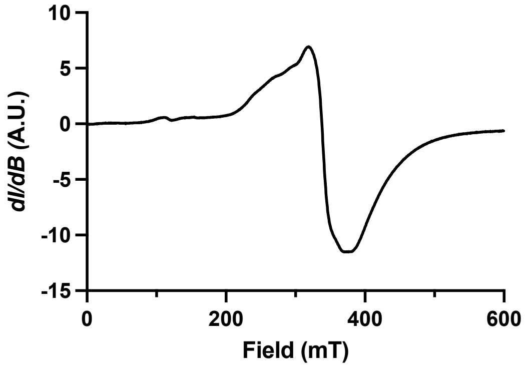

Figure S49 – X-Band EPR spectrum at 15 K of [Gd(**PhenDMA**)]$^{3+}$ (1.0 mM) in MOPS buffer (10 mM, pH 7.4)

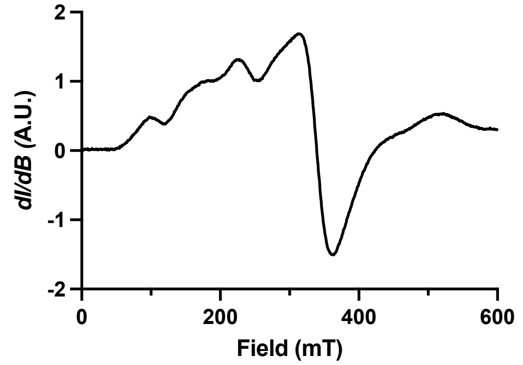

Figure S50 – X-Band EPR spectrum at 15 K of [Gd(**PhenDMA**)]$^{3+}$ (1.0 mM) in MOPS buffer (10 mM, pH 7.4).

5. Cyclic Voltammetry

Table S1 – Summary of Electrochemical Data

	$\mathbf{E_{pa}}$ (V vs Fc/Fc$^+$)	$\mathbf{E_{pc}}$ (V vs Fc/Fc$^+$)	**ΔE** (V)
[Eu(**BipyDMA**)]$^{2+/3+}$	–0.86	–1.31	0.45
[Eu(**Phen18c6**)]$^{2+/3+}$	–1.04	–1.24	0.20
[Eu(**PhenDMA**)]$^{2+/3+}$	–0.87	–1.43	0.56
[Eu(**Phencrypt**)]$^{2+/3+}$	–0.83	–1.31	0.48

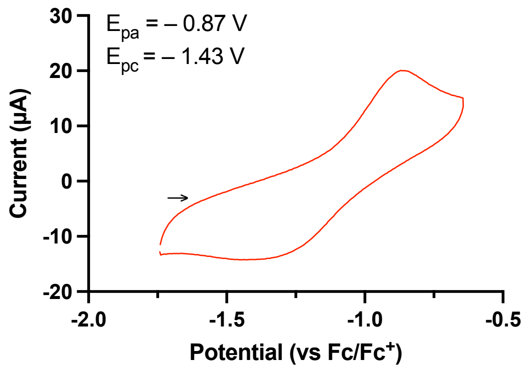

Figure S51 – Cyclic voltammogram of Eu(**PhenDMA**)]$^{2+}$ with LiCl (100 mM) in methanol.

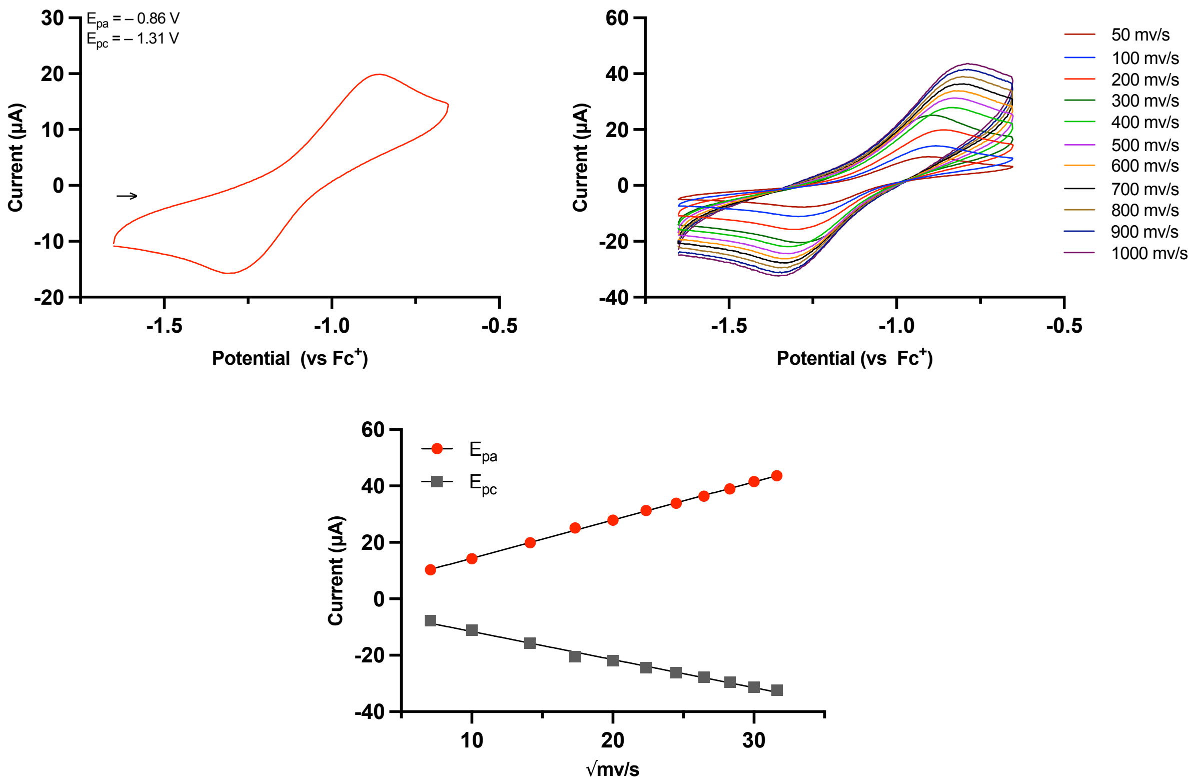

Figure S52 – Cyclic voltammograms of [Eu(**BipyDMA**)]$^{2+}$ with LiCl (100 mM) in methanol.

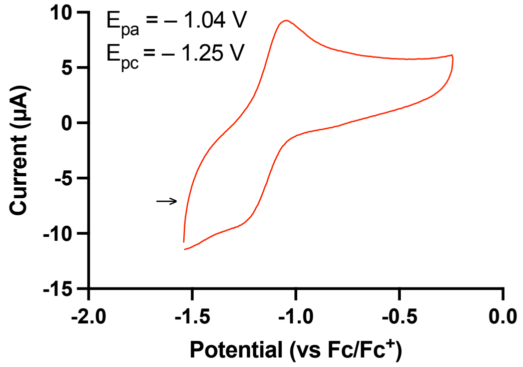

Figure S53 – Cyclic voltammogram of [Eu(**Phen18c6**)]$^{2+}$ with LiCl (100 mM) in methanol.

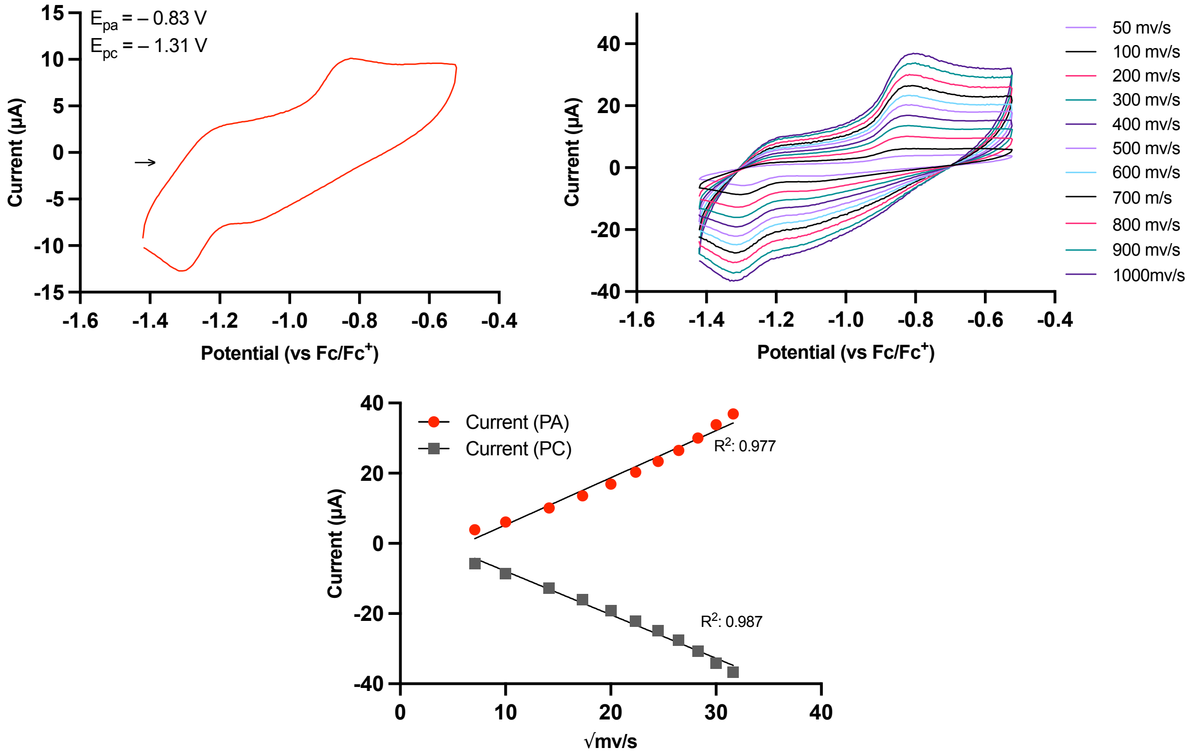

Figure S54 – Cyclic voltammograms of [Eu(**Phencrypt**)]$^{2+}$ with LiCl (100 mM) in methanol.

6. Relaxivity Measurements

Divalent europium compounds were prepared in a glovebox under an atmosphere of N_2 (<0.5 ppm O_2). A ligand solution (17.53–77.3 mM) in MOPS buffer (10 mM, pH 7.4) was mixed in a 1:1 molar ratio with a solution of $EuBr_2$ (13.5 mM) in water, and the reaction was stirred for 15 min. The resulting solution was diluted to 1,000 μL at a final concentration of 1.0 mM in MOPS buffer (10 mM, pH 7.4). That solution was diluted in NMR tubes to concentrations of 0.50, 0.25, and 0.125 mM at total volumes of 471 μL. NMR tubes were flame sealed. T_1 measurements were obtained on a Bruker Minispec mq60 NMR (1.4 T) spectrometer by measuring T_1 with inversion-recovery experiments at 37 °C. Longitudinal relaxivities were determined by the slope of $1/T_1$ versus concentration using linear regression fitting.

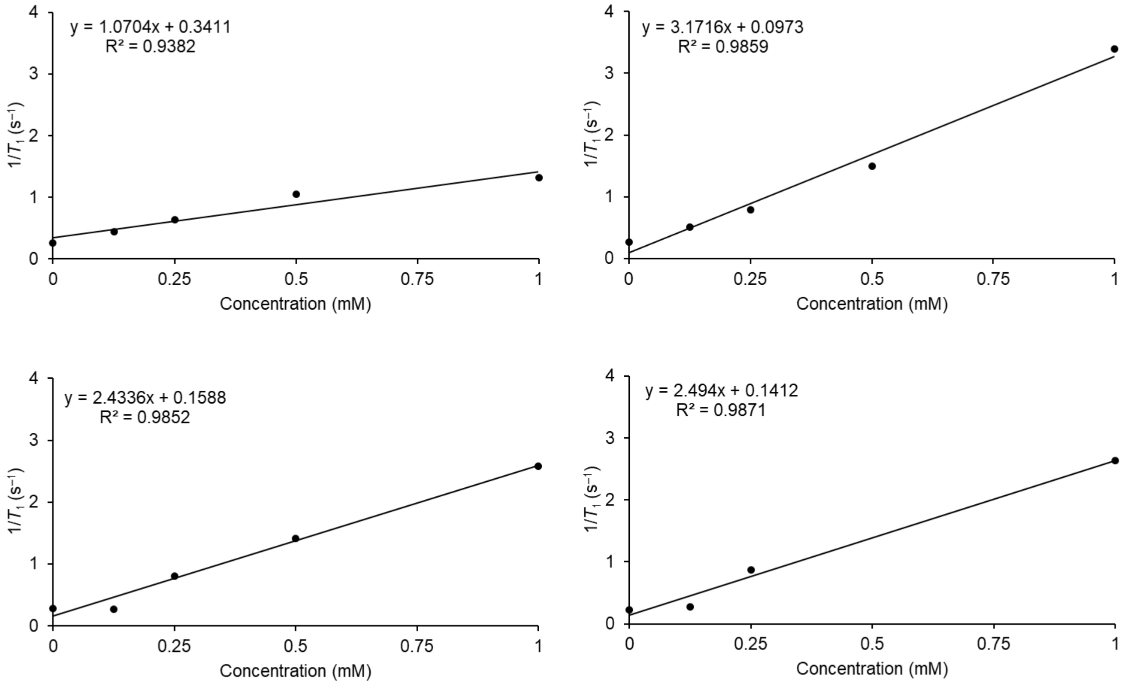

Figure S55 –Longitudinal relaxation rate ($1/T_1$) versus concentration of [Gd(**BipyDMA**)]$^{3+}$ (top left), [Gd(**PhenDMA**)]$^{3+}$ (top right), [Eu(**BipyDMA**)]$^{2+}$ (bottom left), and [Eu(**PhenDMA**)]$^{2+}$(bottom right).

7. T_1-weighted in vitro images

Divalent europium samples were prepared in a glovebox. A solution of ligand (1.0 mM) in MOPS buffer (10 mM, pH 7.4) was mixed in a 1:1 molar ratio with a solution of $EuBr_2$ (14.15 mM) in water, and the reaction was stirred for 15 min. From the resulting solution, individual samples were prepared by dilution to 0.25, 0.125, and 0.05 mM in MOPS buffer (10 mM, pH 7.4) to a total volume of 200 µL in 600 µL Eppendorf tubes. The tubes were closed and wrapped with parafilm before being removed from the glovebox for analysis. Images were acquired on a 4.7 T small-animal MRI scanner.

Region of interest (ROI) analysis was performed using the ImageJ software and the ΔSNR was calculated using following equations:[9,10]

$$SNR = \frac{SI_{mean}}{SD_{noise}} * 0.66$$

$$\Delta SNR = \frac{SNR_{sample} - SNR_{water}}{SNR_{water}} * 100\%$$

Where SNR is the signal-to-noise ratio, the standard deviation (SD) of the noise being determined by the corners of the image, SI is the signal intensity, and 0.66 is the Rayleigh correction factor.

Table S2 – Tabulated ΔSNR for T_1-weighted in vitro image experiments.

	0.25 mM	0.125 mM	0.05 mM
[Eu(**BipyDMA**)]$^{2+}$	200.6	115.4	–7.3
[Eu(**BipyDMA**)]$^{2+}$ + O_2	–0.1	10.0	–14.5
[Eu(**PhenDMA**)]$^{2+}$	115.8	–2.3	–10.2
[Eu(**PhenDMA**)]$^{2+}$ + O_2	–1.4	8.6	0.8
[Gd(**BipyDMA**)]$^{3+}$	266.0	168.5	84.6
[Gd(**PhenDMA**)]$^{3+}$	262.8	118.3	33.5

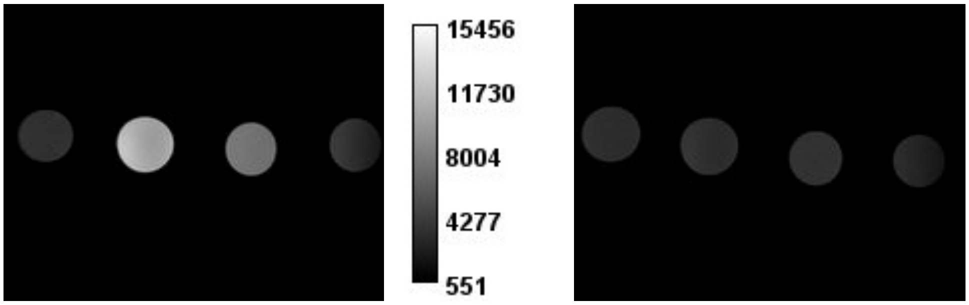

Figure S56 – T_1-weighted image of [Eu(**BipyDMA**)]$^{2+}$ (left) and the same samples after exposure to air (right). Sample concentrations from left to right: 0.25, 0.125, and 0.05 mM.

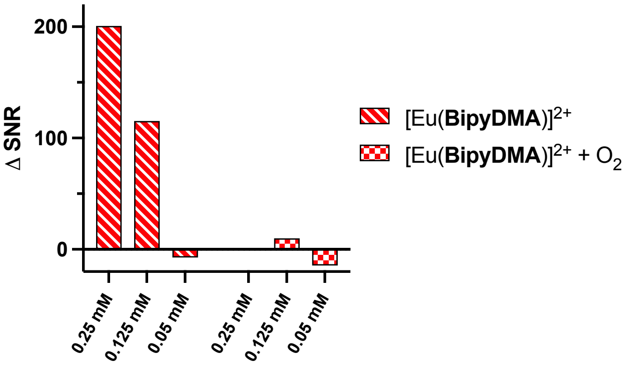

Figure S57 – ROI analysis of T_1-weighted images of [Eu(**BipyDMA)**]$^{2+}$.

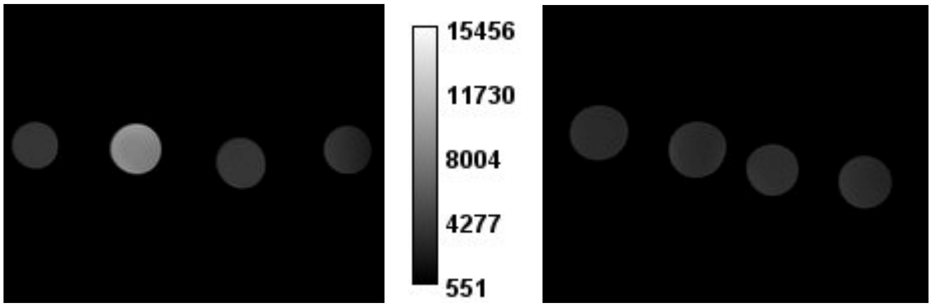

Figure S58 - T_1-weighted images of [Eu(**PhenDMA**)]$^{2+}$ (left) and the same samples after exposure to air (right). Sample concentrations from left to right: 0.25, 0.125, and 0.05 mM.

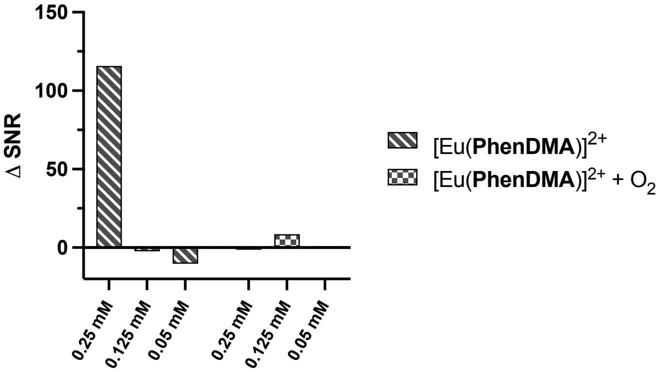

Figure S59 - ROI analysis of T_1-weighted images of [Eu(**PhenDMA**)]$^{2+}$ before and after exposure to air.

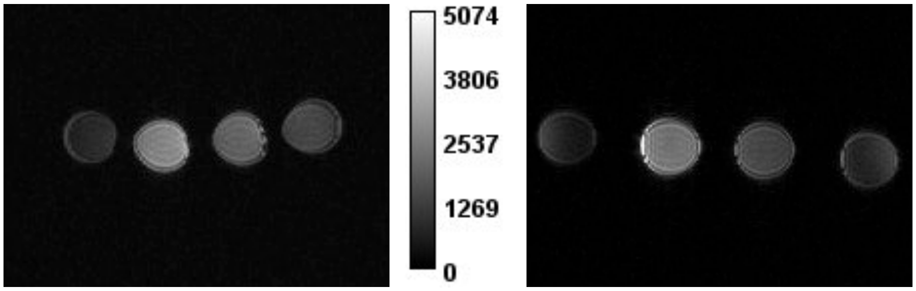

Figure S60 – T_1-weighted images of [Gd(**bipyDMA**)]$^{3+}$ (left) and [Gd(**phenDMA**)]$^{3+}$ (right). Sample concentrations from left to right: solvent blank, 0.25, 0.125, and 0.05 mM.

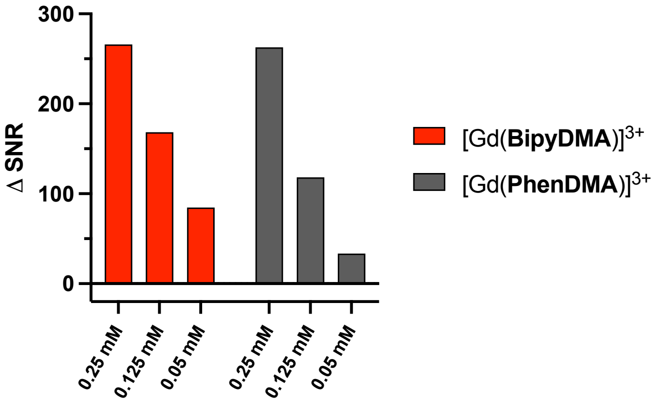

Figure S61 – ROI analysis of T_1-weighted images of [Gd(**PhenDMA**)]$^{3+}$ and [Gd(**BipyDMA**)]$^{3+}$.

8. CRET in vitro Images

The initial complex concentration was determined using the respective molar absorptivity. Then, a dilution series was prepared in 600 µL Eppendorf tubes containing 50, 25, 10, 5, 1, 0.5, 0.25, and 0.1 nmol in a total volume of 190 µL.

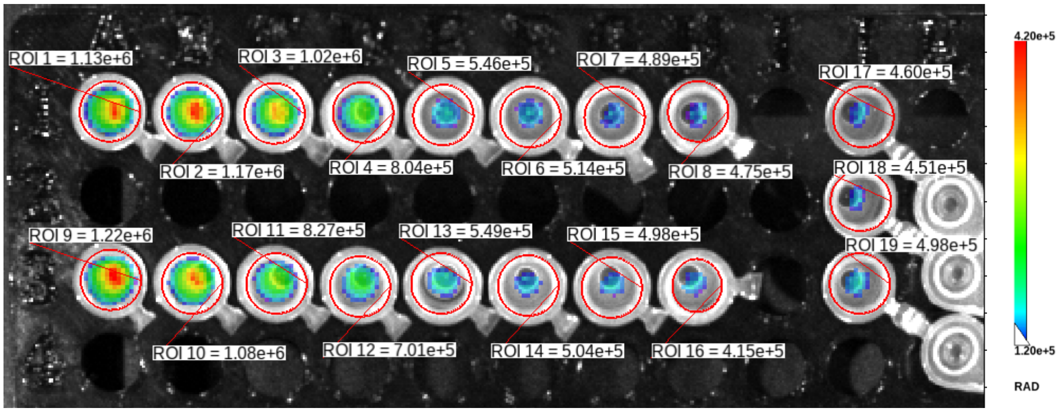

Figure S62 – CRET Plate containing ROI analysis of [Eu(**PhenDMA**)Br]Br_2 (top) and [Eu(**BipyDMA**)Br]Br_2 (bottom). The well diameter is 8 mm. Complex amounts from left to right: 50, 25, 10, 5, 1, 0.5, 0.25, 0.1 nmol. All wells were doped with ^{68}Ga (11 µCi, 10 mL) immediately before being imaged. The three tubes on the right are filled with MOPS buffer (10 mM, pH 7.4) and ^{68}Ga (10 mL, 11 µCi).

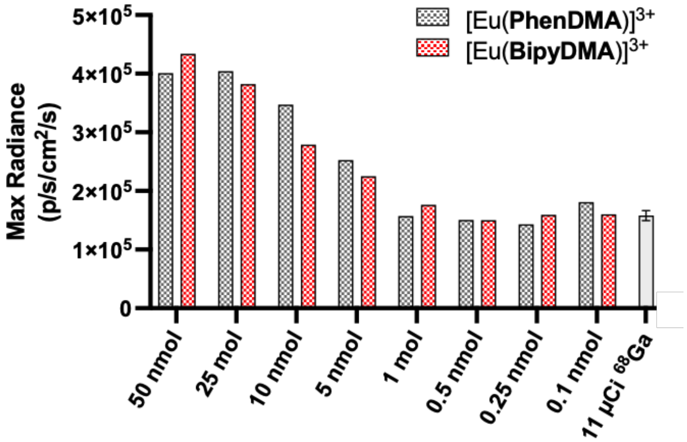

Figure S63 – ROI analysis of CRET plate performed with trivalent europium complexes.

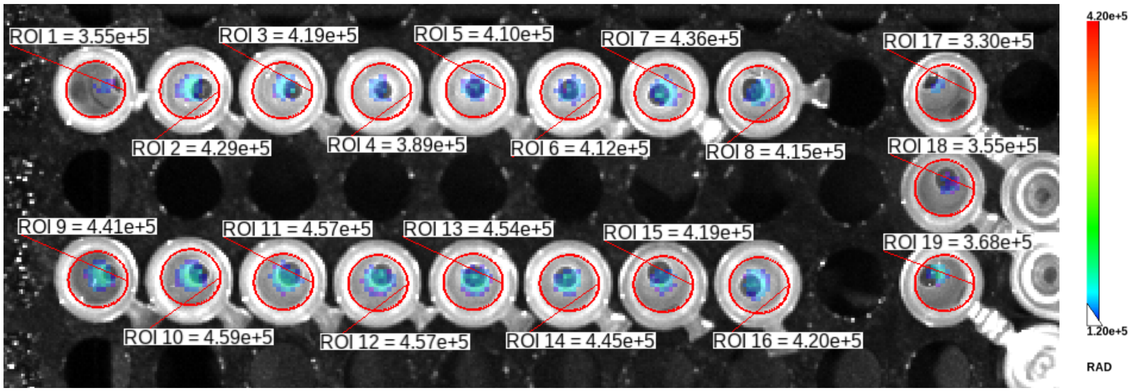

Figure S64 – CRET Plate containing ROI analysis of Gd(**PhenDMA**)Cl_3 (top) and Gd(**BipyDMA**)Cl_3 (bottom). The well diameter is 8 mm. Complex amounts from left to right: 50, 25, 10, 5, 1, 0.5, 0.25, 0.1 nmol. All wells were doped with ^{68}Ga (10 mL, 11 μCi) immediately before being imaged. The three tubes on the right are filled with MOPS buffer (10 mM, pH 7.4) and ^{68}Ga (10 mL, 10 μCi).

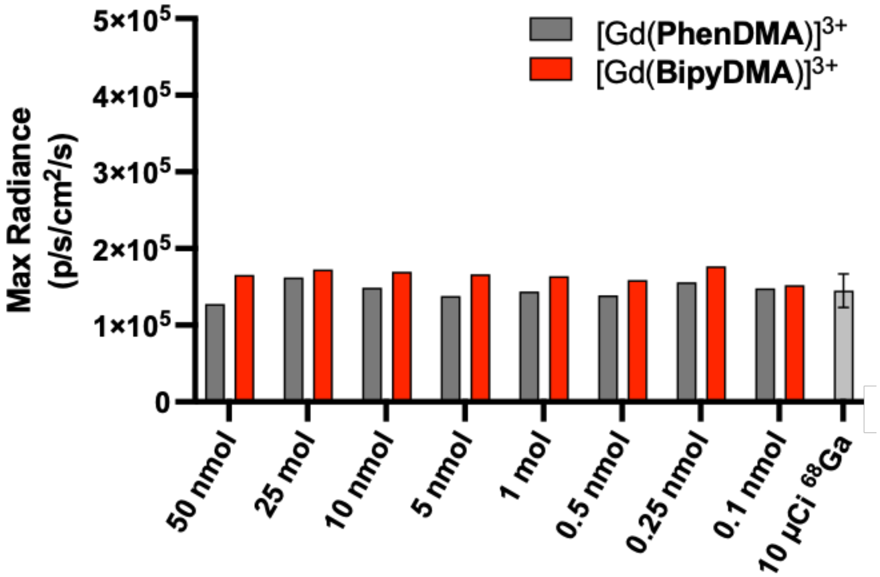

Figure S65 – ROI analysis of CRET plate performed with trivalent gadolinium complexes.

9. Bimodal *in vivo* Imaging

In an inert atmosphere glovebox, Eu(**BipyDMA**)$^{2+}$ was prepared by mixing a solution of **BipyDMA** (12.8 mM, 78 µL) with a solution of $EuBr_2$ (16.02 mM, 62 µL) and diluting to a total volume of 600 µL in MOPS buffer (10 mM, pH 7.4). The solution was loaded into a syringe (100 µL) and sealed in a flask before being removed from the glovebox.

Male 6 J mice were implanted with RM-1-PGLS cell lines (0.1 M)[11] 10 days prior to experimentation. The mouse was anesthetized using (1.5–2% v/v isoflurane in oxygen) and immobilized before being imaged prior to injection. The mouse was removed from the magnet but remained in a stable position for the intratumoral injection (100 µL). The mouse was immediately returned to the magnet and imaged for the first time point (4 min). Subsequent images were acquired until the signal returned to baseline (16 min). Upon dissipation of signal, a solution of ^{68}Ga[$GaCl_3$] (40 µL, 52 µCi) in MOPS buffer (10 mM, pH 7.4) was prepared and injected intratumorally 90 min post-injection of the Eu(**BipyDMA**)$^{2+}$. A second tumor-bearing mouse was simultaneously injected with the ^{68}Ga[$GaCl_3$] solution (40 mL, 52 µCi) as a control. The mice were imaged after 5 min on an IVIS Lumina series III using a 620 nm filter and a 5 min acquisition time.

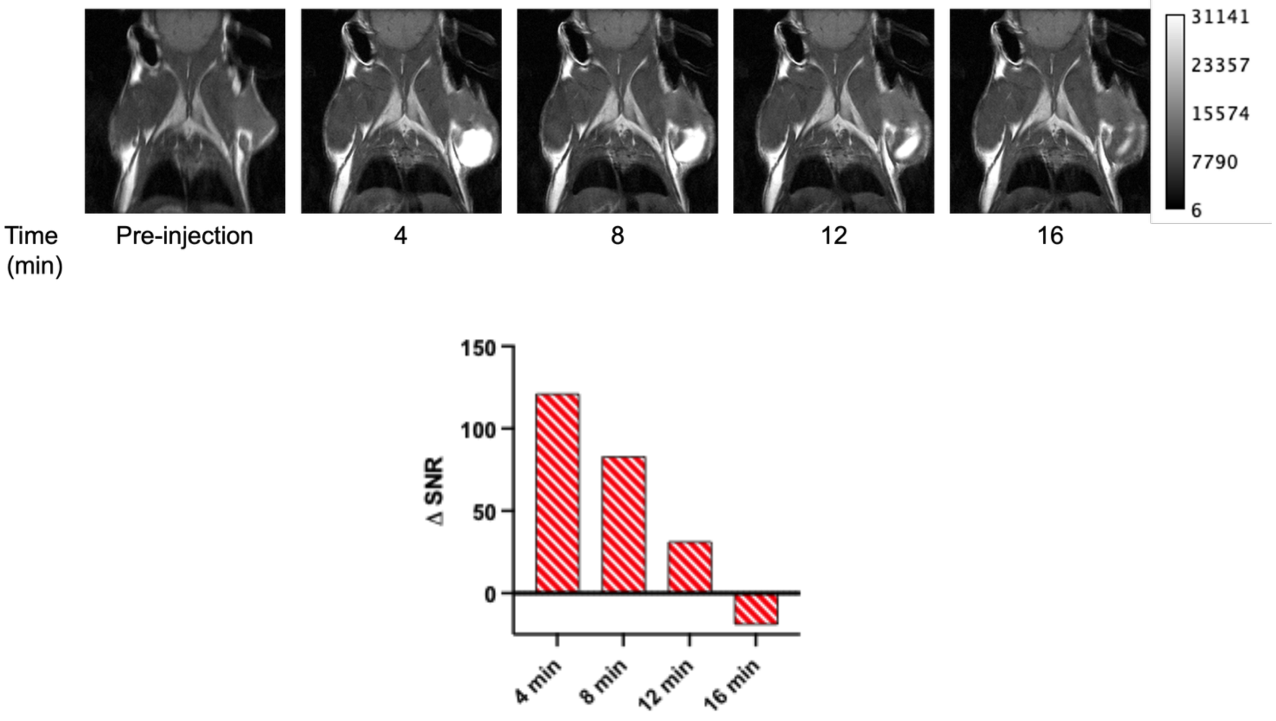

Figure S66 – Time progression of T_1-weighted contrast as Eu^{2+} oxidizes.

a) 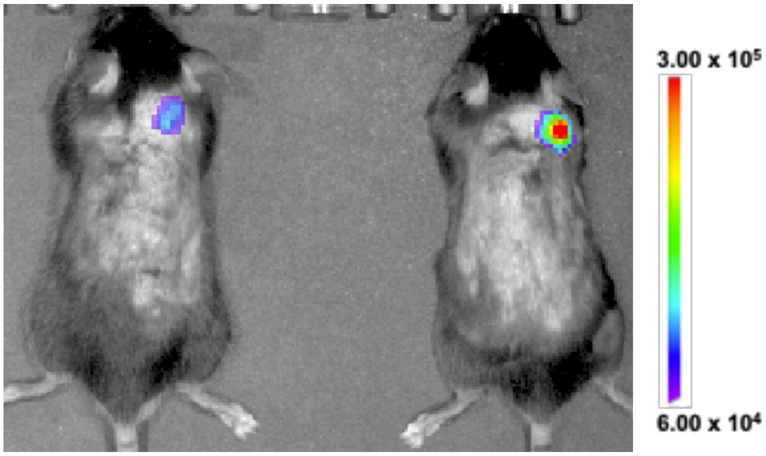

b) 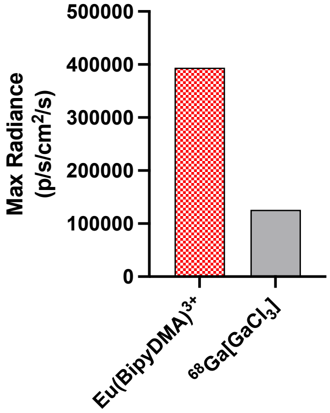

Figure S67 – a) CRET luminescence of a control tumor-bearing mouse administered with ^{68}Ga (52 mCi) (left). CRET luminescence of the tumor-bearing mouse that was administered Eu(**BipyDMA**)$^{2+}$ along with ^{68}Ga (52 mCi) (right). b) ROI analysis of the CRET image. Tumor sizes were 6.37 x 7.16 mm (left) and 8.11 x 6.92 mm (right).

10. NMR Spectra

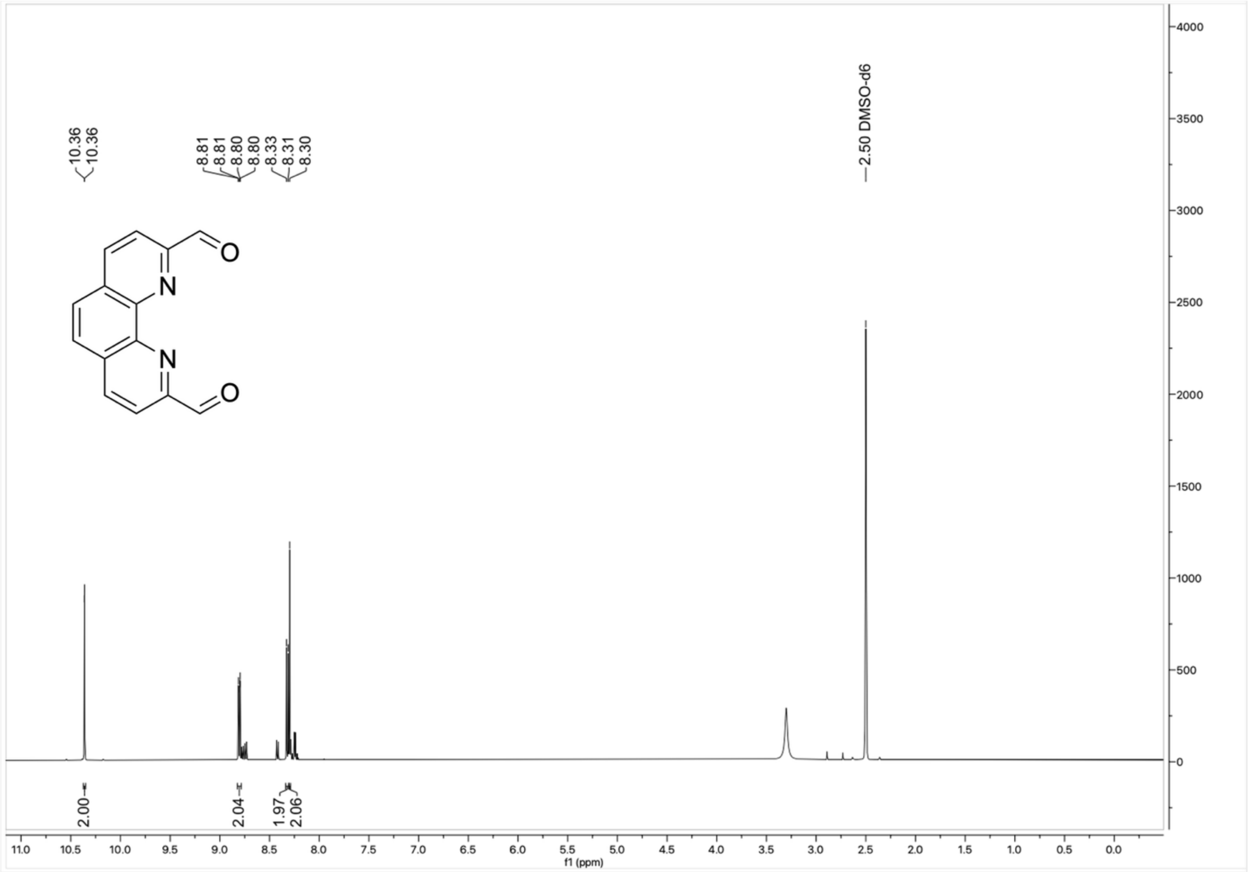

Figure S68 – ^{1}H-NMR spectrum of **Phenanthroline Dialdehyde (1)** in DMSO-d_6.

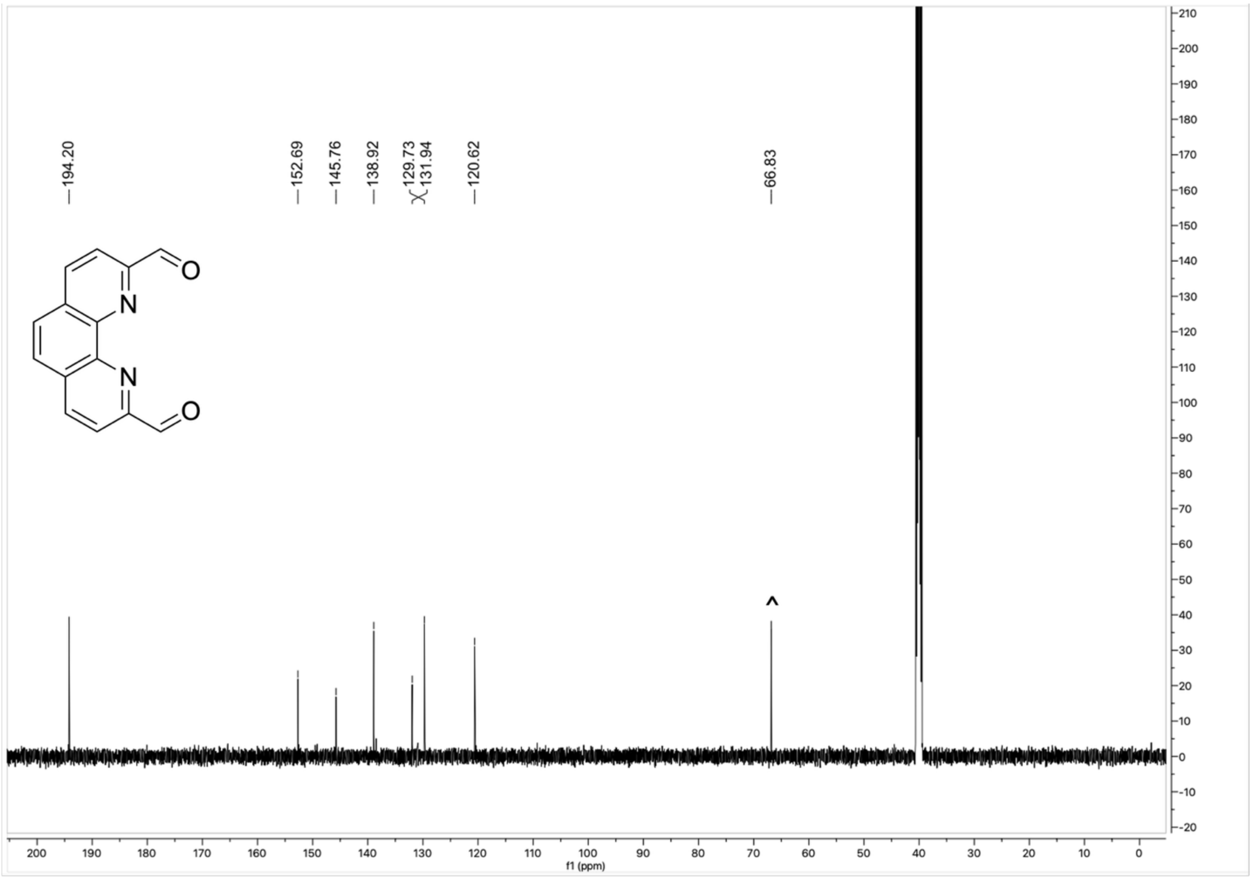

Figure S69 – ^{13}C-NMR spectrum of **Phenanthroline Dialdehyde (1)** in DMSO-d_6. (^ = dioxane)

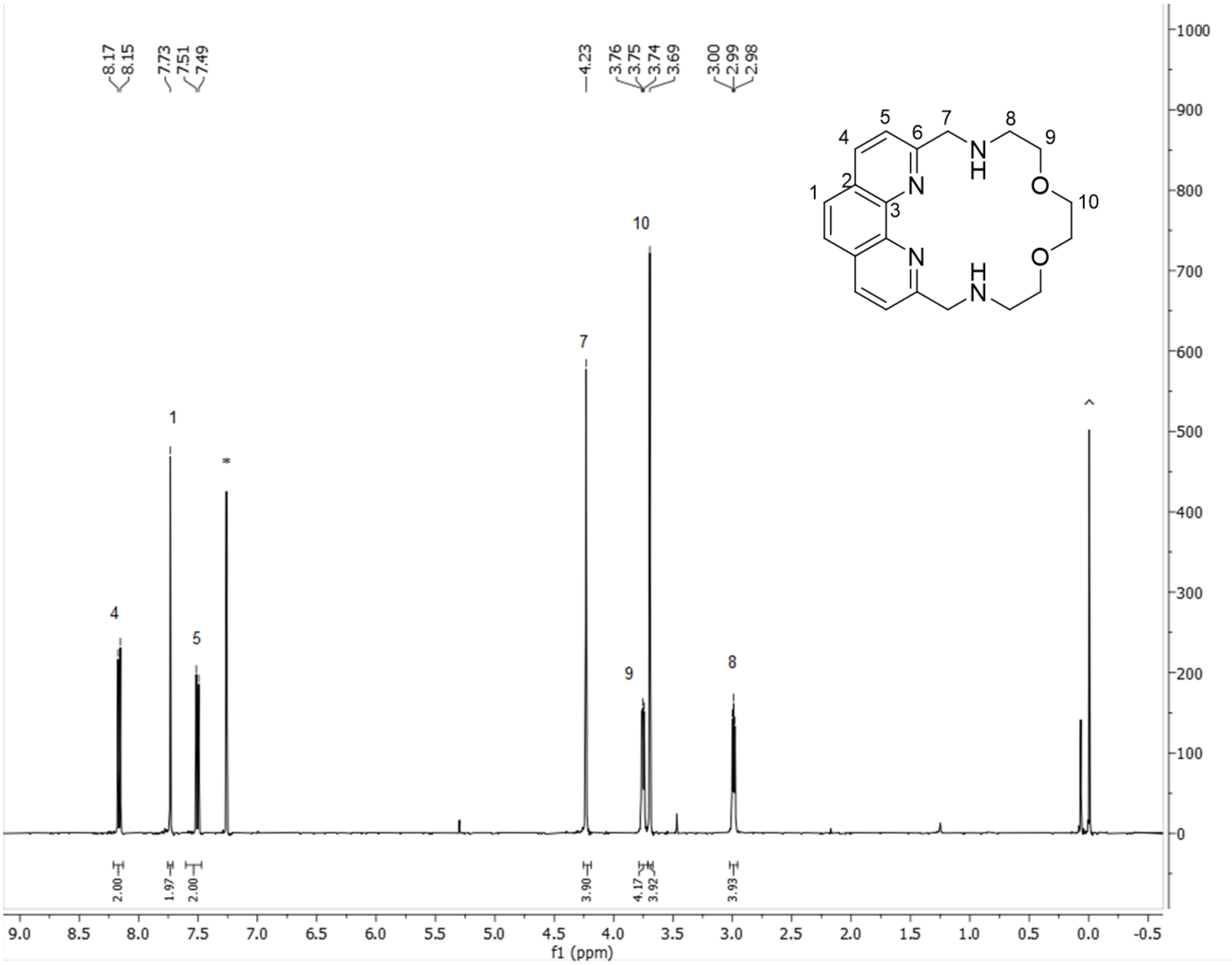

Figure S70 – ^{1}H-NMR spectrum of **phen18c6** in $CDCl_3$ (*) with TMS (^).

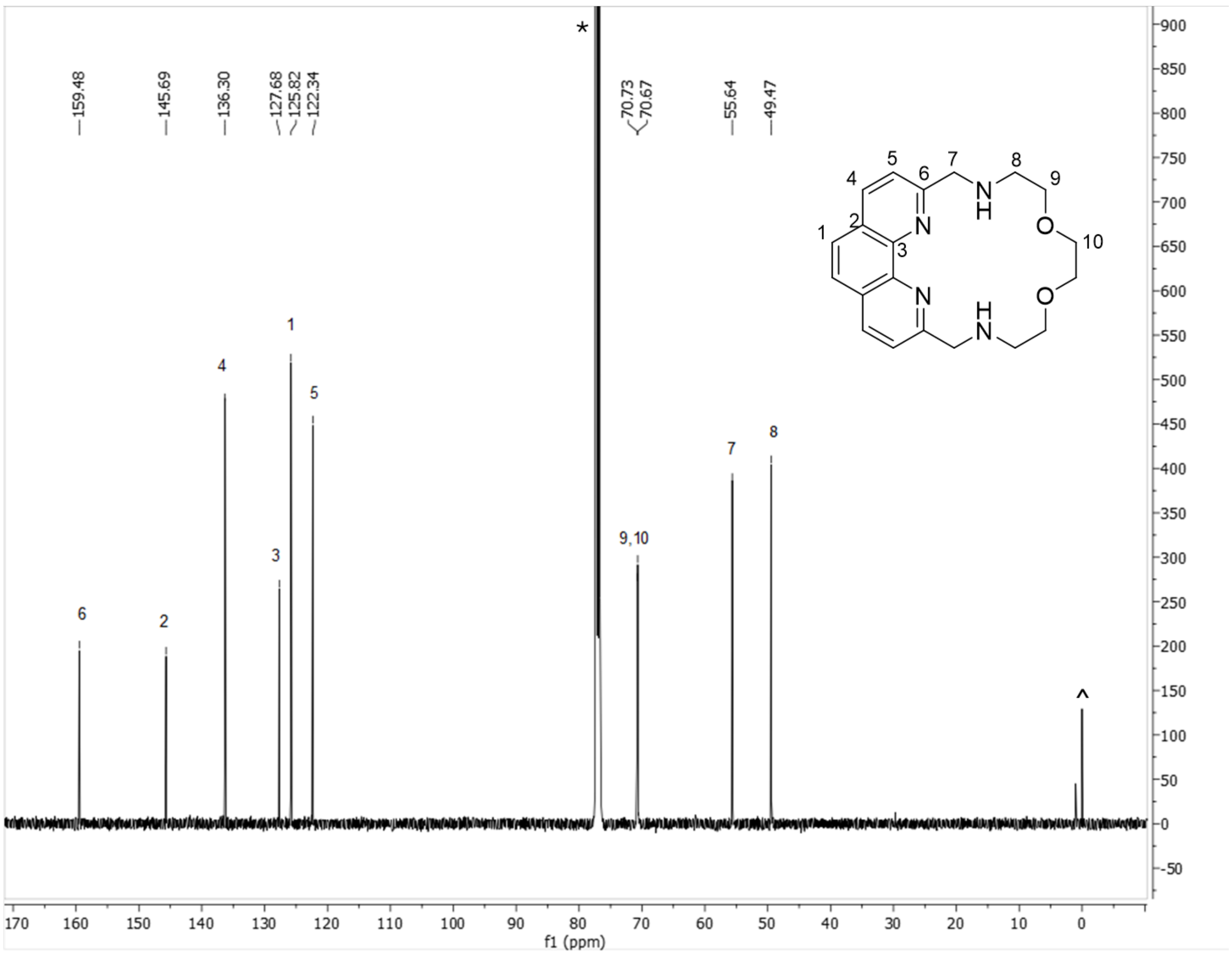

Figure S71 – ^{13}C-NMR spectrum of **phen18c6** in $CDCl_3$ (*) with TMS (^).

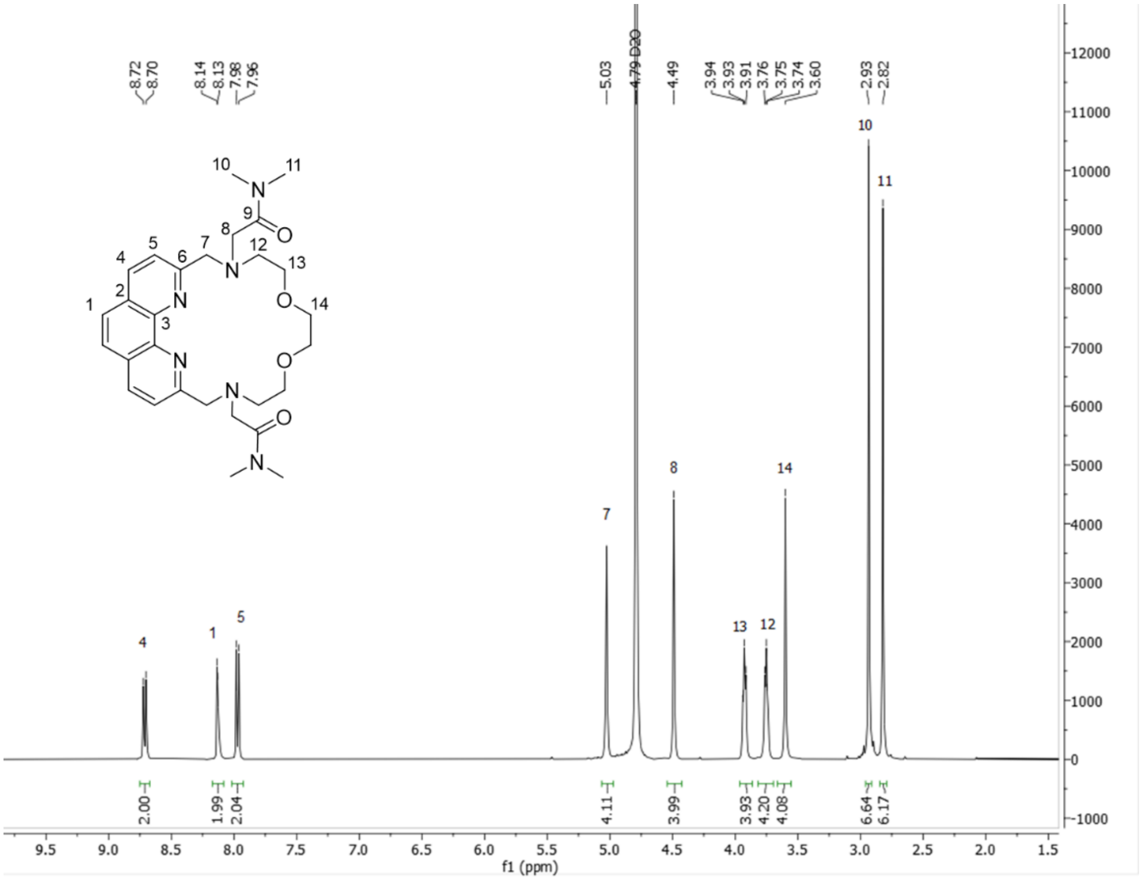

Figure S72 – ^{1}H-NMR spectrum of **PhenDMA** in D_2O.

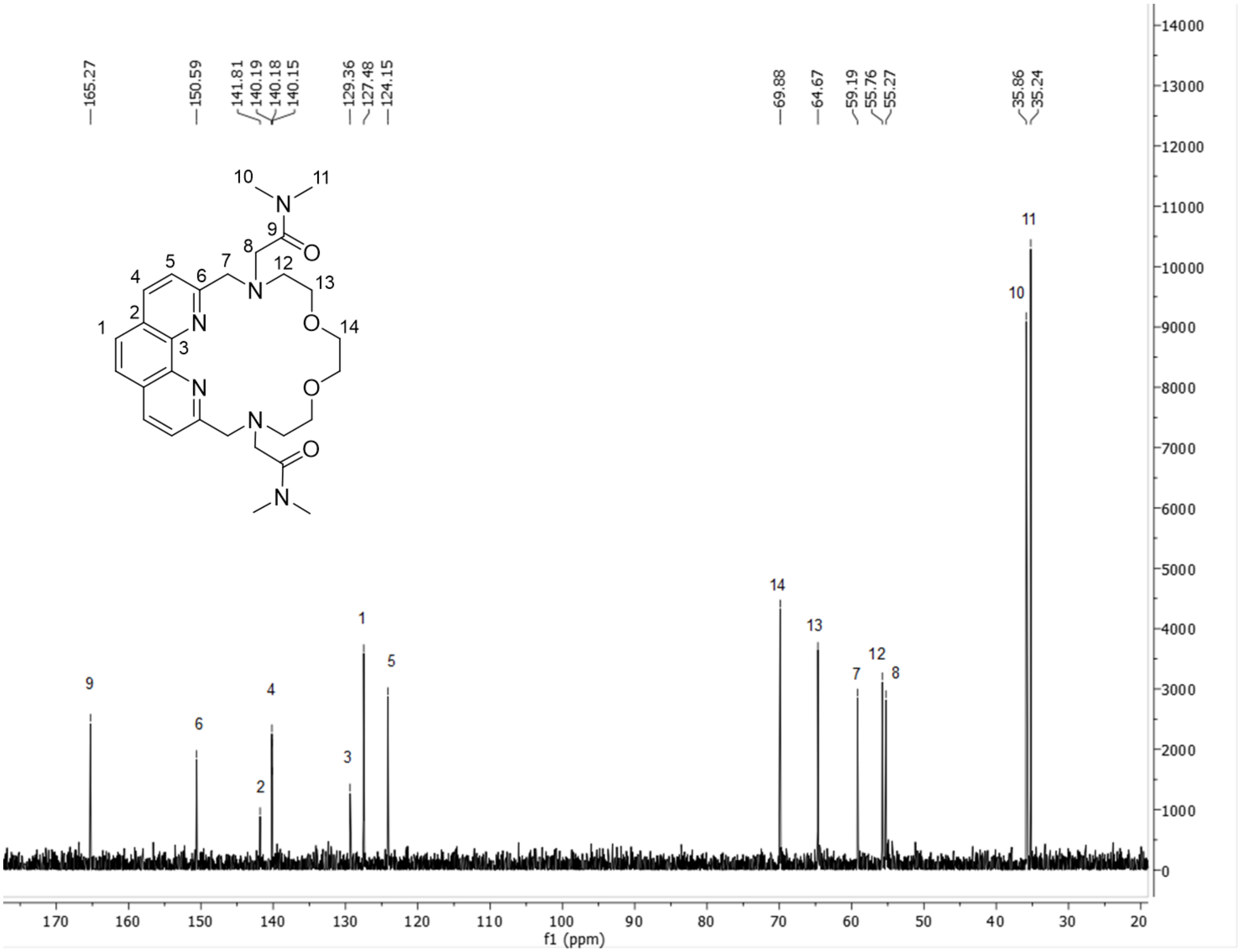

Figure S73 – ^{13}C-NMR spectrum of **PhenDMA** in D_2O.

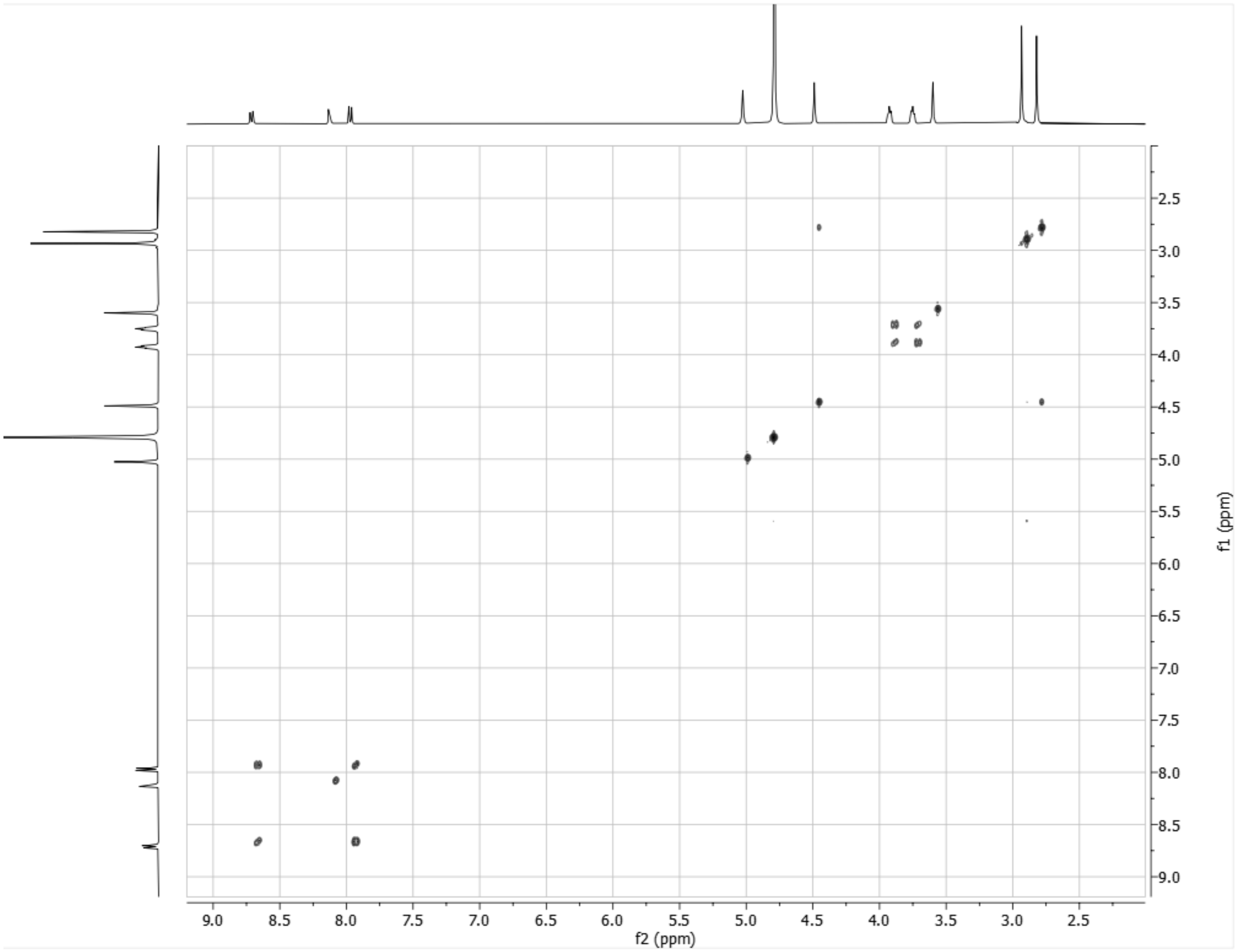

Figure S74 – ^{1}H-^{1}H COSY of **PhenDMA**

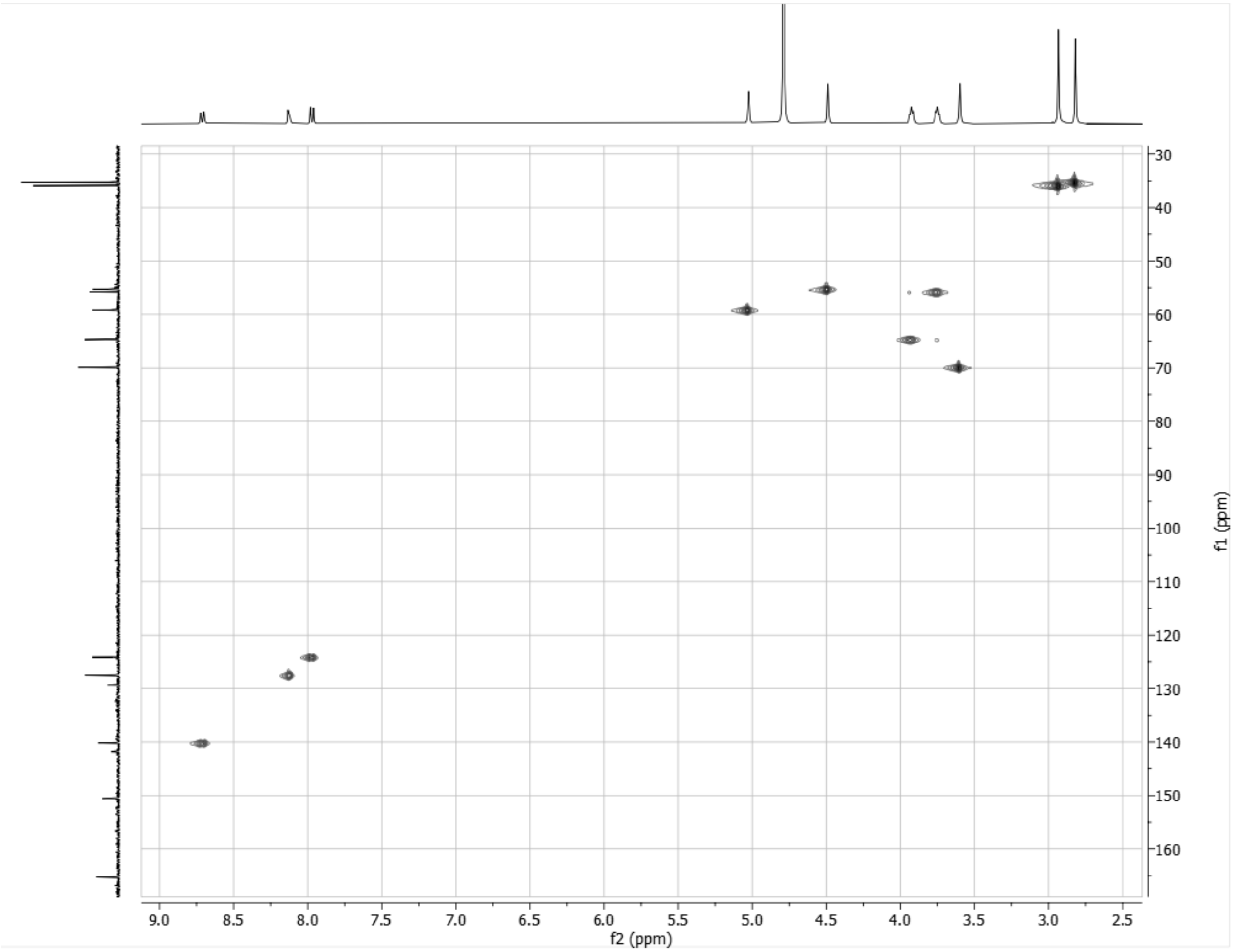

Figure S75 – ^{1}H-^{13}C HSQC of **PhenDMA**

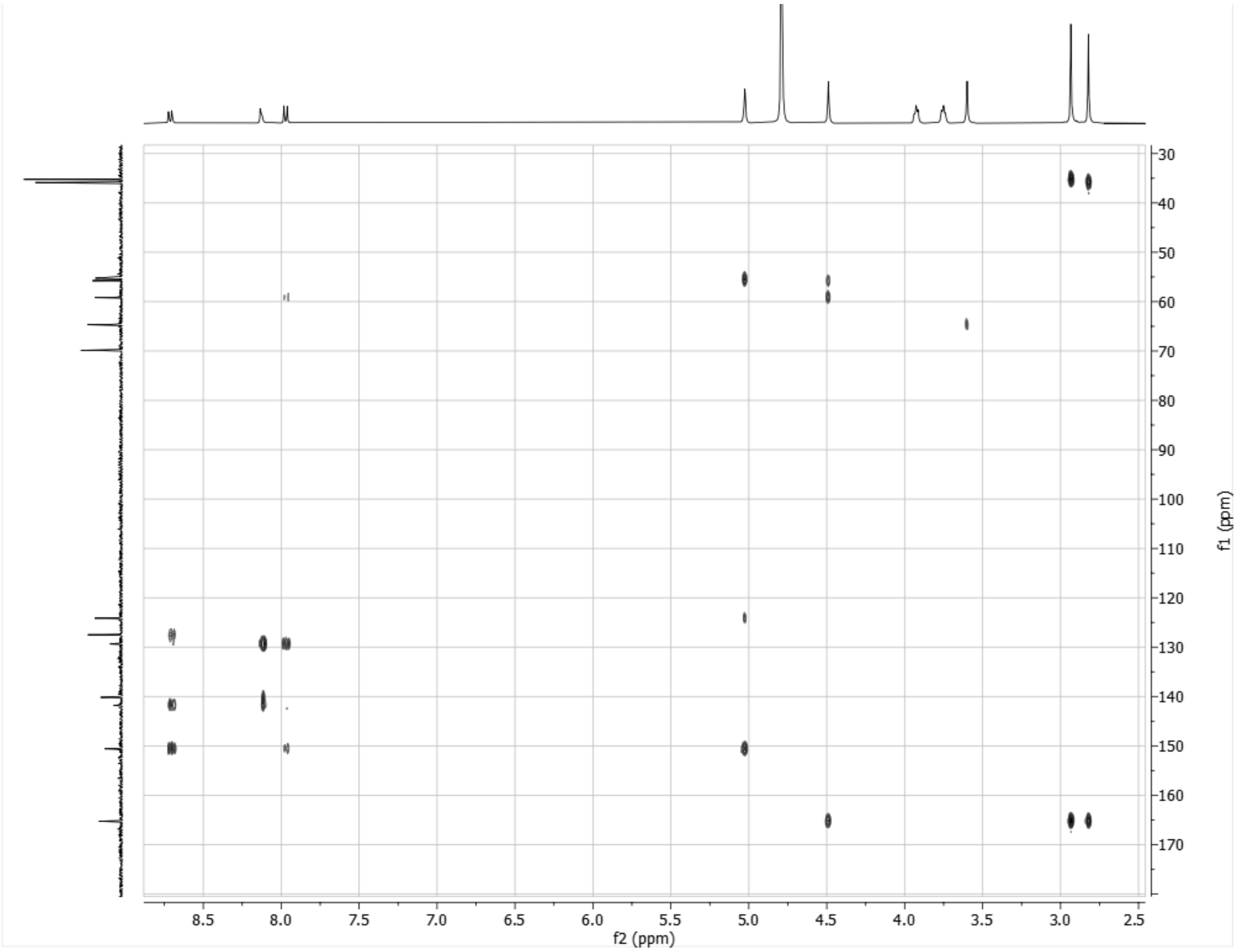

Figure S76 – ^{1}H-^{13}C HMBC of **PhenDMA**

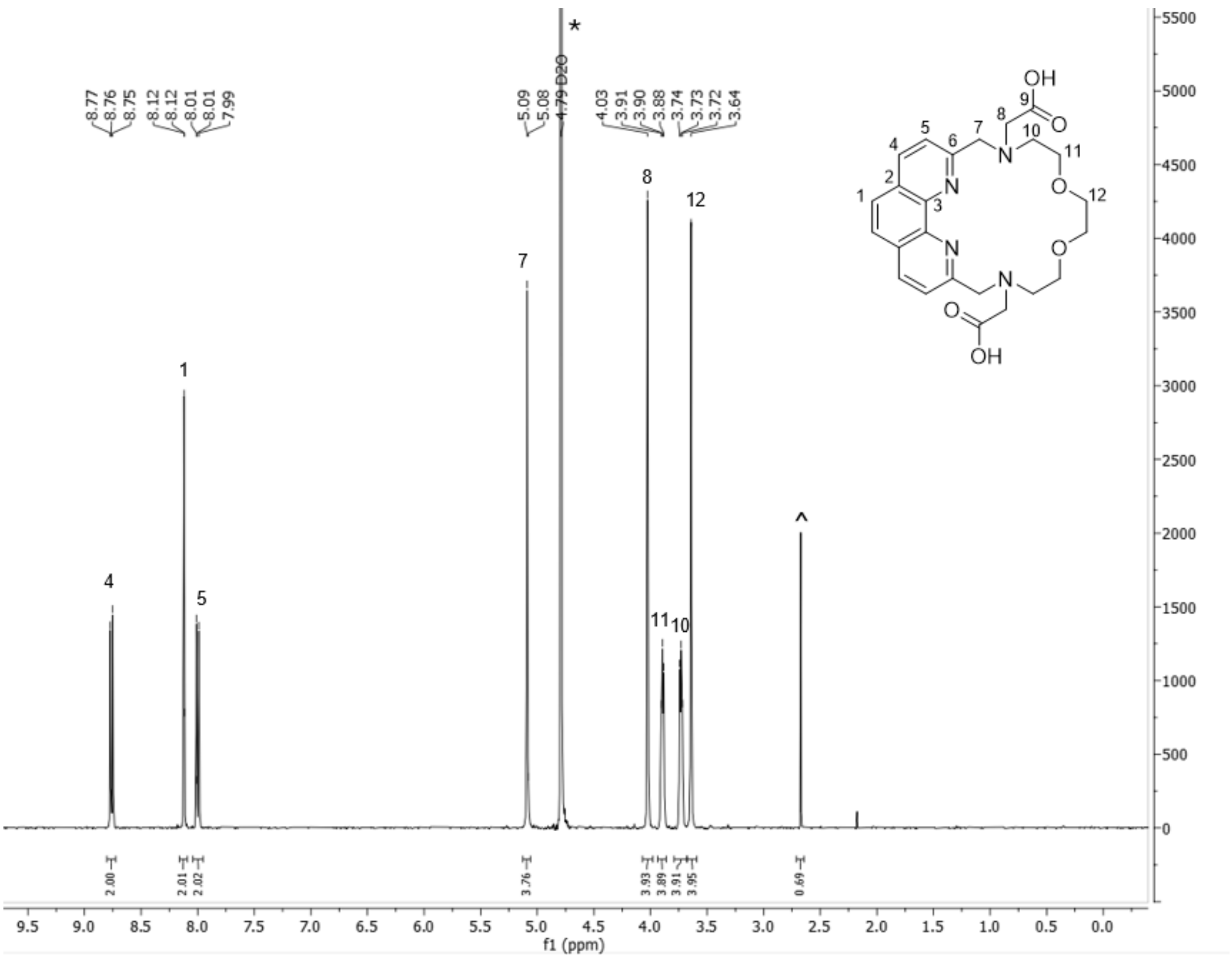

Figure S77 – ^{1}H-NMR spectrum of **phenbisacetate** in D_2O (* = D_2O; ^ = solvent impurity)

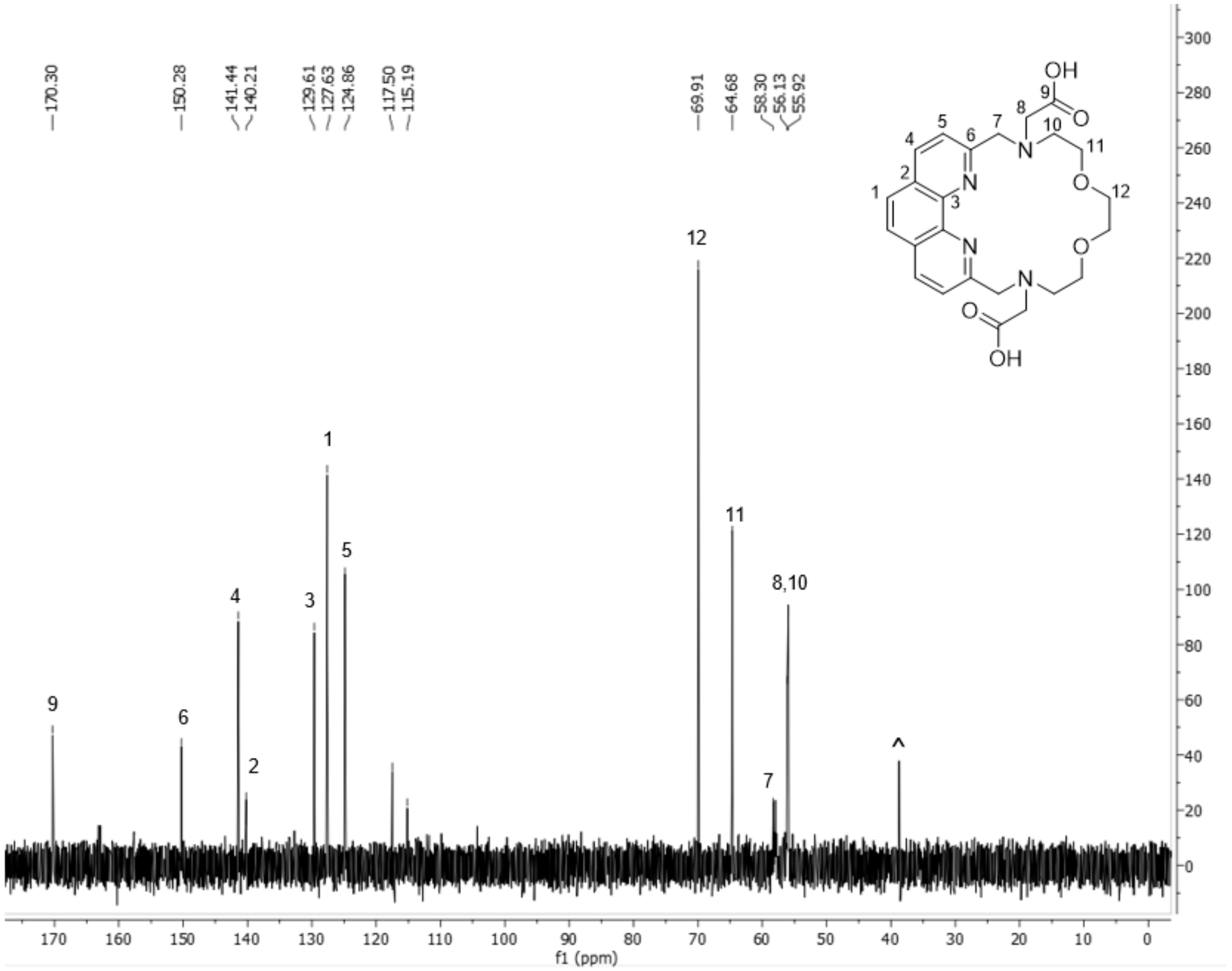

Figure S78 – ^{13}C-NMR spectrum of **phenbisacetate** in D_2O (^ = solvent impurity)

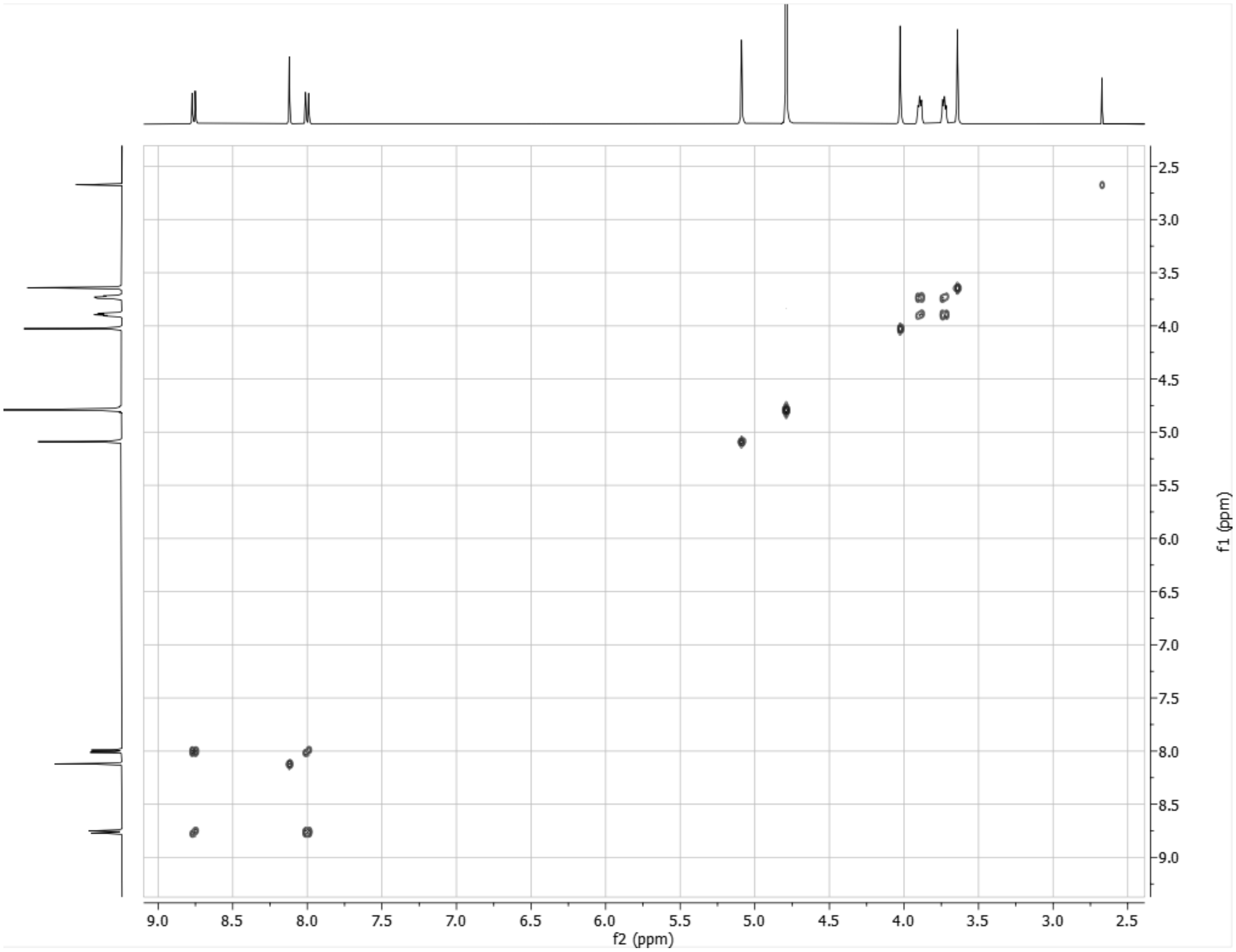

Figure S79 – ^1H-^1H COSY of **phenbisacetate** in D_2O.

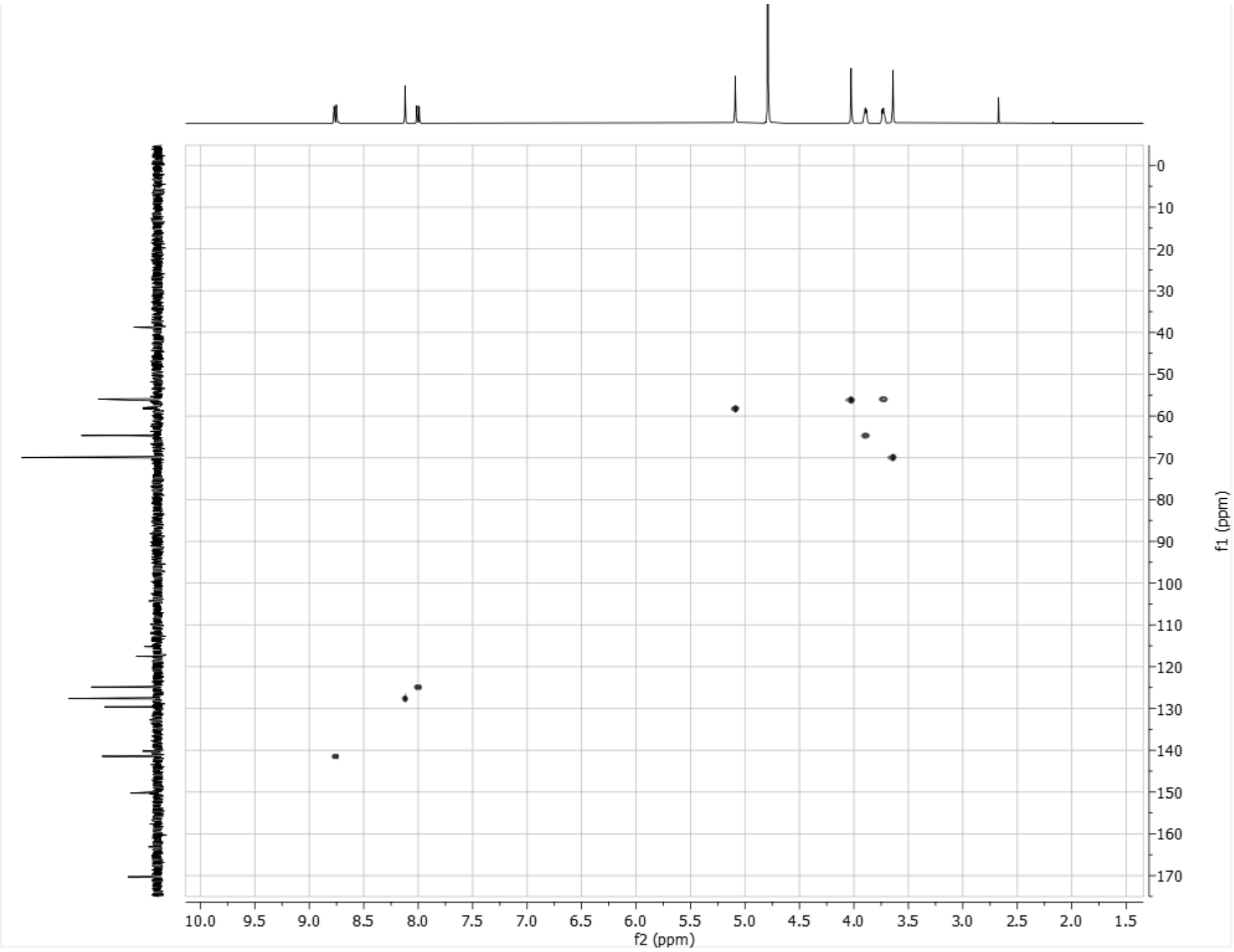

Figure S80 – ^{1}H-^{13}C HSQC spectrum of **phenbisacetate** in D_2O.

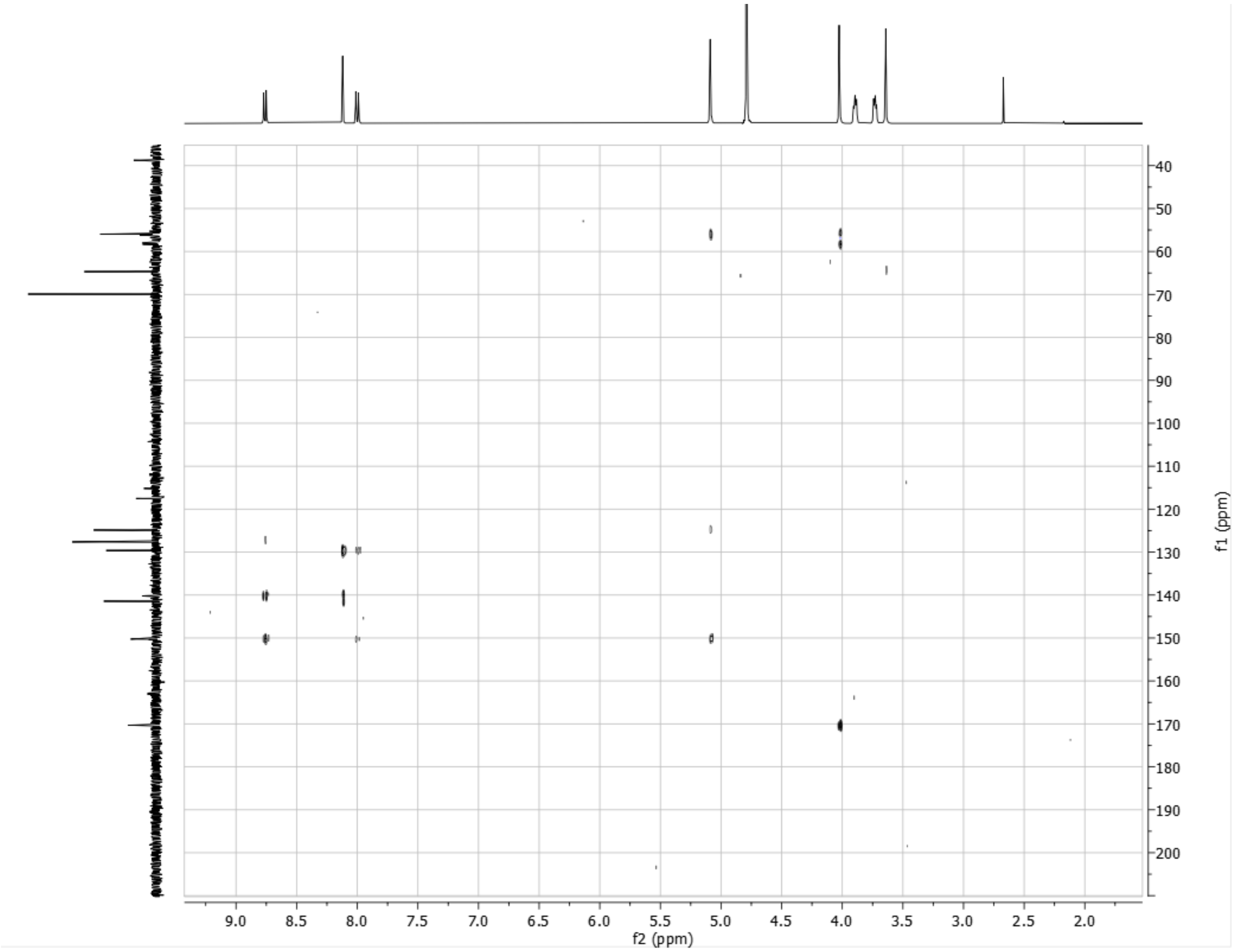

Figure S81 – ^{1}H-^{13}C HMBC spectrum of **phenbisacetate** in D_2O.

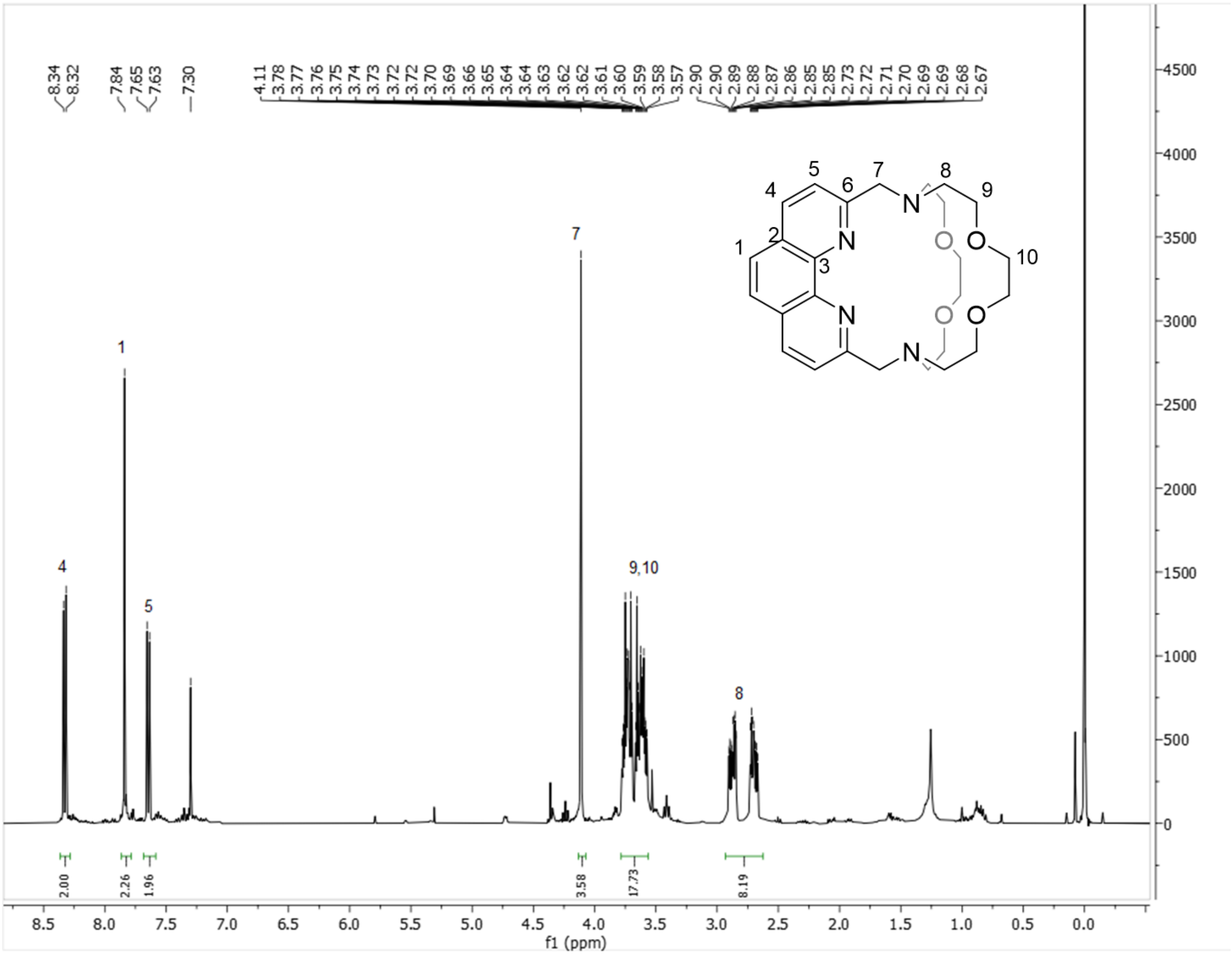

Figure S82 – ^{1}H-NMR spectrum of **phencrypt** in $CDCl_3$

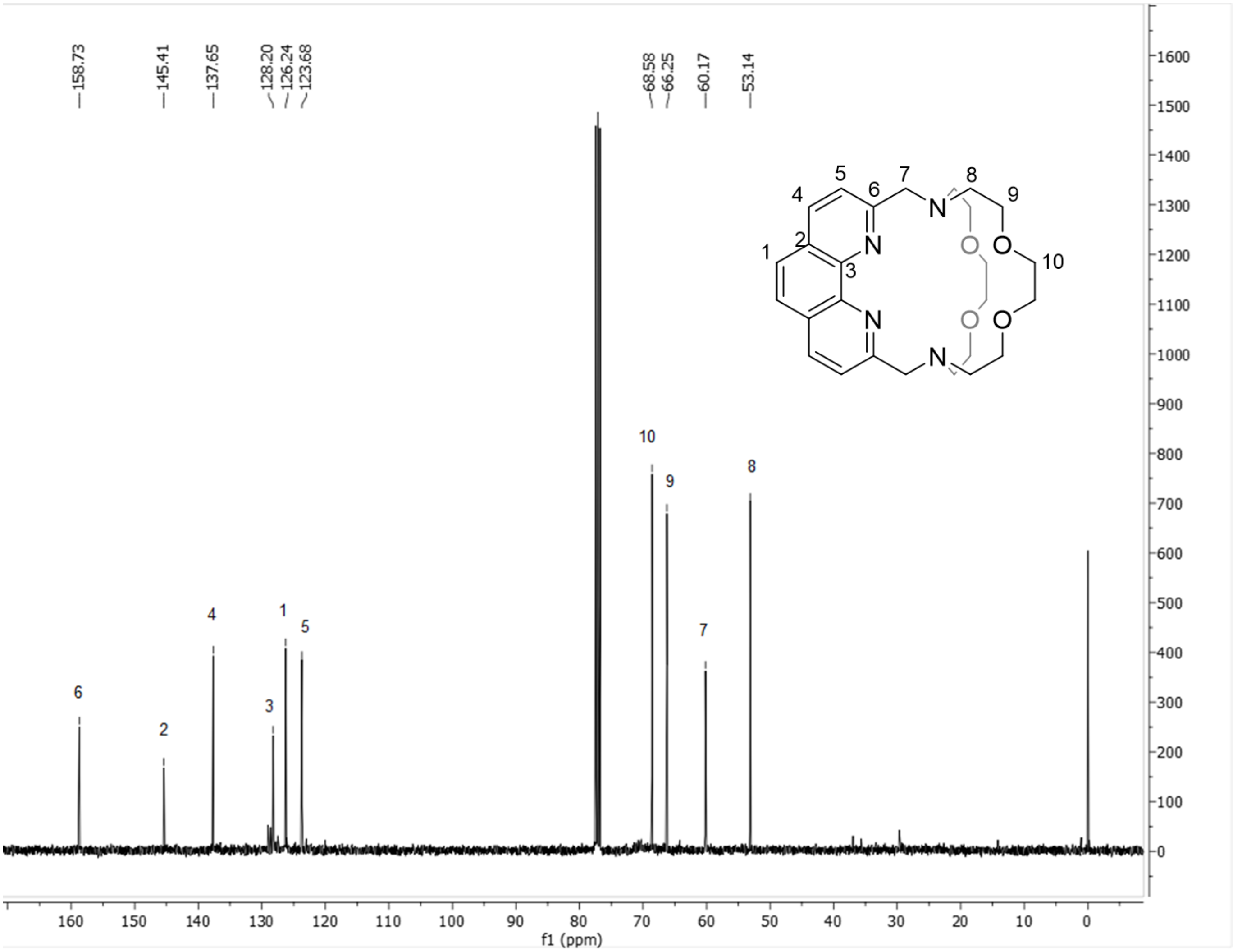

Figure S83 – ^{13}C-NMR spectrum of **phencrypt** in $CDCl_3$.

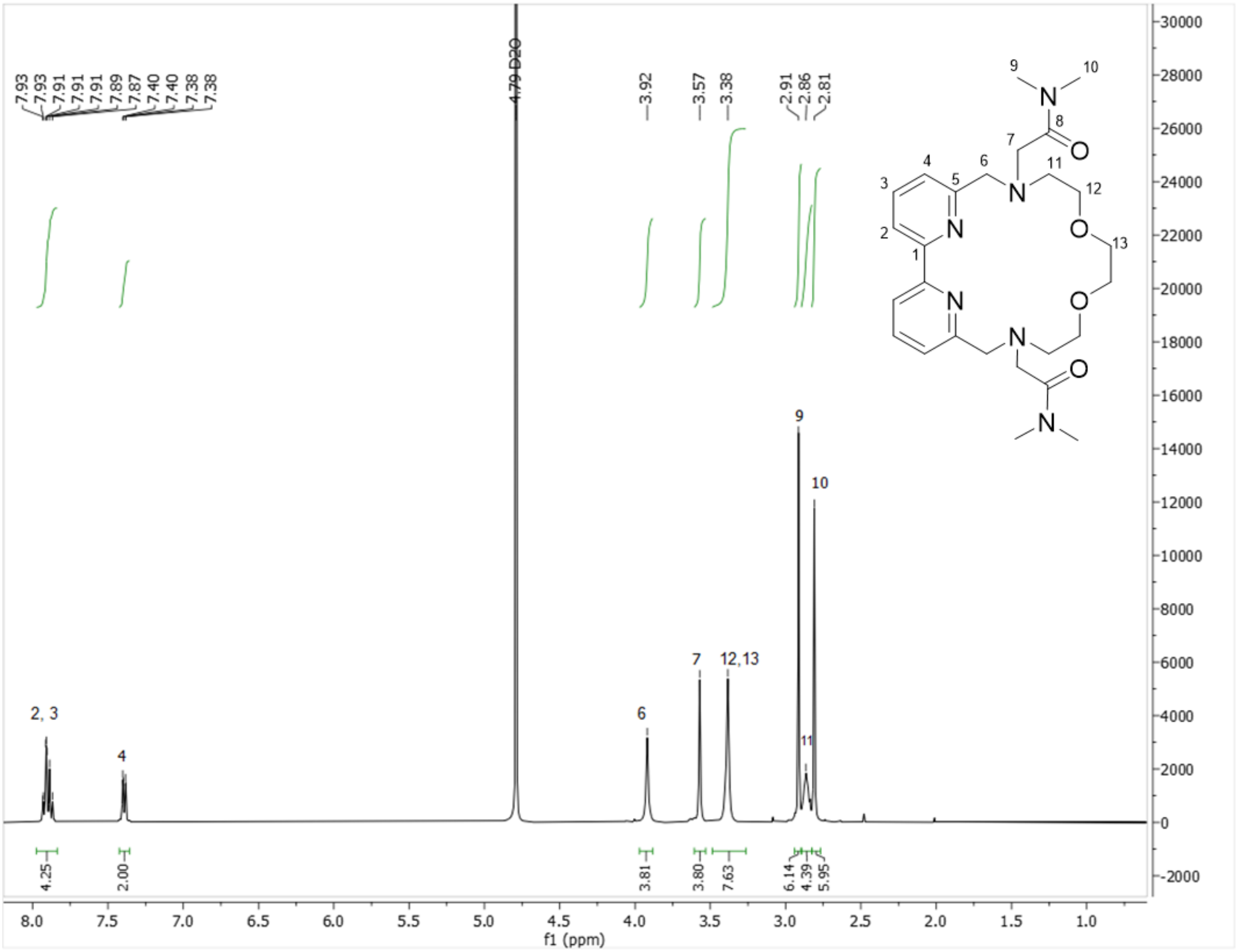

Figure S84 – ^{1}H-NMR spectrum of **BipyDMA**, 400 MHz, D_2O (*).

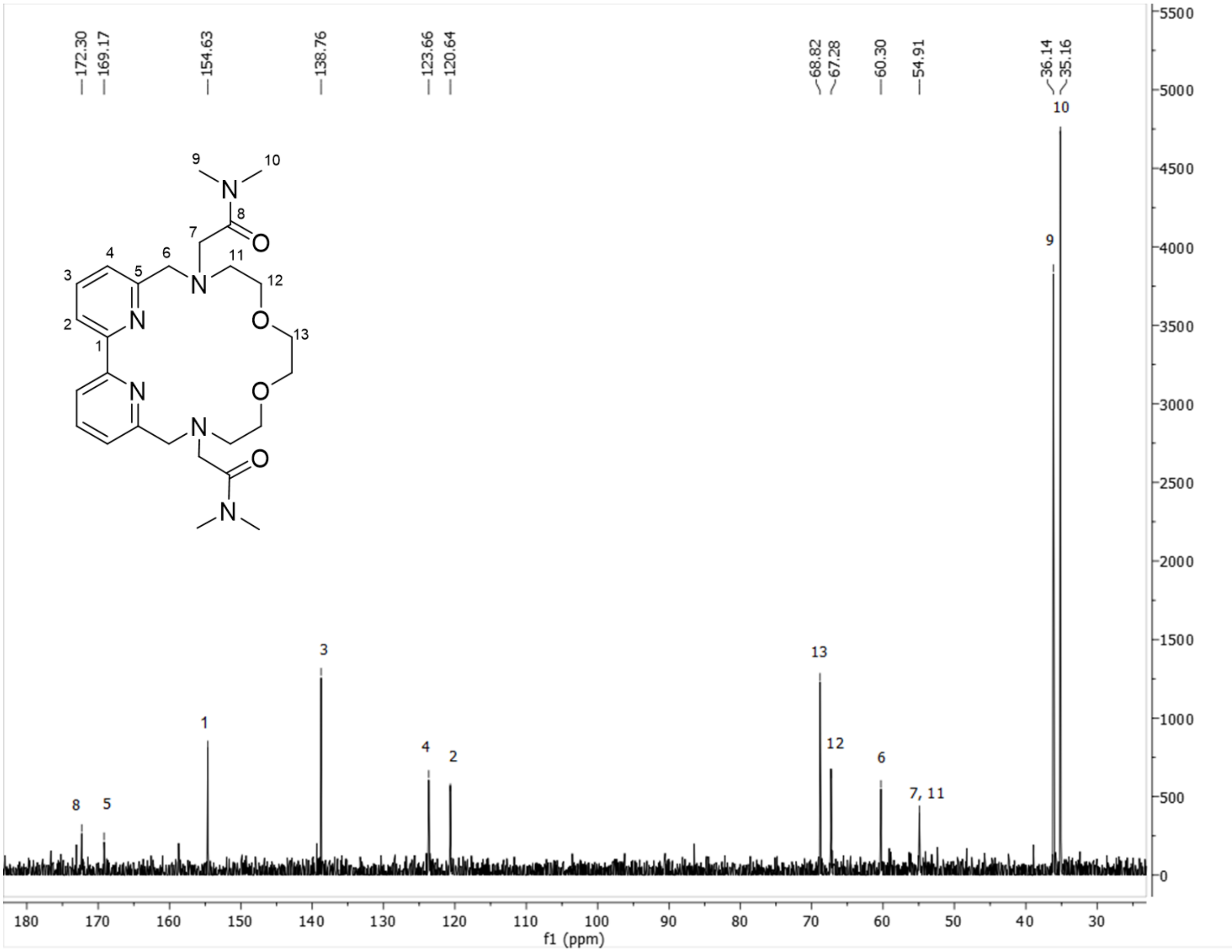

Figure S85 – ^{13}C-NMR spectrum of **bipydma** ligand

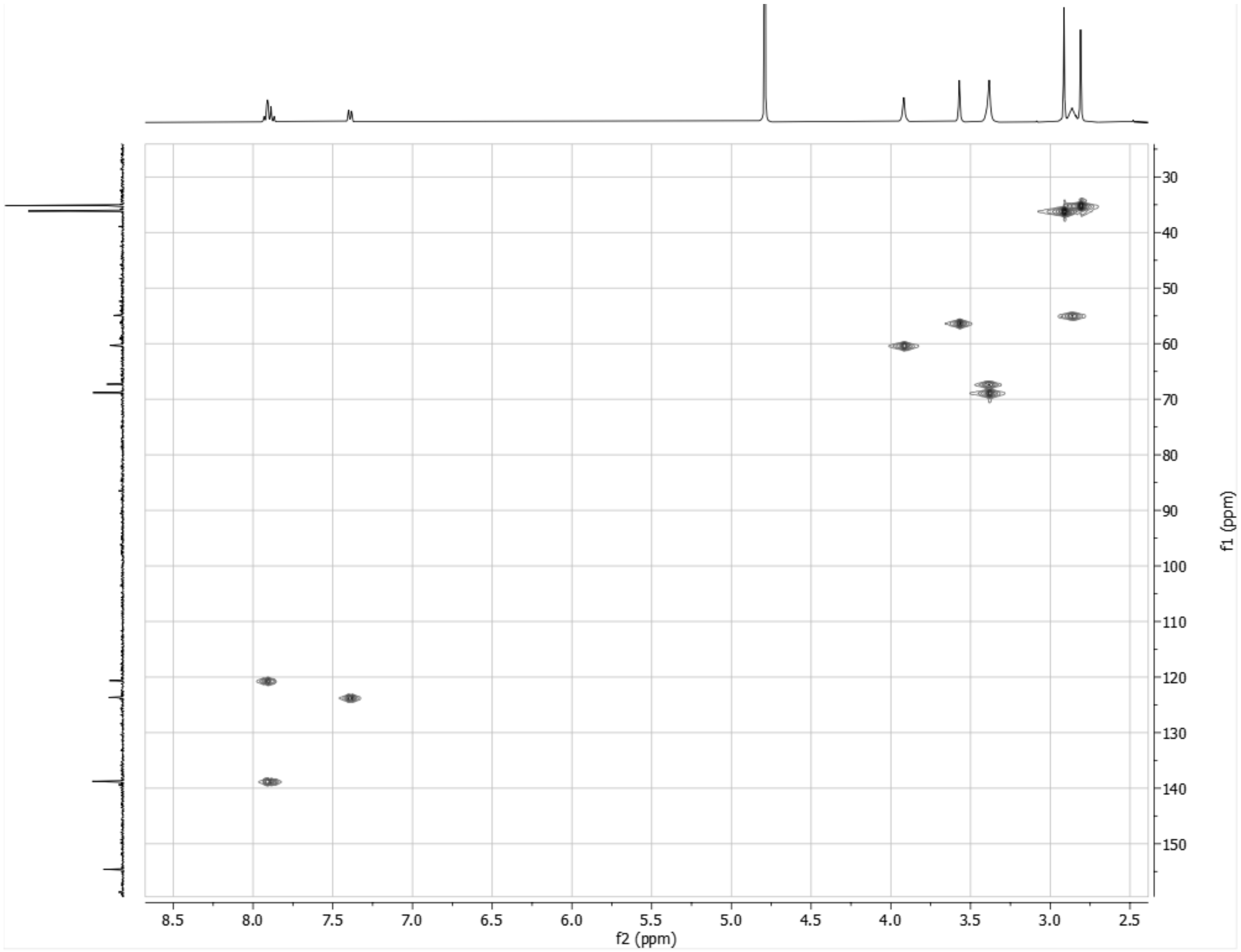

Figure S86 – ^{1}H-^{13}C HSQC of **Bipydma** ligand.

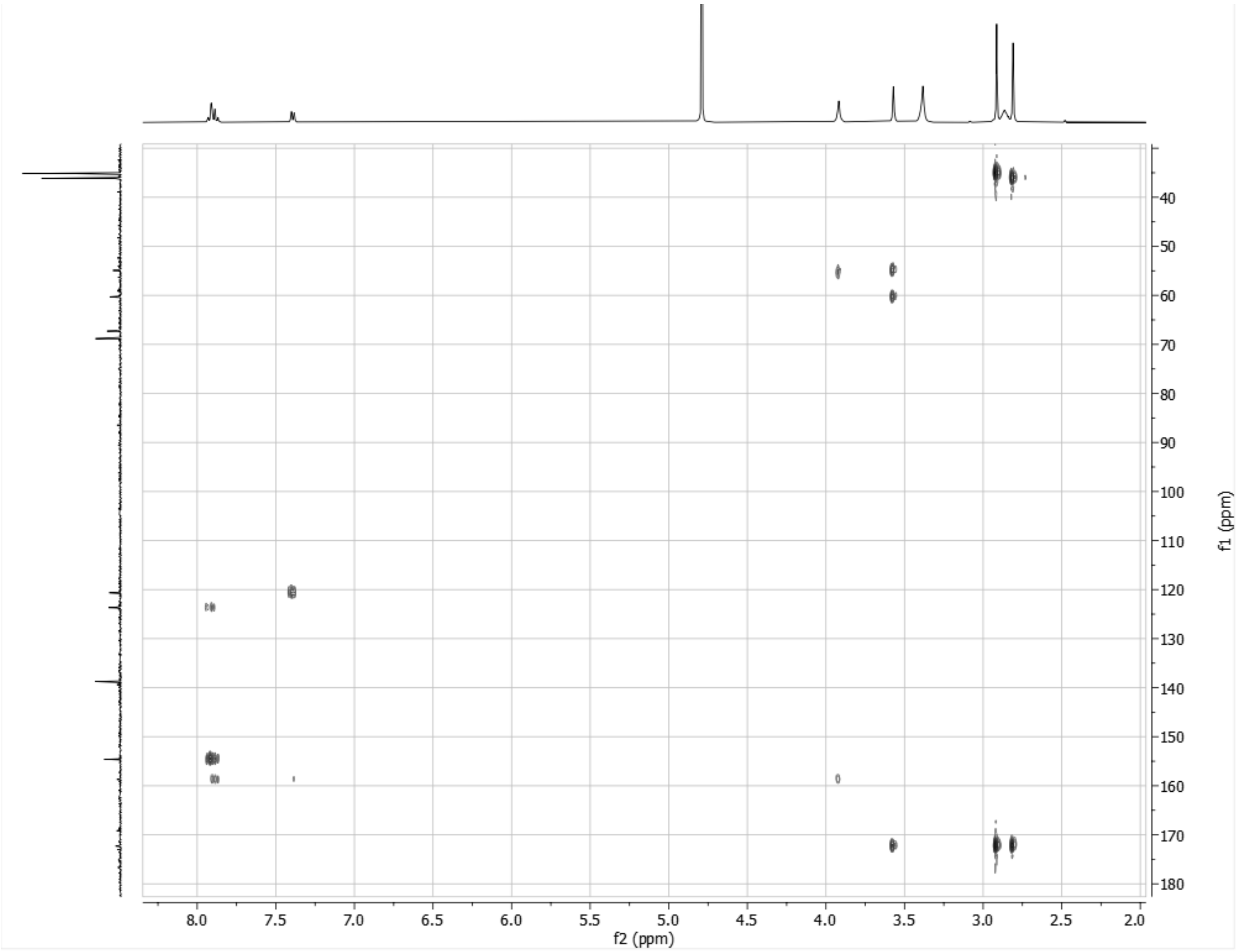

Figure S87 – ^{1}H-^{13}C HMBC of **Bipydma** ligand

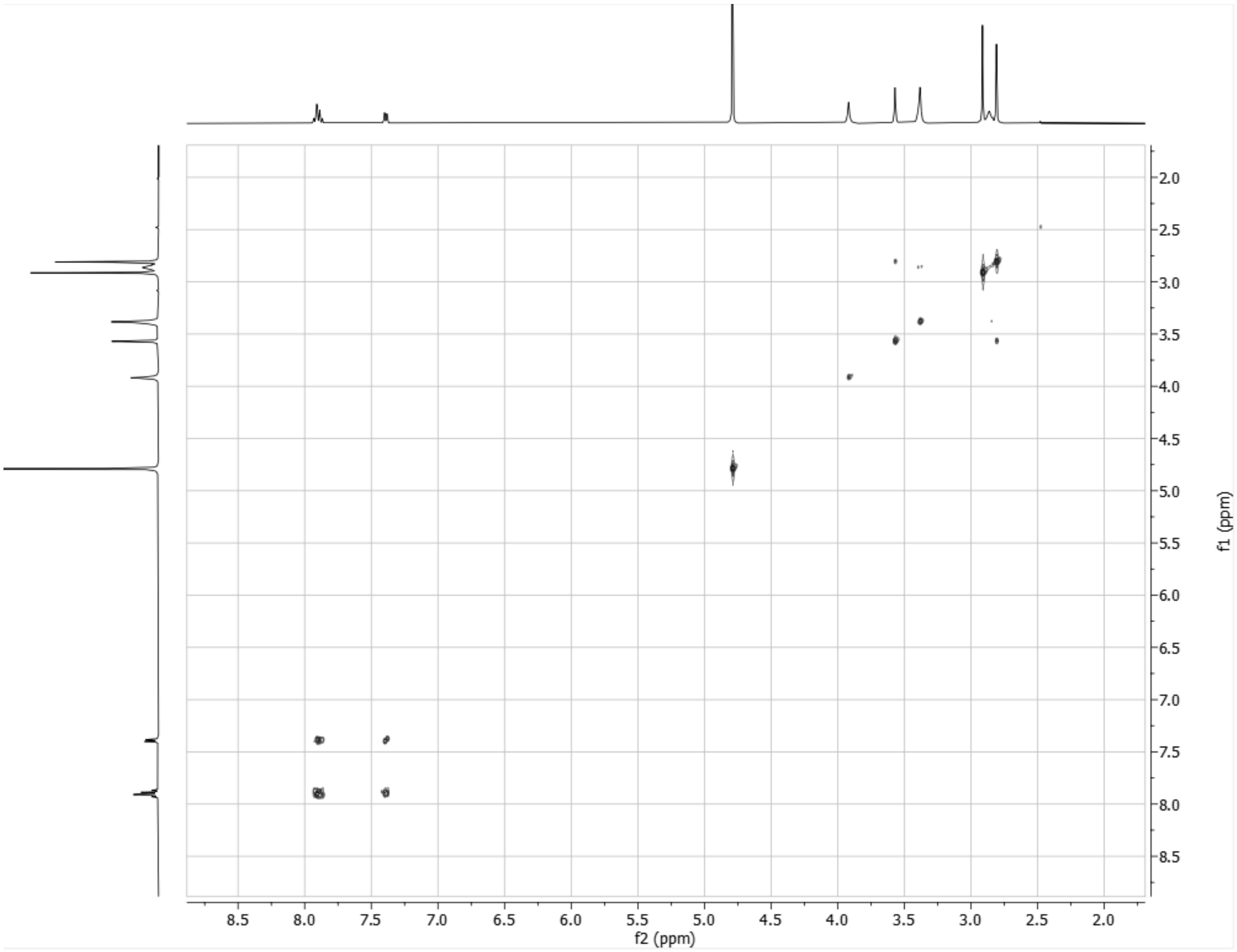

Figure S88 – ^{1}H-^{1}H COSY of **Bipydma** in D_2O.

11. Solid-State Characterization

The SolidG software package[12] was used to calculate the solid angles of the ligands. The resulting G-values describe the percentage of the metal center that is shielded by the ligand. CIF files generated from single crystal XRD was used as the input. SHAPE[13] analysis was performed by analyzing the coordination polyhedron that were derived from the CIF files generated from single crystal XRD. The polyhedron was compared to the appropriate reference polyhedron (nine or ten-coordinate) to find the lowest deviation.

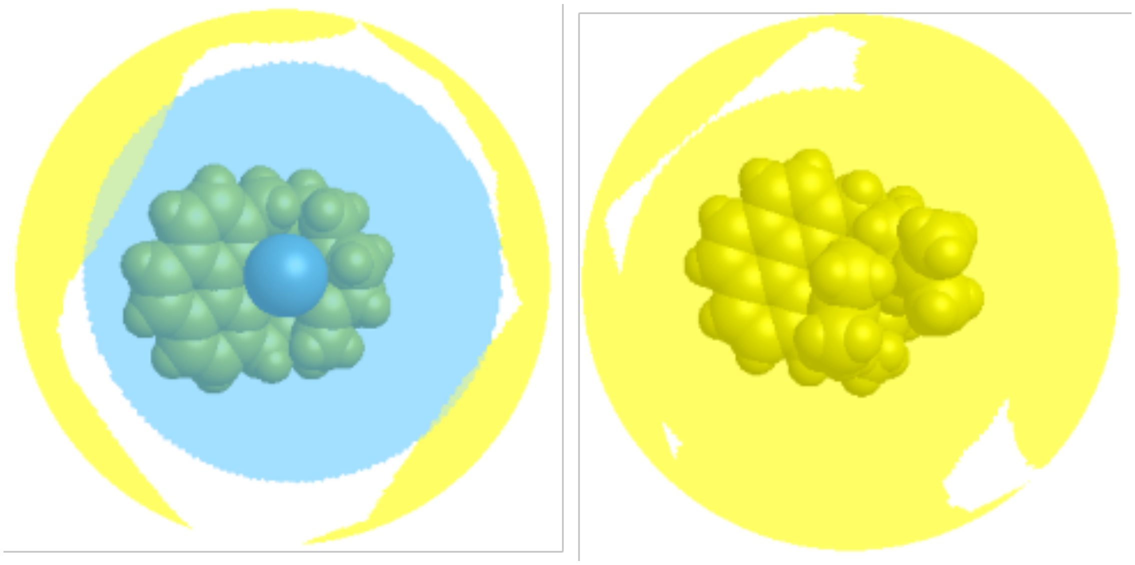

Figure S89 – Generated model showing the shielding the coordination sphere of the ligand in the crystal structure of [Eu(**PhenDMA**)Br]OTf_2, (yellow = octadentate ligand, blue = inner-sphere halide). The **PhenDMA** ligand shields 80.40% of the sphere while the inner sphere bromide shields 11.43%.

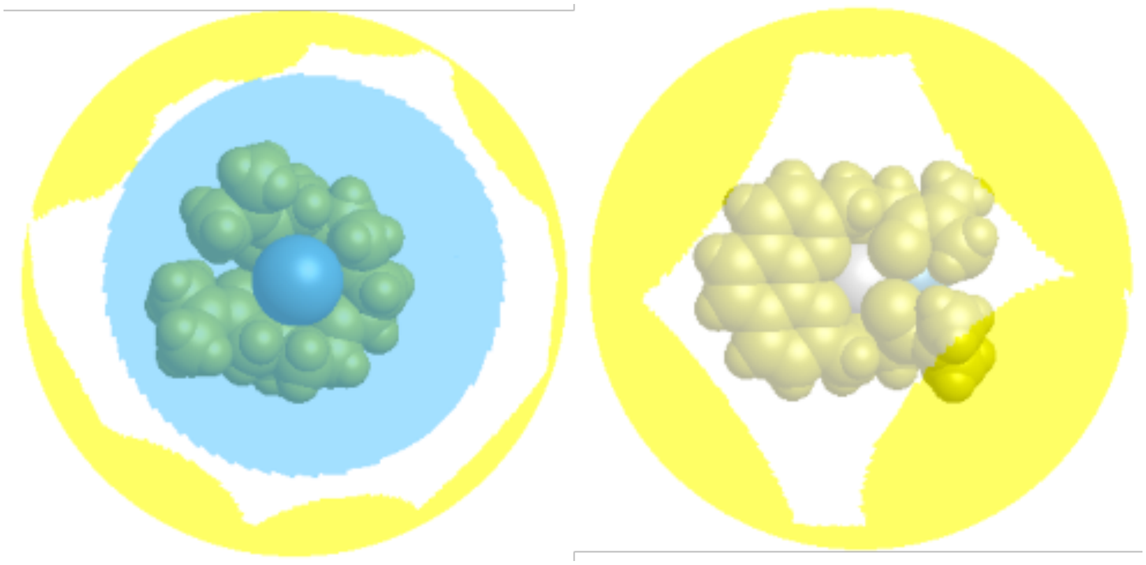

Figure S90 – Generated model showing the shielding the coordination sphere of the ligand in the crystal structure of [Eu(**PhenDMA**)I]I, (yellow = octadentate ligand, blue = inner-sphere halide). The PhenDMA ligand shields 73.16% of the sphere while the inner sphere iodide shields 9.62%.

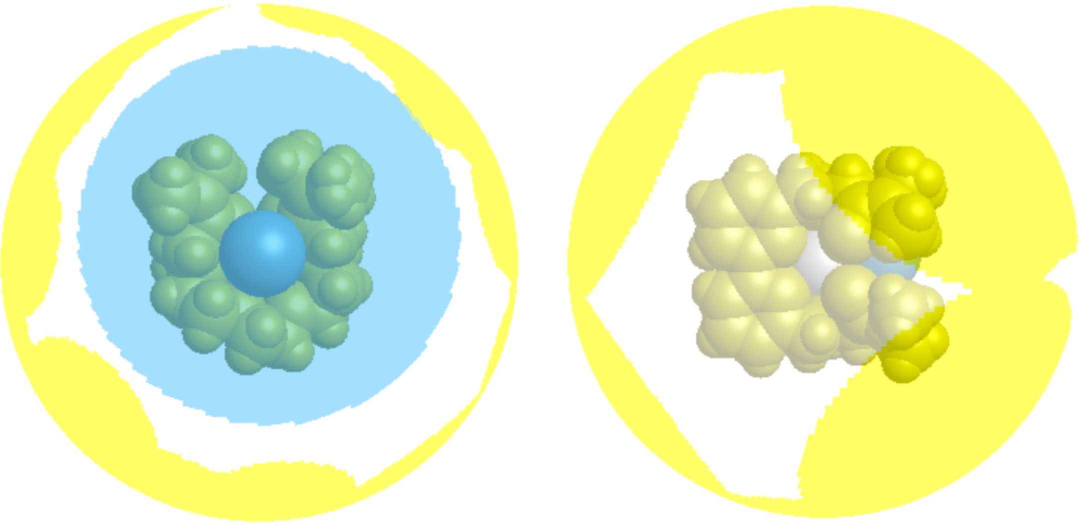

Figure S91 – Generated model showing the shielding the coordination sphere of the ligand in the crystal structure of [Eu(**BipyDMA**)I]I, (yellow = octadentate ligand, blue = inner-sphere halide). The PhenDMA ligand shields 71.78% of the sphere while the inner sphere iodide shields 9.35%.

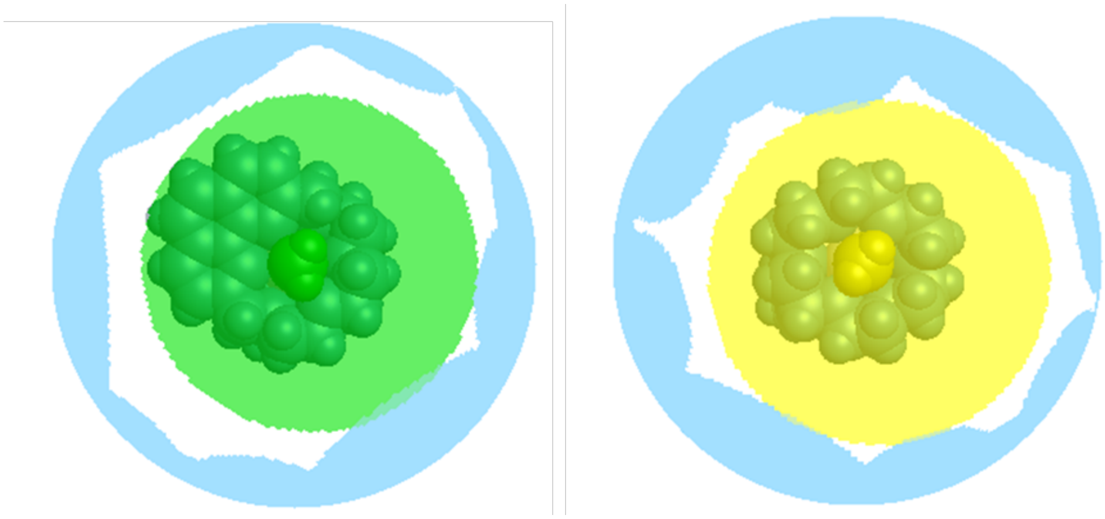

Figure S92 – Generated model showing the shielding the coordination sphere of the ligand in the crystal structure of [Eu(**Phencrypt**)(H_2O)$_2$]I_2, (blue = octadentate ligand, yellow and green = inner-sphere waters). The Phencrypt ligand shields 70.20% of the sphere while the inner sphere waters shield 8.80 and 8.65%.

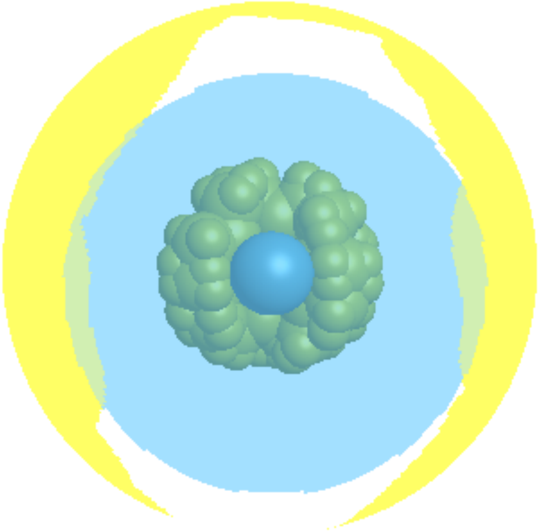

Figure S93 – Generated model showing the shielding the coordination sphere of the ligand in the crystal structure of [Eu(**Phencrypt**)Br]Br_2, (blue = octadentate ligand, yellow and green = inner-sphere waters). The Phencrypt ligand shields 80.02% of the sphere while the inner-sphere water shields 12.06%.

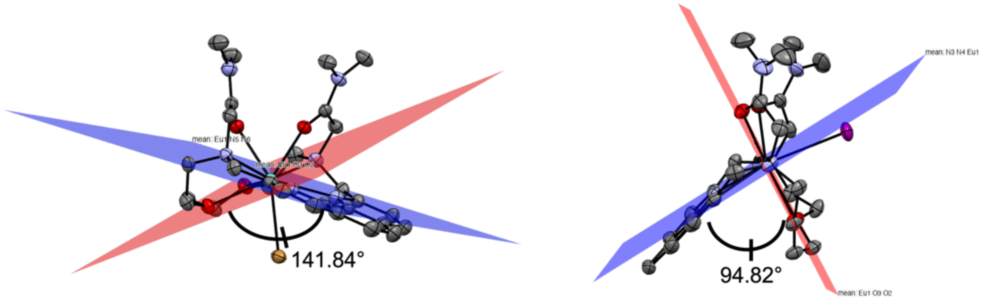

Figure S94 – Visualization of the macrocycle folding. (left) [Eu(**PhenDMA**)Br]OTf_2 and (right) [Eu(**PhenDMA**)I]I. Using Mercury, two planes are formed using the Eu-phen plane (blue) and the Eu-ethereal oxygens plane (red), then the angle between the planes are measured to address the folding of the macrocycle.

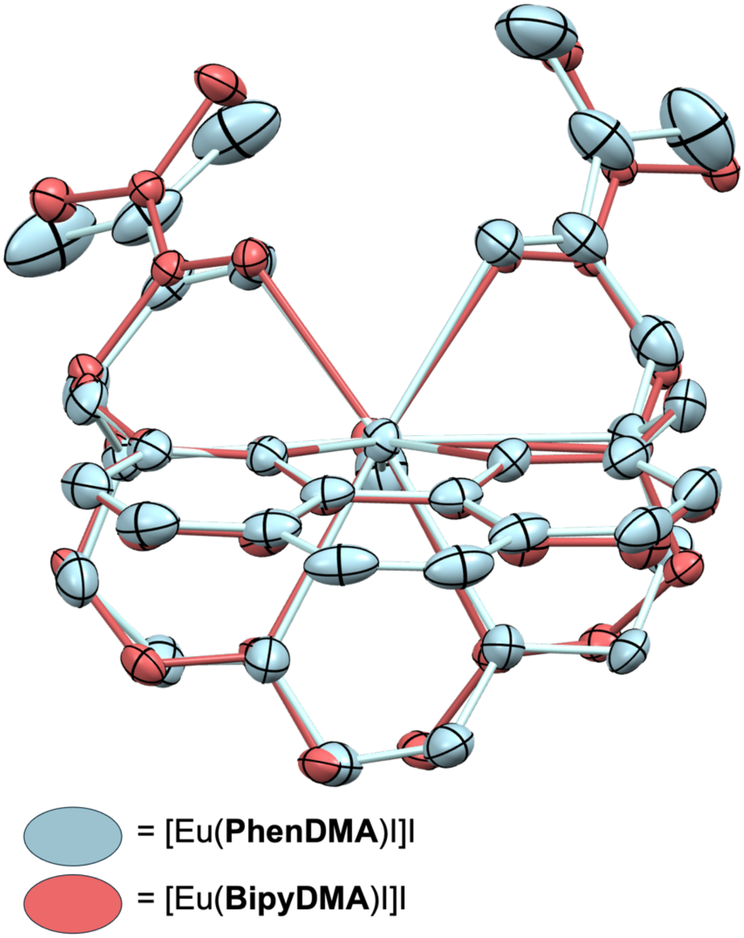

Figure S95 – Overlay of [Eu(**PhenDMA**)I]I and [Eu(**BipyDMA**)I]I highlighting the structural homology.

Table S3 – Summary of referenced divalent europium compounds previously reported

	$Eu_2(Phen)_6Br_2$ [14]	$[Eu(N_2O_6)]ZnBr_4$ [15]
$Eu–N^{phen}$	2.693 (2) – 2.808 (2)	Not applicable
$Eu–O^{ether}$	not applicable	2.717
$Eu–O^{methanol}$	not applicable	2.678

11.1 Crystallographic Information for [Eu(PhenDMA)Br]OTf_2

Data Collection

A colorless crystal with approximate dimensions 0.12 mm × 0.08 mm × 0.03 mm was selected under oil under ambient conditions and attached to the tip of a MiTeGen MicroMount©. The crystal was mounted in a stream of cold nitrogen at 100(1) K and centered in the X-ray beam by using a video camera.

The crystal evaluation and data collection were performed on a Bruker D8 VENTURE PhotonIII four-circle diffractometer with Cu Kα (λ = 1.54178 Å) radiation and the detector to crystal distance of 5.0 cm.[16]

The initial cell constants were obtained from a 180° φ scan conducted at a 2θ = 50° angle with the exposure time of 1 second per frame. The reflections were successfully indexed by an automated indexing routine built in the APEX6 program. The final cell constants were calculated from a set of 9,857 strong reflections from the actual data collection.

The data were collected by using the full sphere data collection routine to survey the reciprocal space to the extent of a full sphere to a resolution of 0.80 Å. A total of 47,405 data were harvested by collecting 50 sets of frames with 0.9–1.0º scans in ω and φ with an exposure time 1–90 s per frame. These highly redundant datasets were corrected for Lorentz and polarization effects. The absorption correction was based on fitting a function to the empirical transmission surface as sampled by multiple equivalent measurements.[17]

Structure Solution and Refinement

The systematic absences in the diffraction data were consistent for the space groups $P\bar{1}$ and $P1$. The E-statistics strongly suggested the centrosymmetric space group $P\bar{1}$ that yielded chemically reasonable and computationally stable results of refinement.[18–23]

A successful solution by intrinsic phasing provided most non-hydrogen atoms from the E-map. The remaining non-hydrogen atoms were in an alternating series of least-squares cycles and difference Fourier maps. All non-hydrogen atoms were refined with anisotropic displacement coefficients except for the minor disorder component of S2 triflate. All hydrogen atoms were included in the structure factor calculation at idealized positions and were allowed to ride on the neighboring atoms with relative isotropic displacement coefficients.

The compound is a 9-coordinate Eu dicationic complex with two triflate counterions.

The S2 triflate is disordered over two positions with the minor component contribution of 0.10. This triflate was refined with a restrained geometry and constrained isotropic displacement coefficients.

The final least-squares refinement of 533 parameters against 8,111 data resulted in residuals R (based on F^2 for $I \geq 2\sigma$) and wR (based on F^2 for all data) of 0.0275 and 0.0754, respectively. The final difference Fourier map was featureless.

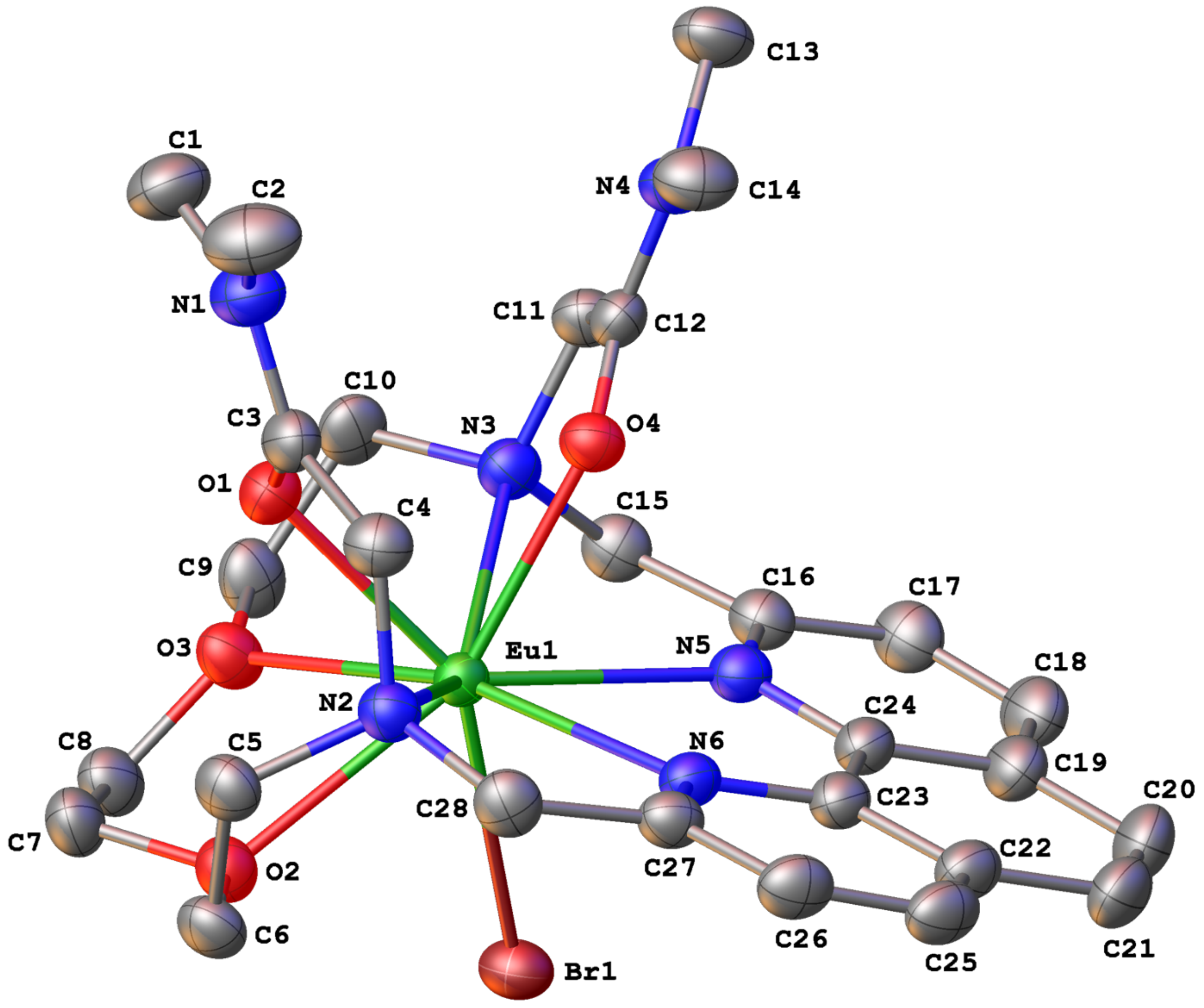

Figure S96 – A molecular drawing of the Eu dicationic complex [Eu(**PhenDMA**)Br]OTf_2 shown with 50% probability ellipsoids. All H atoms are omitted. Atom color: gray = carbon, blue = nitrogen, red = oxygen, dark red = bromide, green = europium.

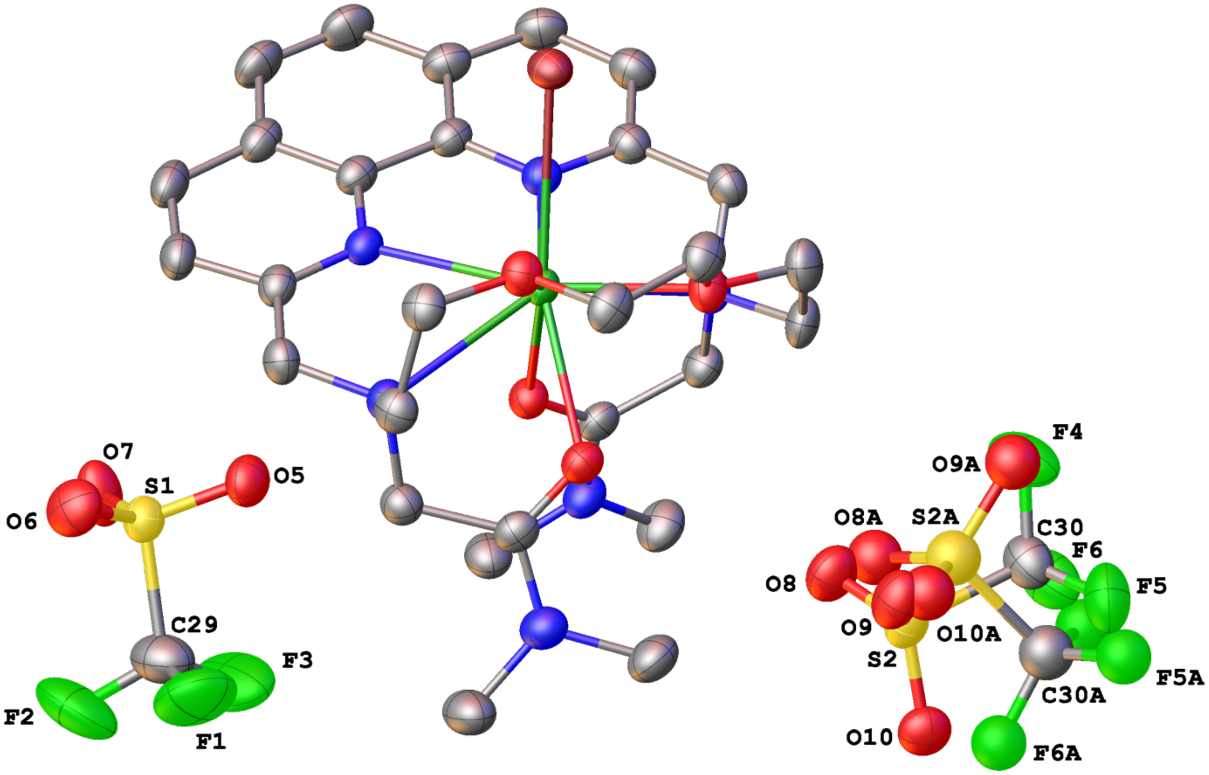

Figure S97 – A molecular drawing of [Eu(**PhenDMA**)Br]OTf$_2$ shown with 50% probability ellipsoids. Both positions of the disordered S2/S2a triflate are shown. All H atoms are omitted. Atom color: gray = carbon, blue = nitrogen, red = oxygen, dark red = bromide, lime green = fluorine, yellow = sulfur, green = europium.

Table S4 – Crystal data and structure refinement for [Eu(PhenDMA)Br]OTf₂.

Empirical formula	$[C_{28}H_{38}BrEuN_6O_4][O_3SCF_3]_2$
Formula weight	1052.65
Temperature/K	100
Crystal system	triclinic
Space group	$P\bar{1}$
a/Å	11.1437(13)
b/Å	13.1707(15)
c/Å	15.0981(17)
α/°	81.571(7)
β/°	70.777(7)
γ/°	66.006(5)
Volume/Å^3	1911.4(4)
Z	2
ρ_{calc}g/cm^3	1.829
μ/mm^{-1}	14.809
F(000)	1048.0
Crystal size/mm^3	0.12 × 0.08 × 0.03
Radiation	Cu Kα (λ = 1.54178)
2Θ range for data collection/°	9.1 to 159.242
Index ranges	$-14 \le h \le 14$, $-16 \le k \le 16$, $-19 \le l \le 18$
Reflections collected	47405
Independent reflections	8111 [R_{int} = 0.0384, R_{sigma} = 0.0283]
Data/restraints/parameters	8111/19/533
Goodness-of-fit on F^2	1.070
Final R indexes [I ≥ 2σ (I)]	R_1 = 0.0275, wR_2 = 0.0753
Final R indexes [all data]	R_1 = 0.0275, wR_2 = 0.0754
Largest diff. peak/hole / e Å^{-3}	1.15/–0.66

11.2 Crystallographic Information for [Eu(BipyDMA)I]I

Data Collection

A purple crystal with approximate dimensions of 0.129 mm × 0.051 mm × 0.048 mm was selected under oil under ambient conditions and attached to the tip of a MiTeGen MicroMount©. The crystal was mounted in a stream of cold nitrogen at 100(1) K and centered in the X-ray beam using a video camera.

Crystal evaluation and data collection were performed on a Bruker D8 VENTURE Photon III four-circle diffractometer with Diamond II IμS Mo Kα (λ = 0.71073 Å) radiation and detector-to-crystal distance of 5.0 cm.[16]

Initial cell constants were obtained from a 180° φ scan conducted at a 2θ = 30° angle with an exposure time of 1 s per frame. Reflections were indexed by an automated indexing routine built in the APEX6 program. The final cell constants were calculated from a set of 9,465 strong reflections from the actual data collection.

Data were collected by using the full sphere data collection routine to survey the reciprocal space to the extent of a full sphere to a resolution of 0.70 Å. A total of 108,753 data were harvested by collecting 17 sets of frames with 0.5–1.0º scans in ω and φ with an exposure time 1 and 4 s per frame. These highly redundant datasets were corrected for Lorentz and polarization effects. The absorption correction was based on fitting a function to the empirical transmission surface as sampled by multiple equivalent measurements.[17]

Structure Solution and Refinement

The lack of systematic absences in the diffraction data were consistent for the space groups $P\bar{1}$ and P1. E-statistics strongly suggested the centrosymmetric space group $P\bar{1}$ that yielded chemically reasonable and computationally stable results of refinement.[18–23]

A successful solution by intrinsic phasing provided most non-hydrogen atoms from the E-map. The remaining non-hydrogen atoms were in an alternating series of least-squares cycles and difference Fourier maps. All non-hydrogen atoms were refined with anisotropic atomic displacement coefficients. All hydrogen atoms were included in the structure factor calculation at idealized positions and were allowed to ride on the neighboring atoms with relative isotropic atomic displacement coefficients.

The asymmetric unit contains one $[EuC_{26}H_{38}N_6O_4I]I$ ionic pair, one molecule of acetonitrile, and one C_4H_8O solvent molecule that resides around a crystallographic inversion center. The overall molecular formula for this compound is therefore $[EuC_{26}H_{38}N_6O_4I]I{\cdot}C_2H_3N{\cdot}0.5C_4H_8O$.

H atoms of the C13 methyl group of the $[EuC_{26}H_{38}N_6O_4I]^+$ cation are disordered over two positions with half occupancy each.

The O5 C_4H_8O solvent molecule resides around a crystallographic inversion center, thus it is only half occupied in the asymmetric unit. This molecule is refined with restrained geometric parameters. Atom C32 has large anisotropic atomic displacement parameters; however, modeling this atom over multiple positions did not improve the overall refinement.

The final least-squares refinement of 429 parameters against 11,081 data resulted in residuals R (based on F^2 for $I \geq 2\sigma$) and wR (based on F^2 for all data) of 0.0152 and 0.0367, respectively. The final difference Fourier map was featureless.

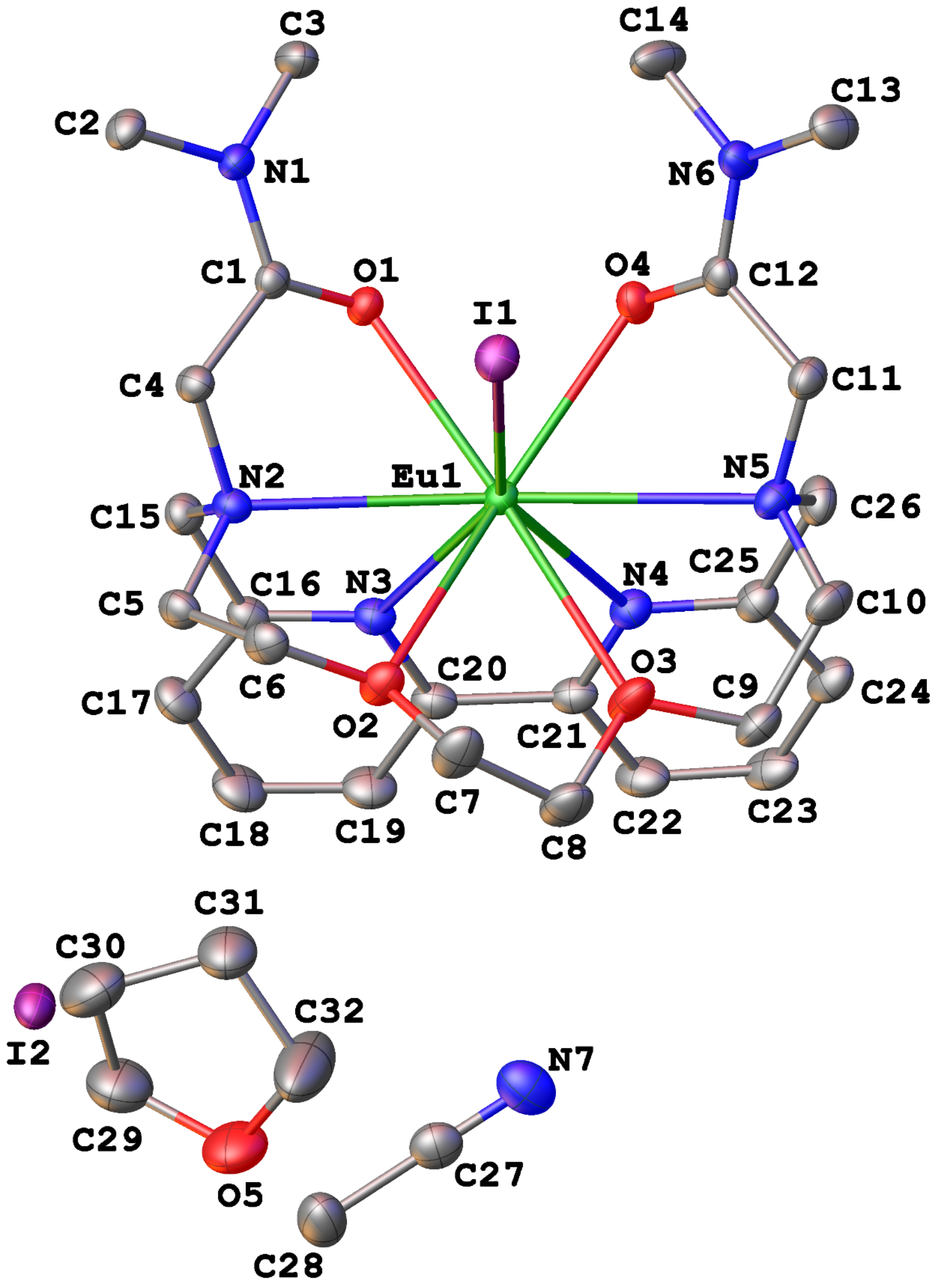

Figure S98 – Molecular drawing of [Eu(**BipyDMA**)I]I shown with 50% probability ellipsoids. The O5 C_4H_8O solvent molecule is half occupied in the asymmetric unit. All H atoms are omitted. Atom color: gray = carbon, blue = nitrogen, red = oxygen, purple = iodide, green = europium.

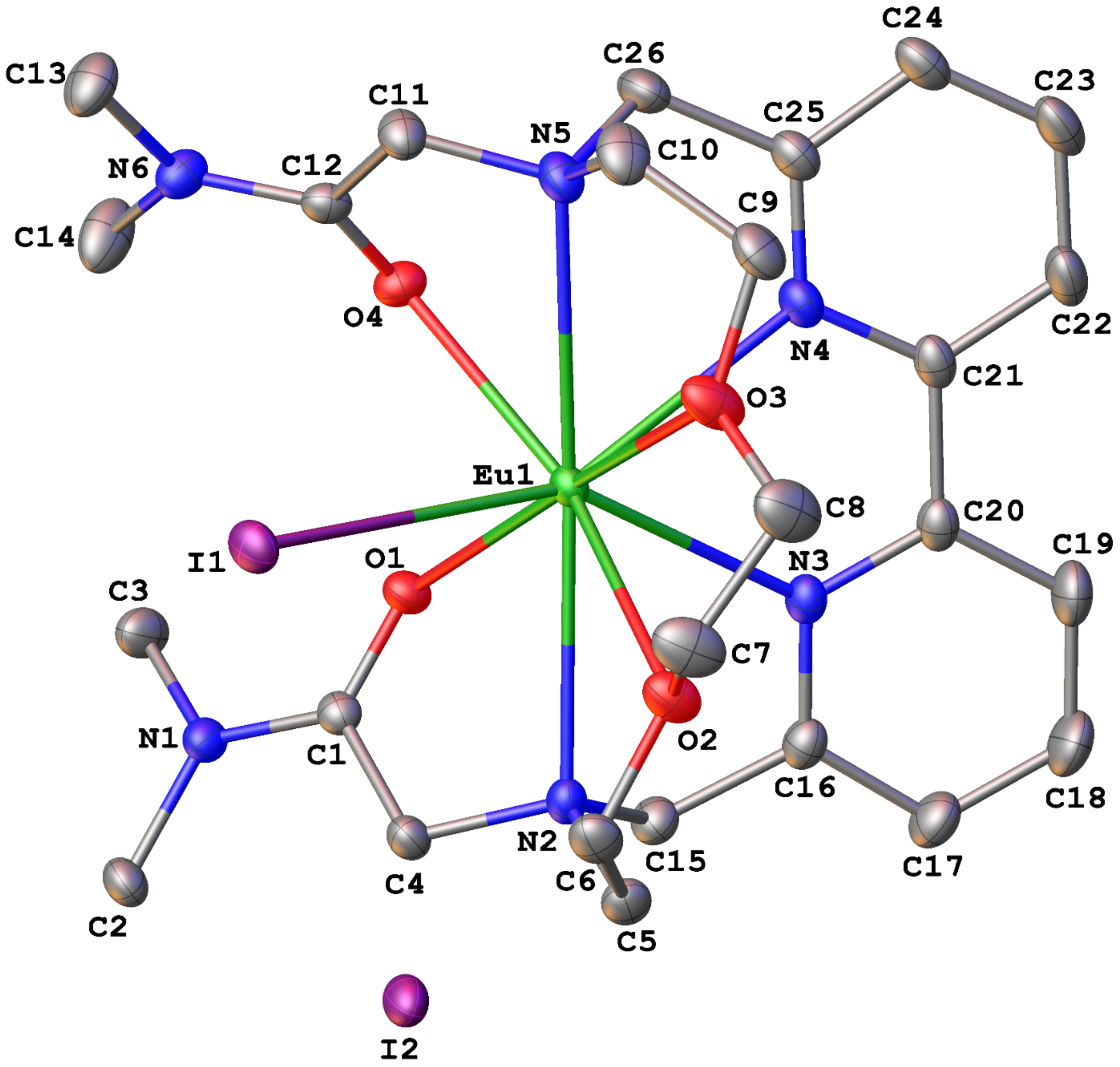

Figure S99 – A molecular drawing of the [Eu(**BipyDMA**)I]I, shown with 50% probability ellipsoids. All H atoms are omitted. Atom color: gray = carbon, blue = nitrogen, red = oxygen, purple = iodide, green = europium.

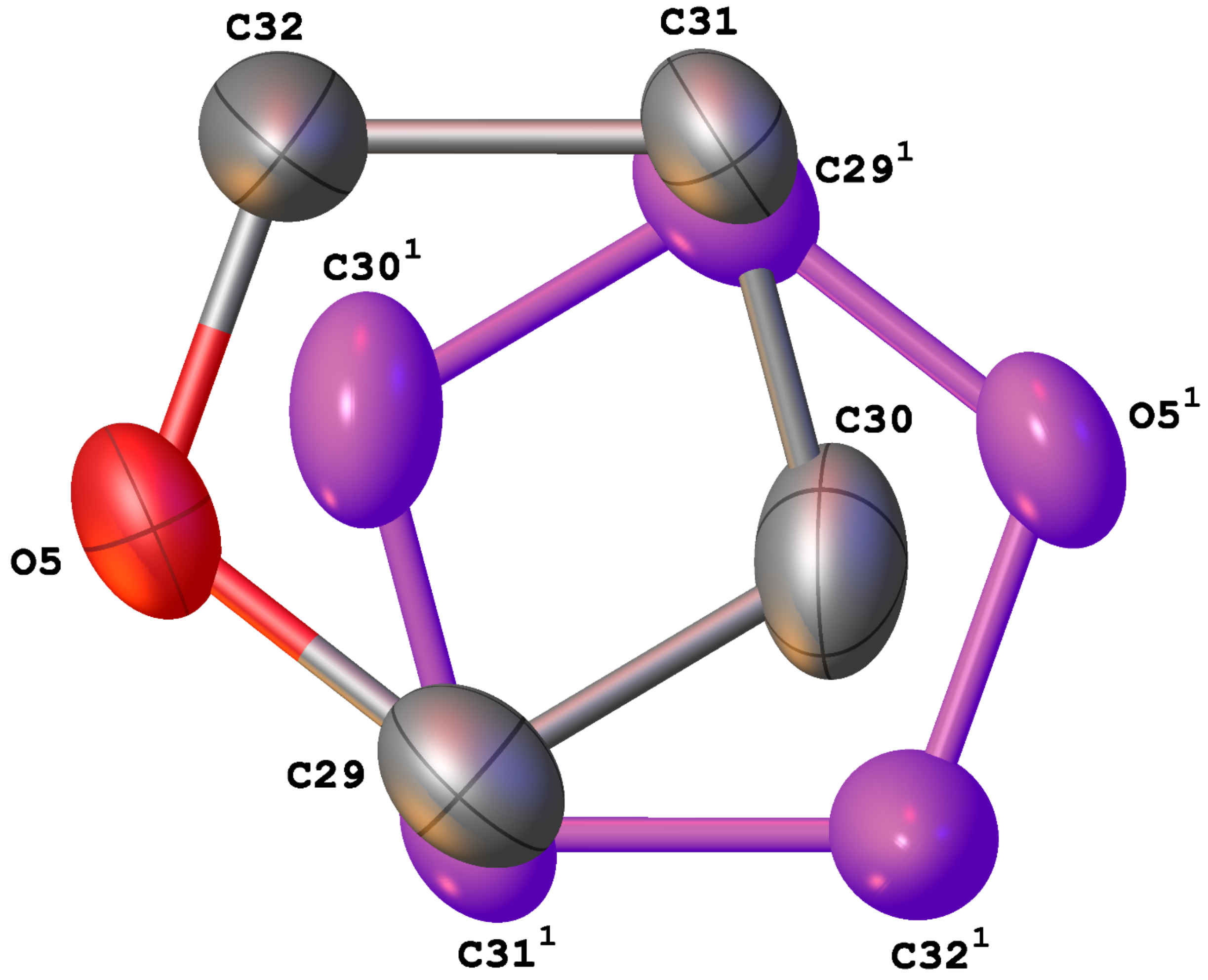

Figure S100 – Molecular drawing of the O5 C_4H_8O solvent molecule expanded about the crystallographic inversion center. The symmetry-related C_4H_8O molecule is drawn in purple. Symmetry code: (1) 2–X, 1–Y, 1–Z. Atom color: gray = carbon, red = oxygen.

Table S5 – Crystal data and structure refinement for [Eu(BipyDMA)I]I.

Empirical formula	$[EuC_{26}H_{38}N_6O_4I]I\cdot C_2H_3N\cdot 0.5C_4H_8O$
Formula weight	981.49
Temperature/K	100.00
Crystal system	triclinic
Space group	$P\bar{1}$
a/Å	11.3707(14)
b/Å	11.7898(14)
c/Å	15.533(2)
α/°	108.474(8)
β/°	103.683(9)
γ/°	102.409(11)
Volume/Å^3	1,821.6(4)
Z	2
ρ_{calc}g/cm^3	1.789
μ/mm^{-1}	3.460
F(000)	958.0
Crystal size/mm^3	0.129 × 0.051 × 0.048
Radiation	Mo Kα (λ = 0.71073)
2Θ range for data collection/°	3.82 to 61.03
Index ranges	$-16 \le h \le 16$, $-16 \le k \le 16$, $-22 \le l \le 22$
Reflections collected	108,752
Independent reflections	11,081 [R_{int} = 0.0345, R_{sigma} = 0.0169]
Data/restraints/parameters	11,081/10/429
Goodness-of-fit on F^2	1.061
Final R indexes [I ≥ 2σ (I)]	R_1 = 0.0152, wR_2 = 0.0360
Final R indexes [all data]	R_1 = 0.0163, wR_2 = 0.0367
Largest diff. peak/hole / e Å^{-3}	0.71/–0.70

11.3 Crystallographic Information for [Eu(PhenDMA)I]I

Data Collection

A brown crystal with approximate dimensions 0.06 mm × 0.03 mm × 0.01 mm was selected under oil under ambient conditions and attached to the tip of a MiTeGen MicroMount©. The crystal was mounted in a stream of cold nitrogen at 100(1) K and centered in the X-ray beam by using a video camera.

Crystal evaluation and data collection were performed on a Bruker D8 VENTURE Photon III four-circle diffractometer with Diamond II IµS Mo Kα (λ = 0.71073 Å) radiation and a detector-to-crystal distance of 5.0 cm.[16]

Initial cell constants were obtained from a 180° φ scan conducted at a 2θ = 30° angle with an exposure time of 1 s per frame. Reflections were indexed by an automated indexing routine built in the APEX6 program. Final cell constants were calculated from a set of 9,040 strong reflections from the actual data collection.

Data were collected using the half-sphere data collection routine to survey the reciprocal space to the extent of a half sphere to a resolution of 0.75 Å. A total of 94,139 data were harvested by collecting 8 sets of frames with 0.5–1.0º scans in ω and φ with an exposure time of 1–40 s per frame. These highly redundant datasets were corrected for Lorentz and polarization effects. The absorption correction was based on fitting a function to the empirical transmission surface as sampled by multiple equivalent measurements.[17]

Structure Solution and Refinement

Systematic absences in the diffraction data were uniquely consistent for the space group $P2_1/c$ that yielded chemically reasonable and computationally stable results of refinement.[18–23]

A successful solution by intrinsic phasing provided most non-hydrogen atoms from the *E*-map. The remaining non-hydrogen atoms were in an alternating series of least-squares cycles and difference Fourier maps. All non-hydrogen atoms were refined with anisotropic atomic displacement coefficients. All hydrogen atoms were included in the structure factor calculation at idealized positions and were allowed to ride on the neighboring atoms with relative isotropic atomic displacement coefficients.

The asymmetric unit contains one cation with a nine-coordinate europium, $[Eu(C_{28}H_{38}N_6O_4)I]^+$, that is charge-balanced by an iodide anion. There is also one partially occupied water molecule for an overall chemical formula of $[Eu(C_{28}H_{38}N_6O_4)I]I \cdot 0.844H_2O$.

The $C_{28}H_{38}N_6O_4$ ligand coordinated to the Eu contains one fragment with positional disorder. The C8, O3, C9, and C10 chain of atoms is disordered over two positions with a dominant component occupation of 78.2(7)%. The minor disordered component is refined with restrained geometries and restrained anisotropic atomic displacement coefficients.

The I1 atom coordinated to Eu is disordered over two positions with a dominant component occupation of 97.10(10)%. The anisotropic atomic displacement coefficients of the major and minor component are constrained to be the same.

The I2$^-$ anion is disordered over two positions with a dominant component occupation of 83.7(11)%.

The solvent water molecule is occupationally disordered with a partial occupancy of 84.4(10)%.

The final least-squares refinement of 438 parameters against 8,190 data resulted in residuals R (based on F^2 for $I \geq 2\sigma$) and wR (based on F^2 for all data) of 0.0318 and 0. 0710, respectively. The final difference Fourier map contains residual peaks of electron density of 1.35 e/Å^3 near the Eu and I atoms. These peaks are in chemically unreasonable positions and are therefore considered noise.

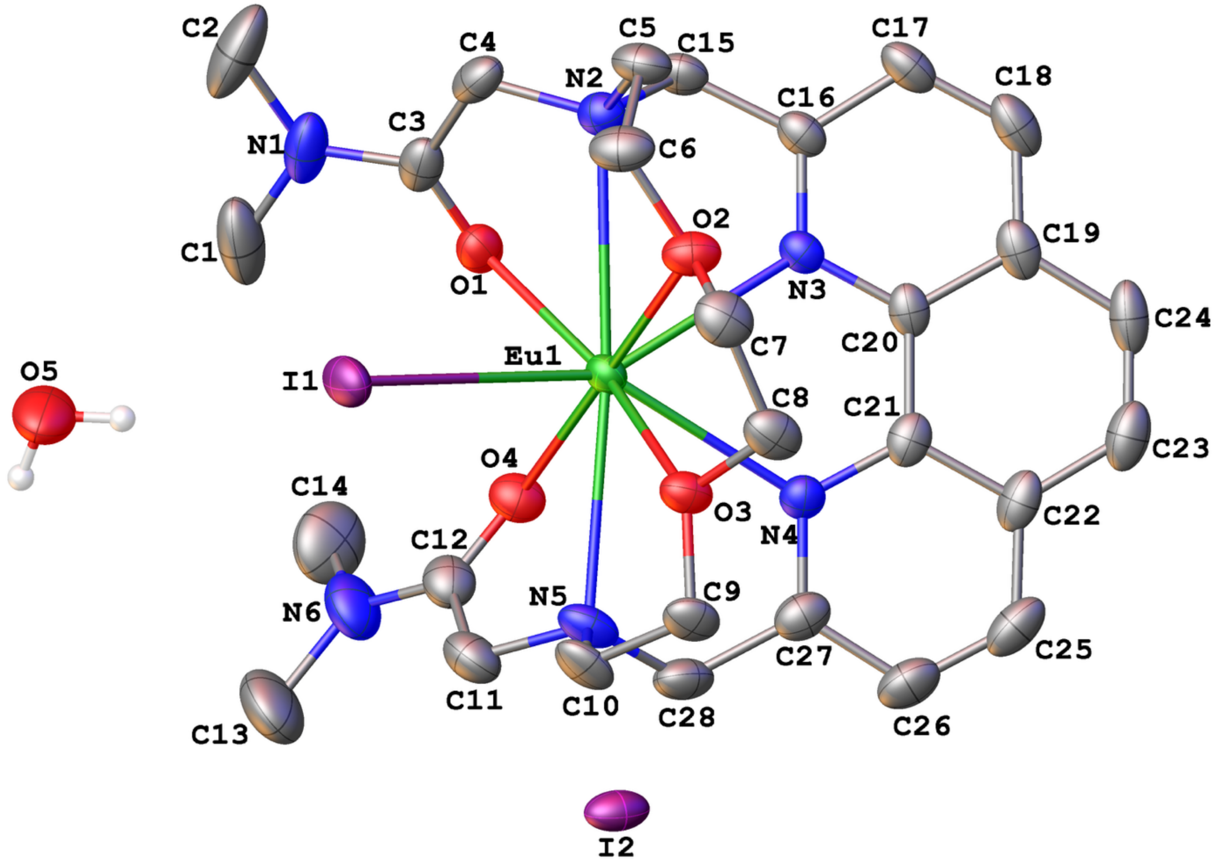

Figure S101 – Molecular drawing of [Eu(**PhenDMA**)I]I shown with 50% probability ellipsoids. All H atoms bonded to C atoms and the minor components of disorder are omitted. Atom color: gray = carbon, blue = nitrogen, red = oxygen, purple = iodide, white = hydrogen, green = europium.

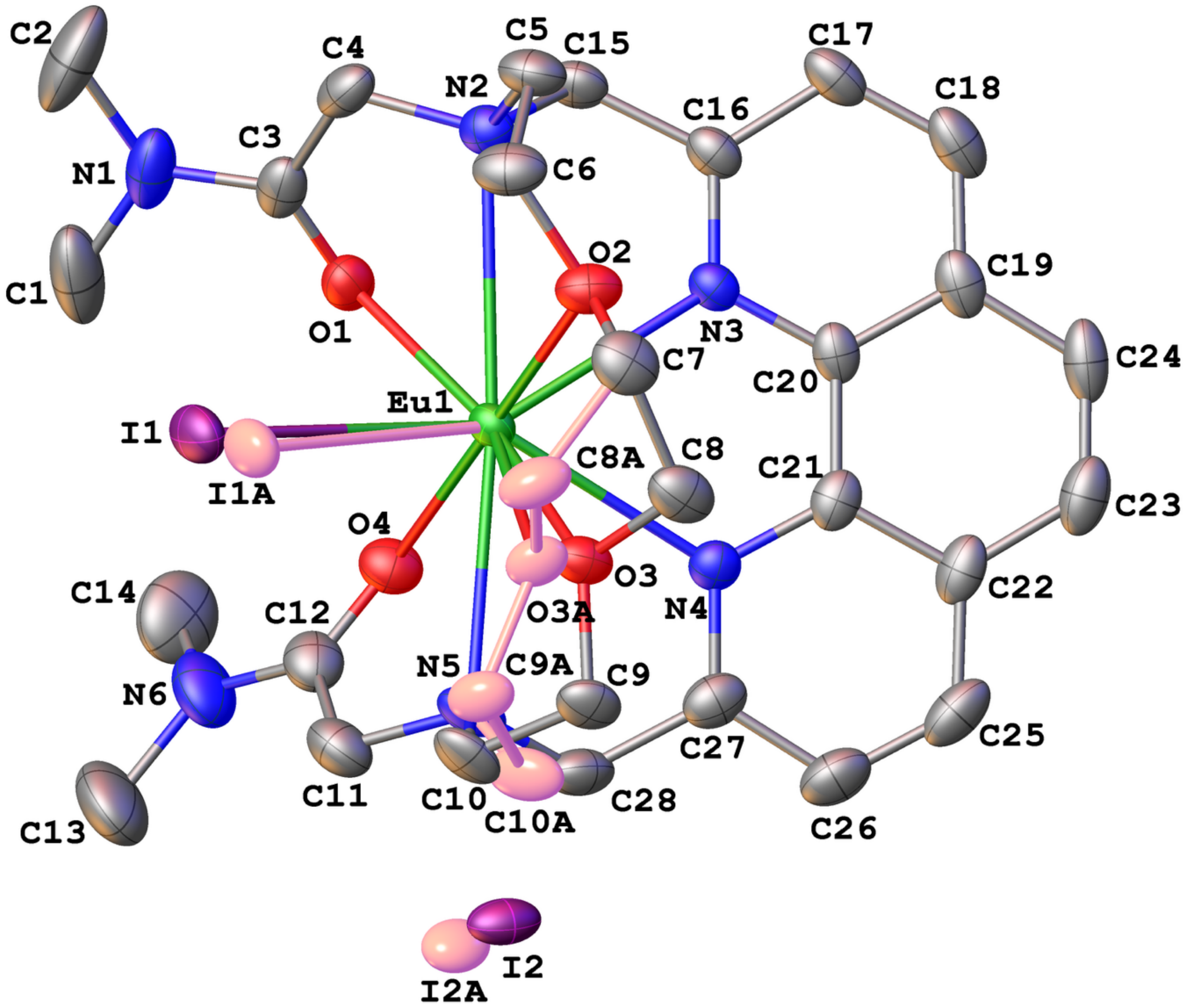

Figure S102 – Molecular drawing of the ionic pair in [Eu(**PhenDMA**)I]I highlighting the disordered atoms. Minor components of the positional disorder are drawn in pink. Shown with 50% probability ellipsoids, and all H atoms and the partially occupied water molecule are omitted. Atom color: gray = carbon, blue = nitrogen, red = oxygen, purple = iodide, green = europium.

Table S6 – Crystal data and structure refinement for [Eu(PhenDMA)I]I.

Empirical formula	$[Eu(C_{28}H_{38}N_6O_4)I]I\cdot0.844H_2O$
Formula weight	943.62
Temperature/K	100.00
Crystal system	monoclinic
Space group	$P2_1/c$
a/Å	11.293(2)
b/Å	18.739(4)
c/Å	16.492(4)
α/°	90
β/°	109.227(9)
γ/°	90
Volume/Å^3	3295.4(13)
Z	4
ρ_{calc}g/cm^3	1.902
μ/mm^{-1}	3.821
F(000)	1,830.0
Crystal size/mm^3	0.06 × 0.03 × 0.01
Radiation	Mo Kα (λ = 0.71073)
2Θ range for data collection/°	3.82 to 56.646
Index ranges	–15 ≤ h ≤ 14, –25 ≤ k ≤ 24, –21 ≤ l ≤ 21
Reflections collected	92,355
Independent reflections	8,190 [R_{int} = 0.0453, R_{sigma} = 0.0405]
Data/restraints/parameters	8,190/43/438
Goodness-of-fit on F^2	1.050
Final R indexes [I ≥ 2σ (I)]	R_1 = 0.0318, wR_2 = 0.0673
Final R indexes [all data]	R_1 = 0.0494, wR_2 = 0.0710
Largest diff. peak/hole / e Å^{-3}	1.35/–0.88

11.4 Crystallographic Information for $[Eu(Phencrypt)(H_2O)_2]I_2$

Data Collection

A red crystal with approximate dimensions 0.112 mm × 0.071 mm × 0.048 mm was selected under oil under ambient conditions and attached to the tip of a MiTeGen MicroMount©. The crystal was mounted in a stream of cold nitrogen at 100(1) K and centered in the X-ray beam by using a video camera.

Crystal evaluation and data collection were performed on a Bruker D8 VENTURE Photon III four-circle diffractometer with Diamond II IµS Mo Kα (λ = 0.71073 Å) radiation and the detector to crystal distance of 5.0 cm.[16]

Initial cell constants were obtained from a 180° φ scan conducted at a 2θ = 30° angle with an exposure time of 1 s per frame. Reflections were successfully indexed using an automated indexing routine built in the APEX6 program. Final cell constants were calculated from a set of 9,205 strong reflections from the actual data collection.

Data were collected by using the half-sphere data collection routine to survey the reciprocal space to the extent of a half sphere to a resolution of 0.70 Å. A total of 145,436 data were harvested by collecting 11 sets of frames with 0.5º scans in ω and φ with an exposure time of 1–12 s per frame. These highly redundant datasets were corrected for Lorentz and polarization effects. The absorption correction was based on fitting a function to the empirical transmission surface as sampled by multiple equivalent measurements.[17]

Structure Solution and Refinement

Systematic absences in the diffraction data were uniquely consistent for the space group $P2_1/n$ that yielded chemically reasonable and computationally stable results of refinement.[18–23]

A successful solution by intrinsic phasing provided most non-hydrogen atoms from the *E*-map. The remaining non-hydrogen atoms were in an alternating series of least-squares cycles and difference Fourier maps. All non-hydrogen atoms were refined with anisotropic atomic displacement coefficients. The hydrogen atoms bonded to O atoms were refined with distance restraints and relative isotropic atomic displacement parameters. All other hydrogen atoms were included in the structure factor calculation at idealized positions and were allowed to ride on the neighboring atoms with relative isotropic atomic displacement coefficients.

The asymmetric unit contains the $[EuC_{26}H_{38}N_4O_4]I_2$ compound. There are also unidentified decomposition products, side products, or solvent molecules present in the asymmetric unit. The Eu^{2+} oxidation state was independently verified using EPR spectroscopy. In addition, the red color of the crystals is indicative of Eu^{2+}.

The unidentified species present in the asymmetric unit cannot be modeled with a chemically reasonable representation. These molecules are partially occupied and are likely disordered over multiple positions. A significant amount of time was invested in identifying and refining the disordered molecules. The OLEX2.Mask was used to correct the diffraction data for diffuse scattering effects. OLEX2 calculated the upper limit of volume that can be occupied by the solvent to be 376 Å^3, or 11.5% of the unit cell volume. The program calculated 64 electrons in the unit cell for the diffuse species. Please note that all derived results in the following tables are based on the known contents. No data are given for the diffusely scattering species.

The final least-squares refinement of 364 parameters against 9,981 data resulted in residuals R (based on F^2 for $I \geq 2\sigma$) and wR (based on F^2 for all data) of 0.0253 and 0.0700, respectively. The final difference Fourier map contains several peaks of residual electron density (approximately 2.74 e$^-$/Å^3) in the structure. These peaks are in chemically unreasonable positions around the heavy Eu and I atoms and were considered noise.

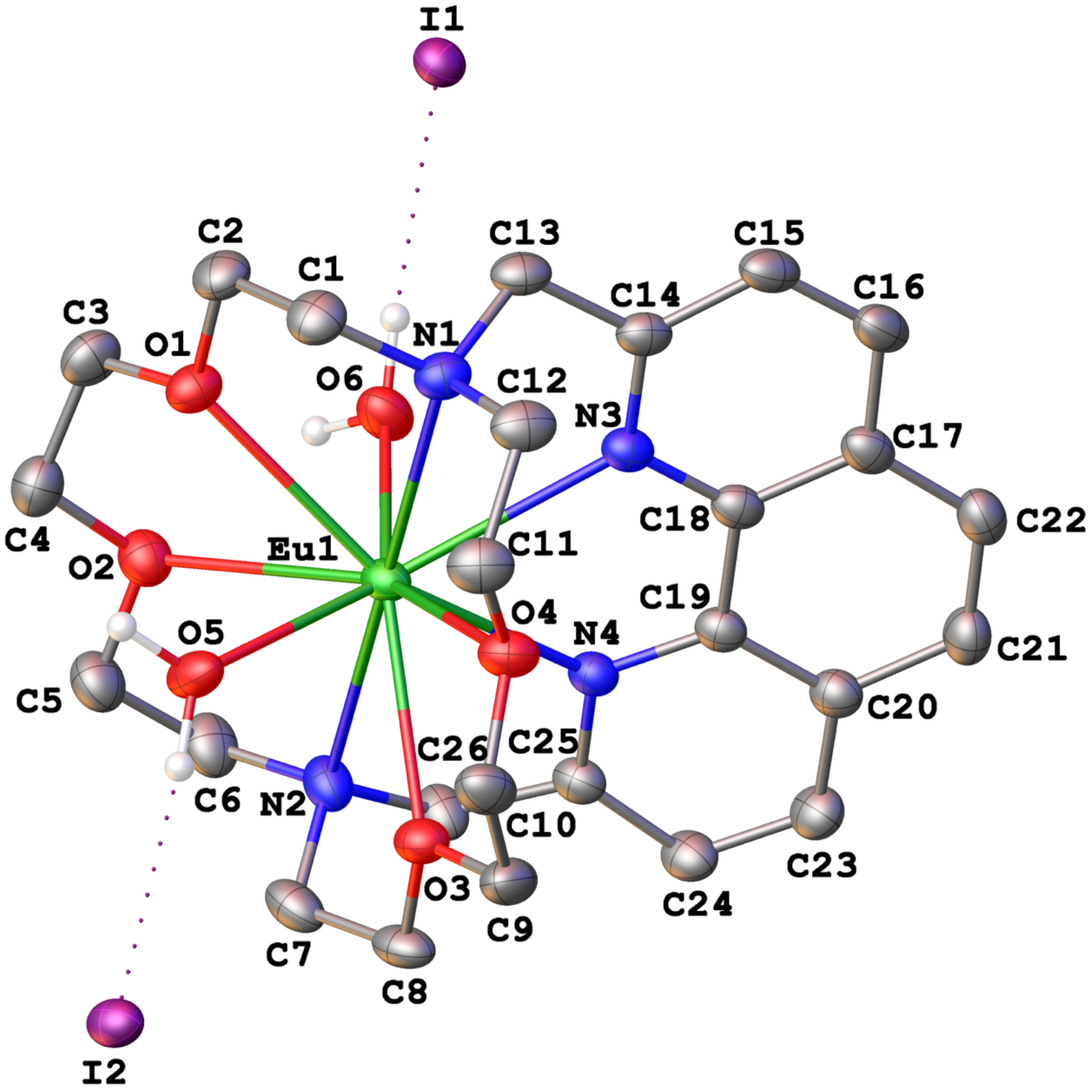

Figure S103 – Molecular drawing of [Eu(**Phencrypt**)(H_2O)$_2$]I_2 shown with 50% probability ellipsoids. All H atoms bonded to C atoms are omitted. Atom color: gray = carbon, blue = nitrogen, red = oxygen, purple = iodide, white = hydrogen, green = europium.

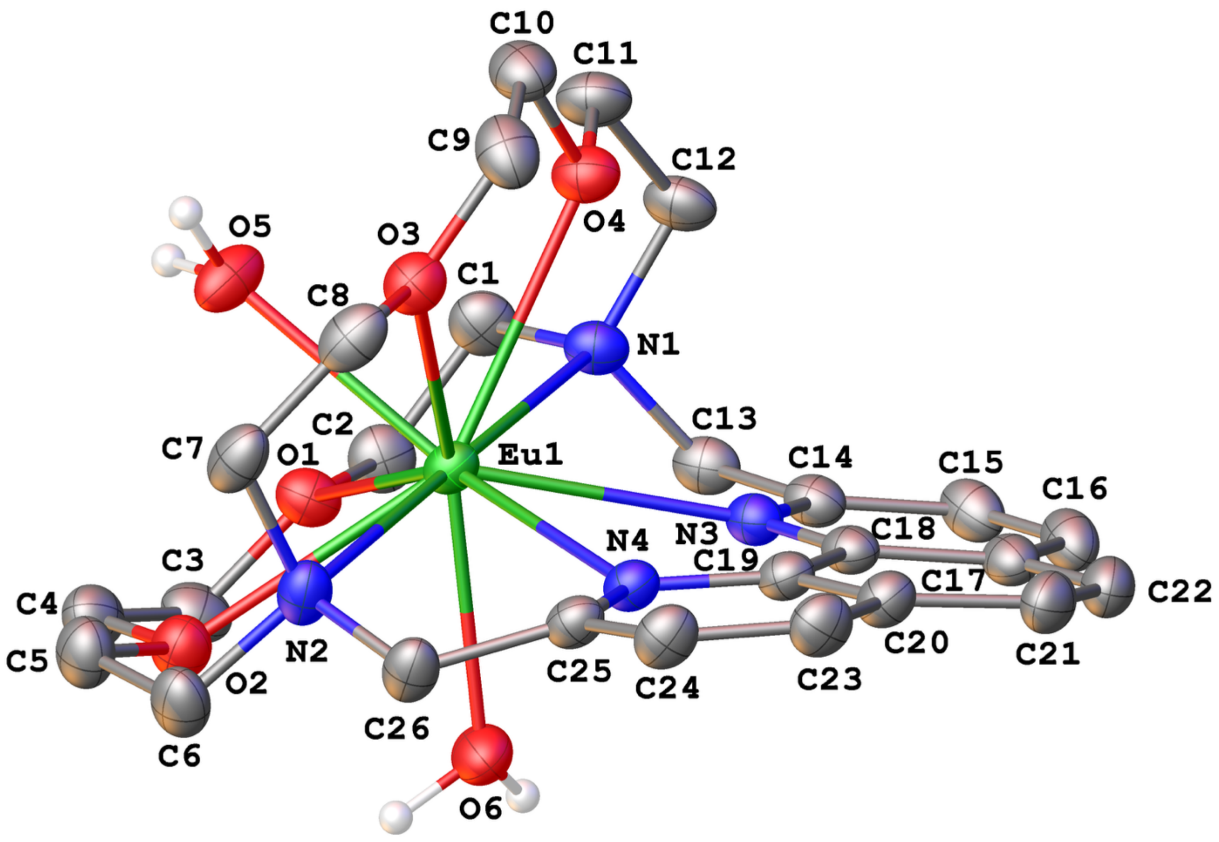

Figure S104 – Molecular drawing of the $[EuC_{26}H_{38}N_4O_4]^{2+}$ di-cation in [Eu(**Phencrypt**)$(H_2O)_2]I_2$. Shown with 50% probability ellipsoids and all H atoms bonded to C atoms are omitted. Atom color: gray = carbon, blue = nitrogen, red = oxygen, purple = iodide, white = hydrogen, green = europium.

Table S7 – Crystal data and structure refinement for [Eu(Phencrypt)(H_2O)$_2$]I_2.

Empirical formula	[$EuC_{26}H_{38}N_4O_4$]I_2
Formula weight	908.36
Temperature/K	100.00
Crystal system	monoclinic
Space group	$P2_1/n$
a/Å	17.8043(19)
b/Å	9.1627(14)
c/Å	21.866(3)
α/°	90
β/°	113.566(8)
γ/°	90
Volume/Å^3	3,269.7(8)
Z	4
ρ_{calc}g/cm^3	1.845
μ/mm^{-1}	3.848
F(000)	1,756.0
Crystal size/mm^3	0.112 × 0.071 × 0.048
Radiation	Mo Kα (λ = 0.71073)
2Θ range for data collection/°	3.796 to 61.066
Index ranges	$-25 \leq h \leq 23$, $-13 \leq k \leq 13$, $-31 \leq l \leq 31$
Reflections collected	141,014
Independent reflections	9981 [R_{int} = 0.0380, R_{sigma} = 0.0163]
Data/restraints/parameters	9,981/6/364
Goodness-of-fit on F^2	1.057
Final R indexes [I ≥ 2σ (I)]	R_1 = 0.0253, wR_2 = 0.0675
Final R indexes [all data]	R_1 = 0.0281, wR_2 = 0.0700
Largest diff. peak/hole / e Å^{-3}	2.74/–0.80

11.5 Crystallographic Information for [Eu(Phencrypt)Br]Br_2

Data Collection

A colorless crystal with approximate dimensions 0.14 mm × 0.10 mm × 0.04 mm was selected under oil under ambient conditions and attached to the tip of a MiTeGen MicroMount©. The crystal was mounted in a stream of cold nitrogen at 100(1) K and centered in the X-ray beam by using a video camera.

Crystal evaluation and data collection were performed on a Bruker D8 VENTURE Photon III four-circle diffractometer with Diamond II IμS Mo Kα (λ = 0.71073 Å) radiation and the detector to crystal distance of 7.5 cm.[16]

Initial cell constants were obtained from a 180° φ scan conducted at a 2θ = 30° angle with an exposure time of 1 s per frame. Reflections were indexed by an automated indexing routine built in the APEX6 program. Final cell constants were calculated from a set of 9,944 strong reflections from the actual data collection.

Data were collected using the full-sphere data collection routine to survey the reciprocal space to the extent of a full sphere to a resolution of 0.75 Å. A total of 79,289 data were harvested by collecting 11 sets of frames with 0.5–1.0º scans in ω and φ with an exposure time of 1–7 s per frame. These highly redundant datasets were corrected for Lorentz and polarization effects. The absorption correction was based on fitting a function to the empirical transmission surface as sampled by multiple equivalent measurements.[17]

Structure Solution and Refinement

The systematic absences in the diffraction data were uniquely consistent for the space group *Fddd* that yielded chemically reasonable and computationally stable results of refinement.[18,20–23]

A successful solution by intrinsic phasing provided most non-hydrogen atoms from the *E*-map. The remaining non-hydrogen atoms were in an alternating series of least-squares cycles and difference Fourier maps. All non-hydrogen atoms were refined with anisotropic atomic displacement coefficients unless otherwise noted. All hydrogen atoms were included in the structure factor calculation at idealized positions and were allowed to ride on the neighboring atoms with relative isotropic atomic displacement coefficients.

The overall molecular formula for this compound is $[Eu(C_{26}H_{34}N_4O_4)Br]Br_2{\cdot}1.5C_4H_8O$. Only one-half of the molecular formula is present in the asymmetric unit. The $[Eu(C_{26}H_{34}N_4O_4)Br]^{2+}$ dication and the C_4H_8O solvent molecules reside on special positions and are positionally disordered.

The Eu1 atom resides on a crystallographic 2-fold rotation axis, thus, only one-half of the $[Eu(C_{26}H_{34}N_4O_4)Br]^{2+}$ dication is symmetry independent. The $C_{26}H_{34}N_4O_4$ ligand coordinated to the Eu1 cation contains one fragment with positional disorder. The O1, O2, N1, and C1—C6 atoms

are disordered over two positions with a dominant component occupation of 79.1(12)%. The disordered components are refined with restrained geometries and restrained anisotropic atomic displacement coefficients. The Br1 atom coordinated to the Eu1 atom is equally disordered about the 2-fold rotation axis.

The O3 C_4H_8O molecule resides on a crystallographic inversion center; therefore, only one-half of the molecule is symmetry independent. In addition, the O3 C_4H_8O molecule is disordered over two positions with an occupation ratio of 28.5(9)/21.5(9)%. The disordered components are refined with restrained geometries and restrained anisotropic atomic displacement coefficients. Each of the disordered components of the molecule have a symmetry-related component resulting in four total positions for the O3 C_4H_8O molecule.

The O4 C_4H_8O molecule resides on the intersection of three 2-fold crystallographic axes, thus, only one-quarter of the molecule is present in the asymmetric unit. The O4 C_4H_8O molecule is also positionally disordered with an occupation ration of 16.5(8)/8.5(8)%. The disordered components are refined with geometric restraints and isotropic atomic displacement parameter restraints. Each of the disordered components have three symmetry-related positions, resulting in eight total positions for the O4 C_4H_8O molecule.

The final least-squares refinement of 391 parameters against 4,454 data resulted in residuals R (based on F^2 for $I \geq 2\sigma$) and wR (based on F^2 for all data) of 0.0351 and 0.0912, respectively. The final difference Fourier map contains residual peaks of electron density of 1.71 e/Å^3 near the Eu and Br atoms. These peaks are in chemically unreasonable positions and are therefore considered noise.

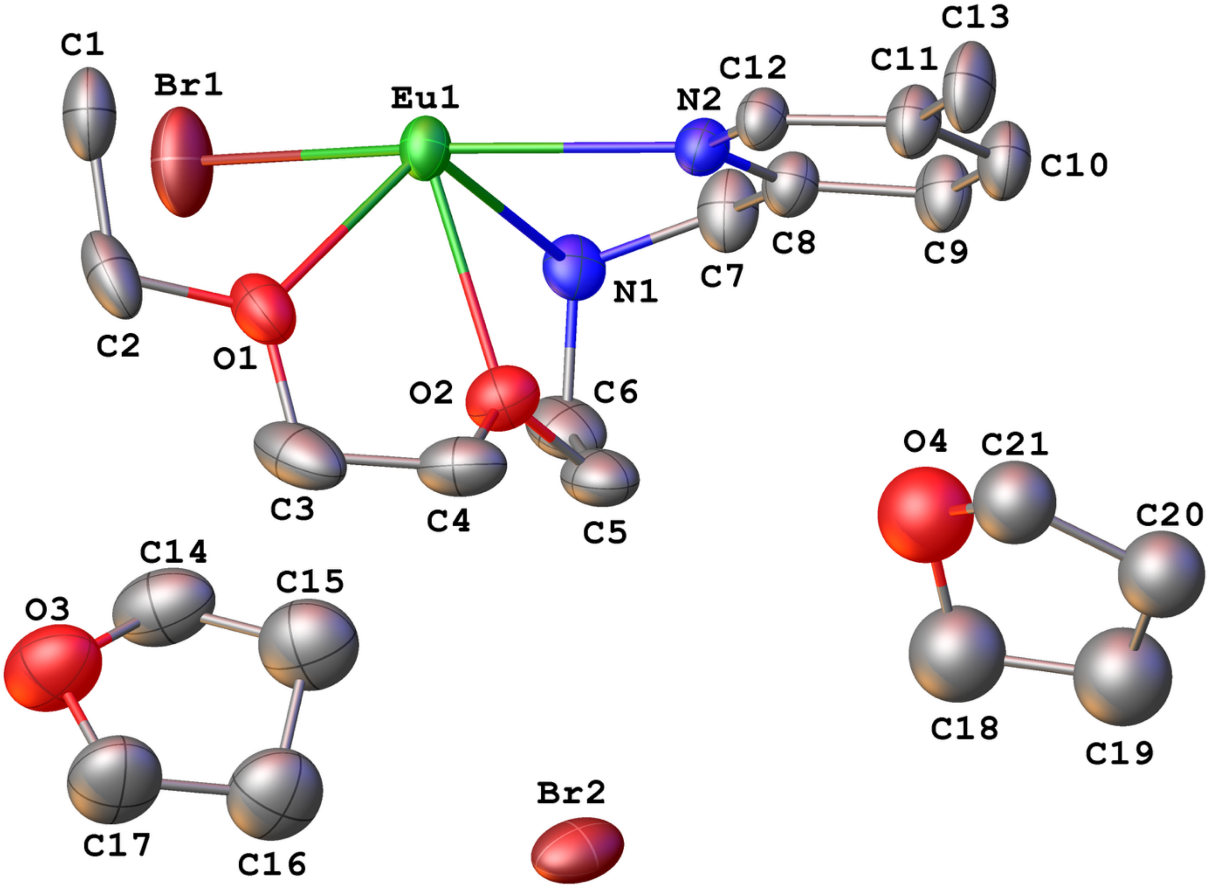

Figure S105 – Drawing of the asymmetric unit in [Eu(**Phencrypt**)Br]Br_2 shown with 50% probability ellipsoids. In this representation, the Eu1 and Br1 atoms are half occupied, the major component of the O3 C_4H_8O molecule is 28.5(9)% occupied, and the major component of the O4 C_4H_8O molecule is 16.5(8)% occupied. All H atoms and the minor components of disorder are omitted. Atom color: gray = carbon, blue = nitrogen, red = oxygen, dark red = bromide, green = europium.

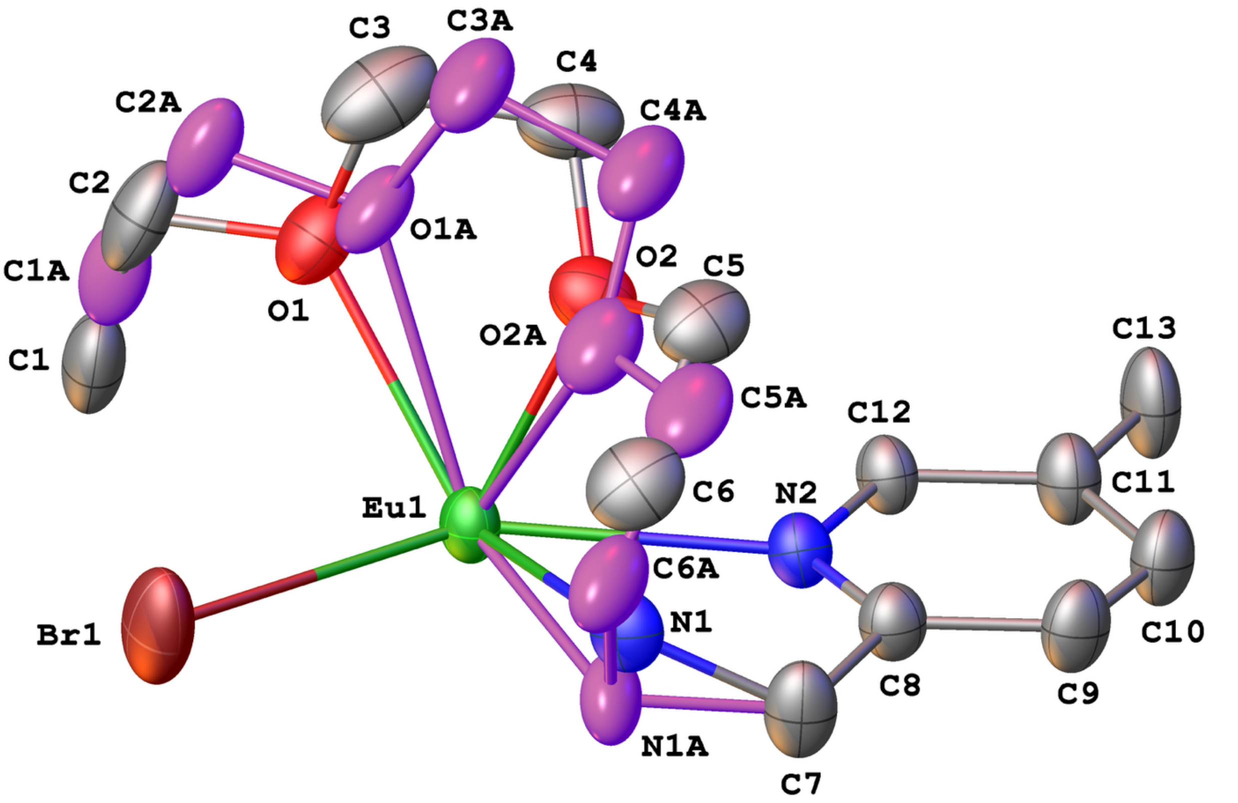

Figure S106 – Molecular drawing highlighting the positional disorder in the $[Eu(C_{26}H_{34}N_4O_4)Br]^{2+}$ dication in [Eu(**Phencrypt**)Br]Br_2. The Eu1 and Br1 atoms are half occupied in this representation. The minor components of the positional disorder are drawn in purple. This diagram is shown with 50% probability ellipsoids, and all H atoms are omitted. Atom color: gray = carbon, blue = nitrogen, red = oxygen, dark red = bromide, green = europium.

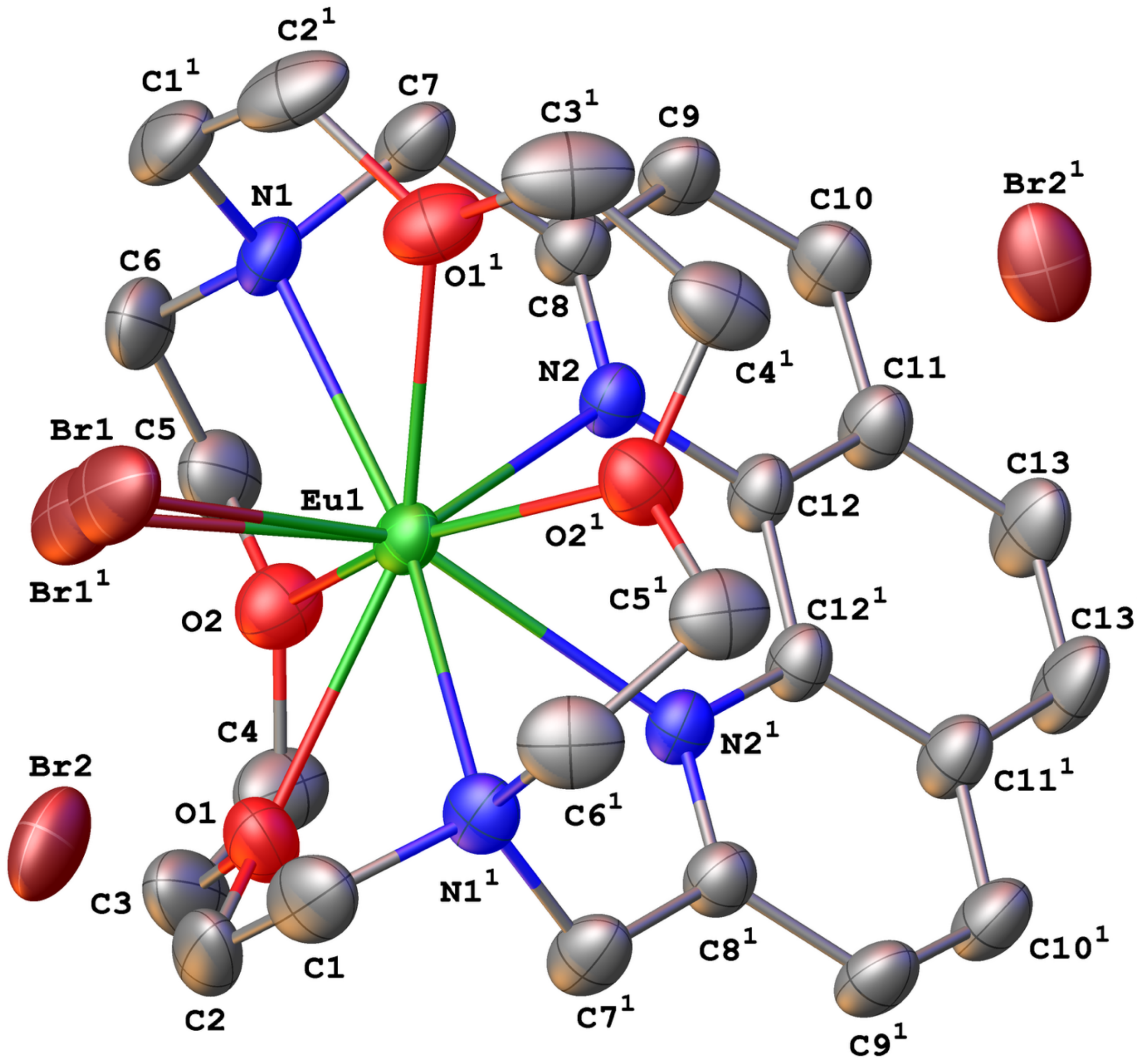

Figure S107 – A molecular drawing of the completed $[Eu(C_{26}H_{34}N_4O_4)Br]Br_2$ ionic composition in[Eu(**Phencrypt**)Br]Br_2. The Br1 atoms are each half occupied. The figure is drawn with 50% probability ellipsoids, and all H atoms and the minor components of disorder are omitted. Symmetry code: (1) 5/4-X, 5/4-Y, Z. Atom color: gray = carbon, blue = nitrogen, red = oxygen, dark red = bromide, green = europium.

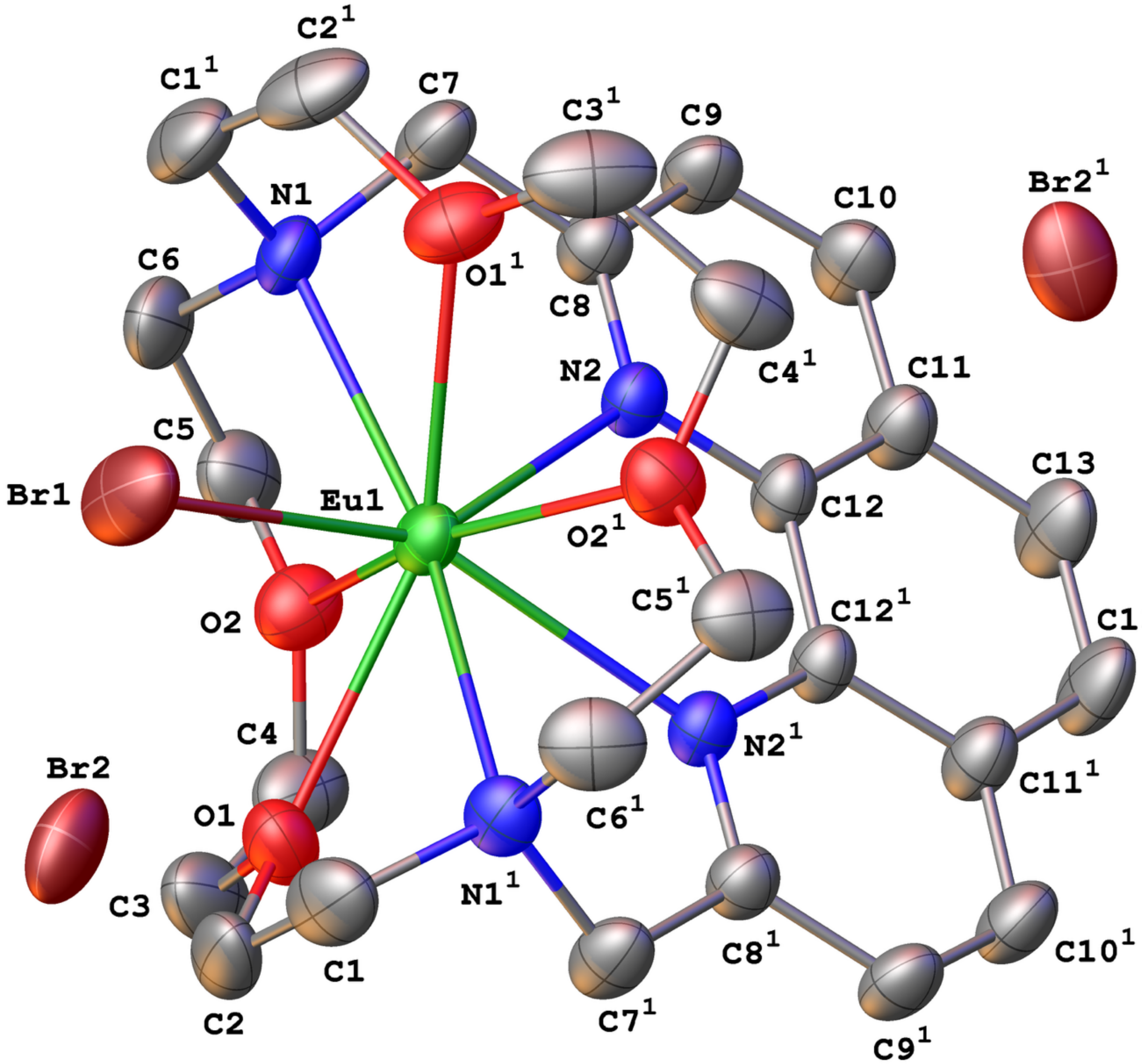

Figure S108 – Molecular drawing of the completed [Eu($C_{26}H_{34}N_4O_4$)Br]Br_2 ionic composition in [Eu(**Phencrypt**)Br]Br_2. The figure is drawn with 50% probability ellipsoids. The second position for the Br1 atom, the minor component of the positional disorder, and all H atoms are omitted. Symmetry code: (1) 5/4–X, 5/4–Y, Z. Atom color: gray = carbon, blue = nitrogen, red = oxygen, dark red = bromide, green = europium.

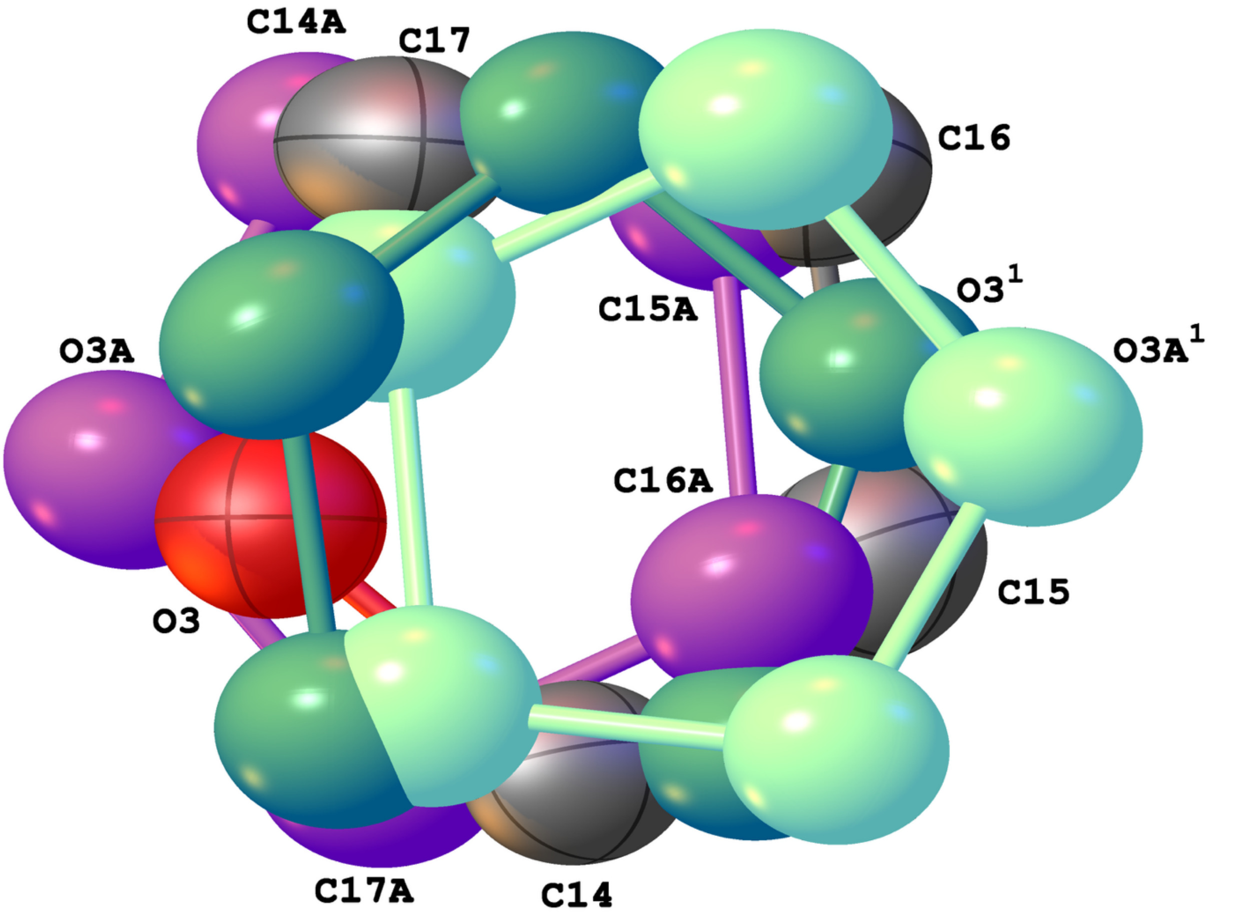

Figure S109 – Molecular drawing of the four positionally disordered and symmetry-related O3 C_4H_8O molecules. The molecules are drawn with 50% probability ellipsoids, and all H atoms are omitted. Symmetry code: (1) 1/2–X, 3/2–Y, 1–Z. Atom color: gray = carbon, red = oxygen.

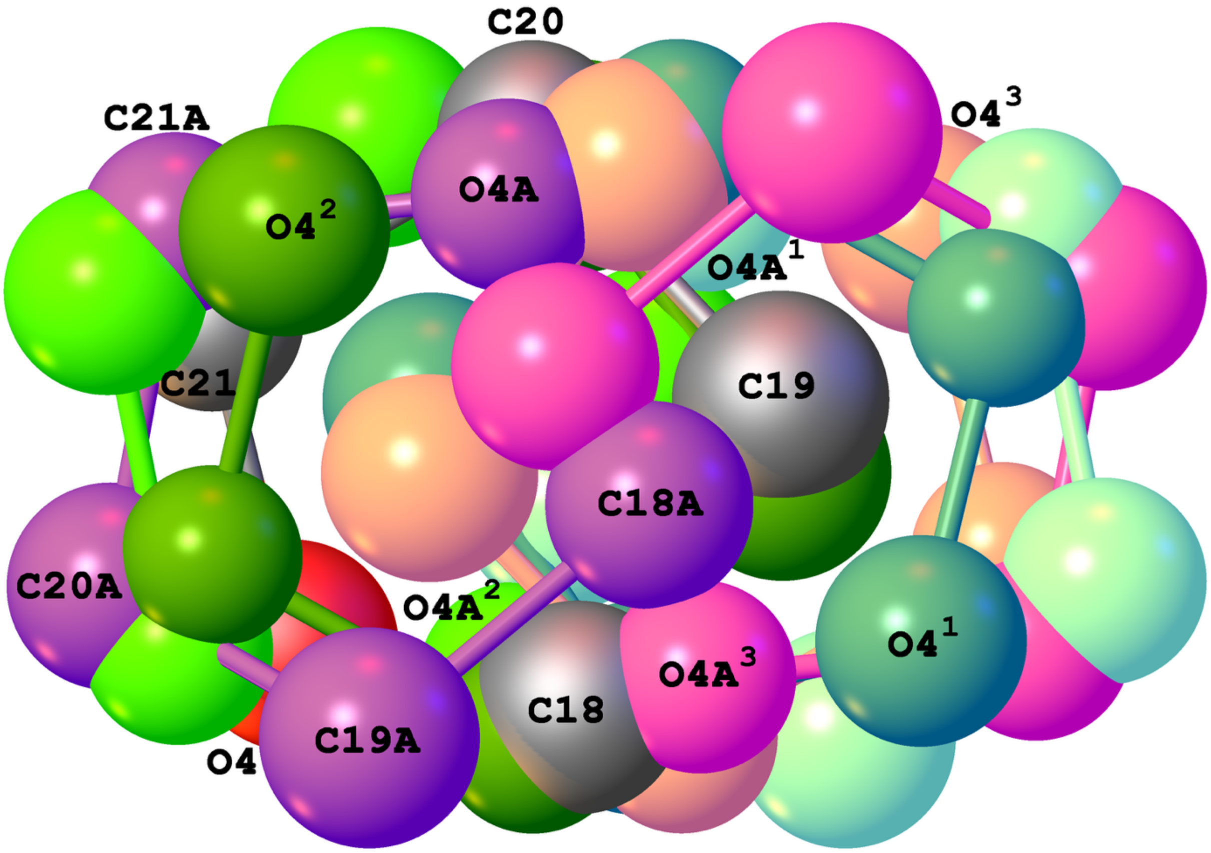

Figure S110 – Molecular drawing of the eight positions for the disordered and symmetry-related O4 C_4H_8O molecules. The molecules are drawn with 50% probability ellipsoids, and all H atoms are omitted. Symmetry code: (1) 3/4–X, 7/4–Y, Z; (2) 3/4–X, Y, 3/4–Z; (3) X, 7/4–Y, 3/4–Z. Atom color: gray = carbon, red = oxygen.

Table S8 – Crystal data and structure refinement for [Eu(Phencrypt)Br]Br_2.

Empirical formula	[Eu($C_{26}H_{34}N_4O_4$)Br]Br_2·1.5C_4H_8O
Formula weight	966.42
Temperature/K	100.00
Crystal system	orthorhombic
Space group	*Fddd*
a/Å	10.797(3)
b/Å	19.970(6)
c/Å	66.34(2)
α/°	90
β/°	90
γ/°	90
Volume/Å^3	14,305(8)
Z	16
ρ_{calc}g/cm^3	1.795
μ/mm^{-1}	5.151
F(000)	7,648.0
Crystal size/mm^3	0.14 × 0.1 × 0.04
Radiation	Mo Kα (λ = 0.71073)
2Θ range for data collection/°	4.26 to 56.586
Index ranges	$-14 \le h \le 14$, $-26 \le k \le 26$, $-88 \le l \le 88$
Reflections collected	75,470
Independent reflections	4,454 [R_{int} = 0.0438, R_{sigma} = 0.0154]
Data/restraints/parameters	4,454/410/391
Goodness-of-fit on F^2	1.074
Final R indexes [I ≥ 2σ (I)]	R_1 = 0.0351, wR_2 = 0.0883
Final R indexes [all data]	R_1 = 0.0403, wR_2 = 0.0912
Largest diff. peak/hole / e Å^{-3}	1.71/–1.40

12. LC–MS Data

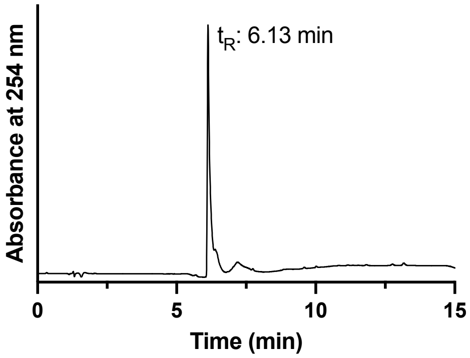

Figure S111 – LC–MS trace of **PhenDMA** (**Method A**).

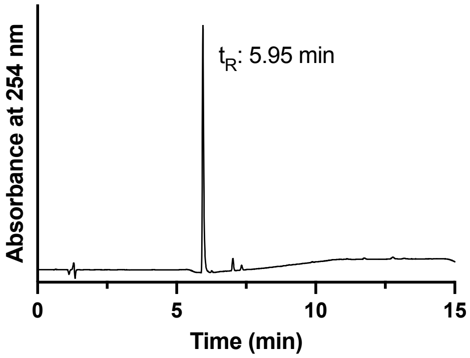

Figure S112 – LC–MS trace of **BipyDMA** (**Method A**).

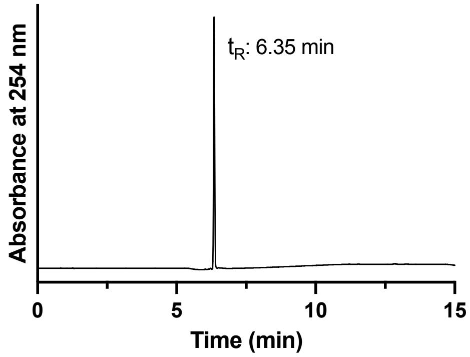

Figure S113 – LC–MS trace of **Phenacetate** (**Method A**).

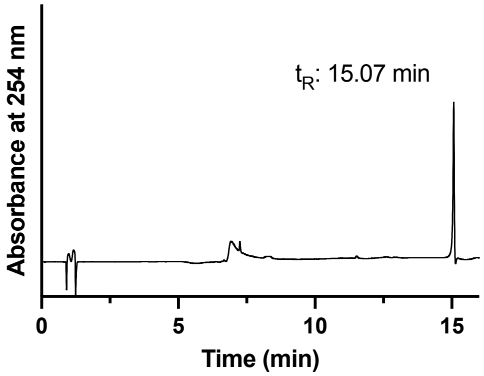

Figure S114 – LC-MS trace of [Eu(**Phen18c6**)]$^{3+}$ (**Method B)**.

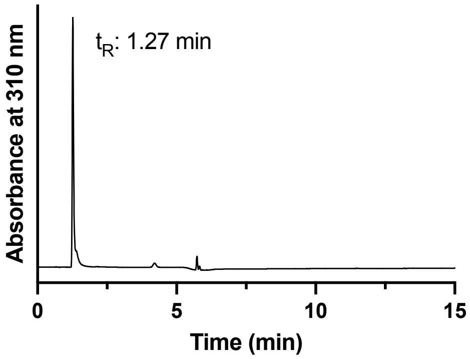

Figure S115 – LC-MS trace of [Eu(**BipyDMA**)]$^{3+}$(**Method A**).

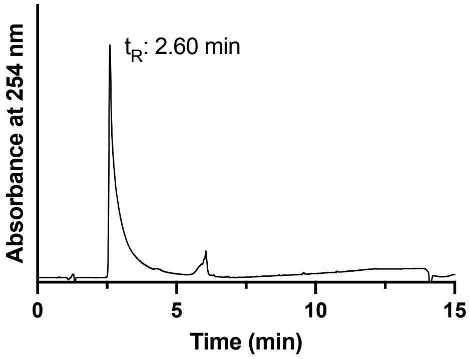

Figure S116 – LC–MS trace of [Eu(**PhenDMA**)]$^{3+}$ (**Method C**).

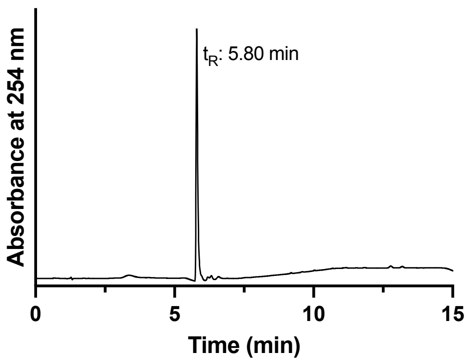

Figure S117 – LC–MS traces of [Eu(**Phenacetate**)]$^{+}$ (**Method A**).

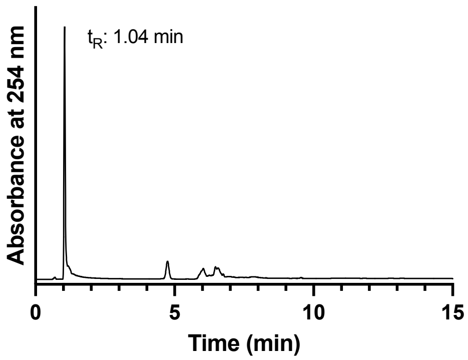

Figure S118 – LC–MS trace of [Eu(**Phencrypt**)]$^{3+}$ (**Method A**).

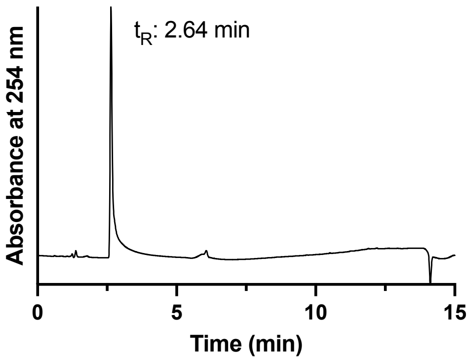

Figure S119 – LC–MS trace of [Gd(**PhenDMA**)]$^{3+}$ (**Method C**).

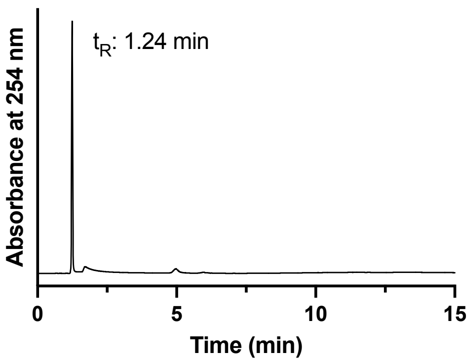

Figure S120 – LC-MS trace of [Gd(**BipyDMA**)]$^{3+}$ (**Method A**).

13. ESI–HRMS Data

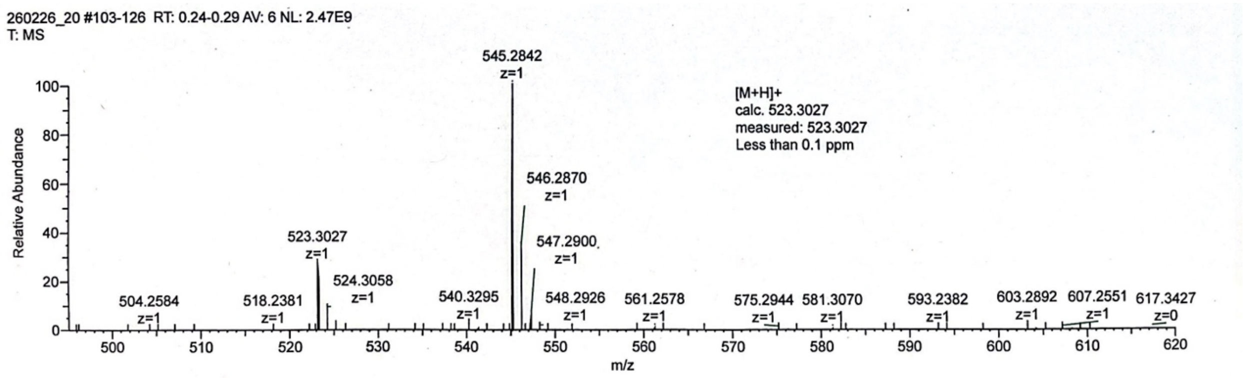

Figure S121 –ESI–HRMS of **PhenDMA** – [M+H]$^+$: calc. 523.3027, found: 523.3027 [M+Na]$^+$ calc. 545.2852, found: 545.2842.

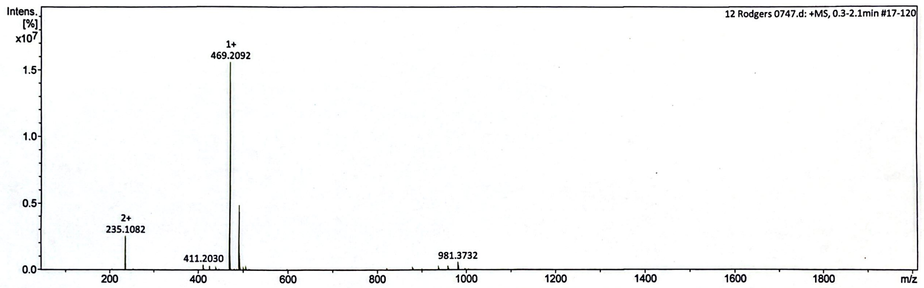

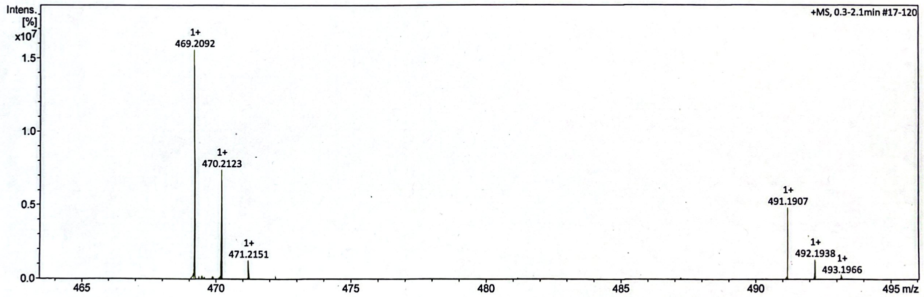

Figure S122 –ESI–HRMS of **Phenacetate** – $[M+H]^+$: calc. 469.2082, found: 469.2092.

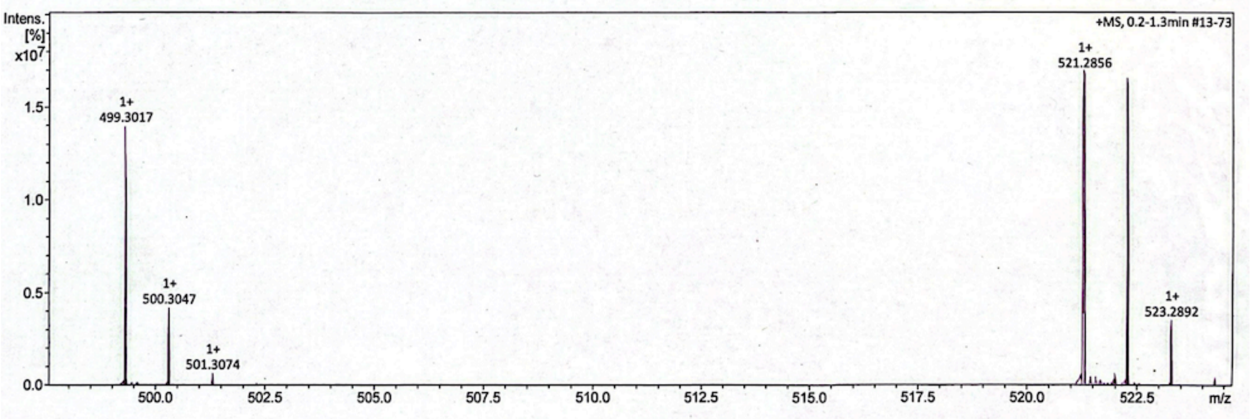

Figure S123 –ESI–HRMS of **BipyDMA** –$[M+H]^+$ = calc. 499.3027 , found: 499.3017 $[M+H]^+$; $[M+Na]^+$ = calc. 521.2847, found: 521.2856.

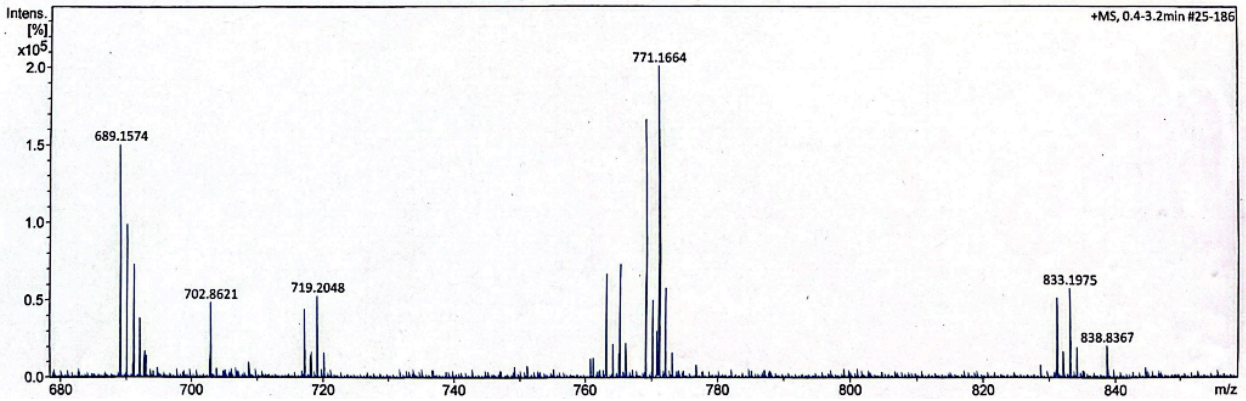

Figure S124 –ESI–HRMS of [Eu(**PhenDMA**)]$^{3+}$ – [EuL · 2Br]$^{+}$ = calc. 833.0533, found: 833.1975.

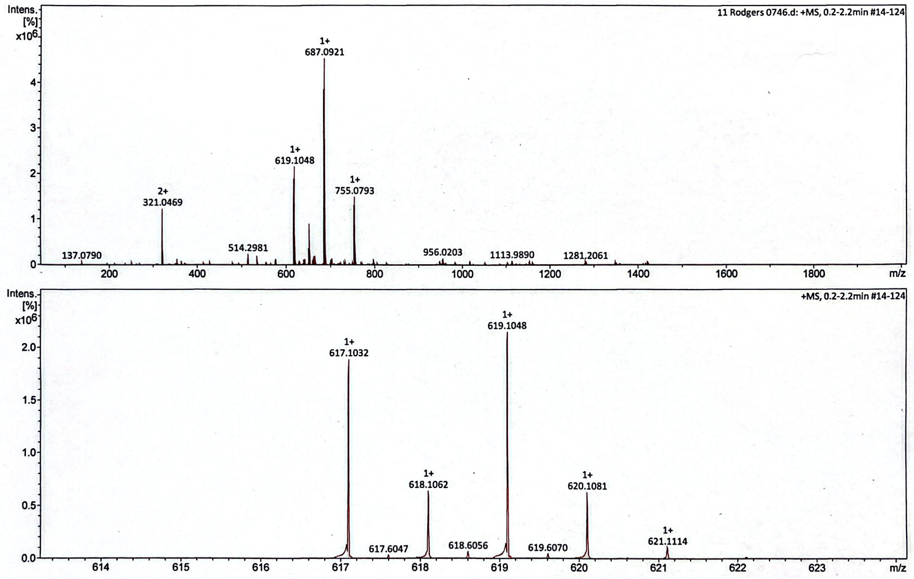

Figure S125 –ESI–HRMS of [Eu(**Phenacetate**)]$^{+}$ – [Eu · L]$^{+}$ = calc. 617.1045, found: 617.1032.

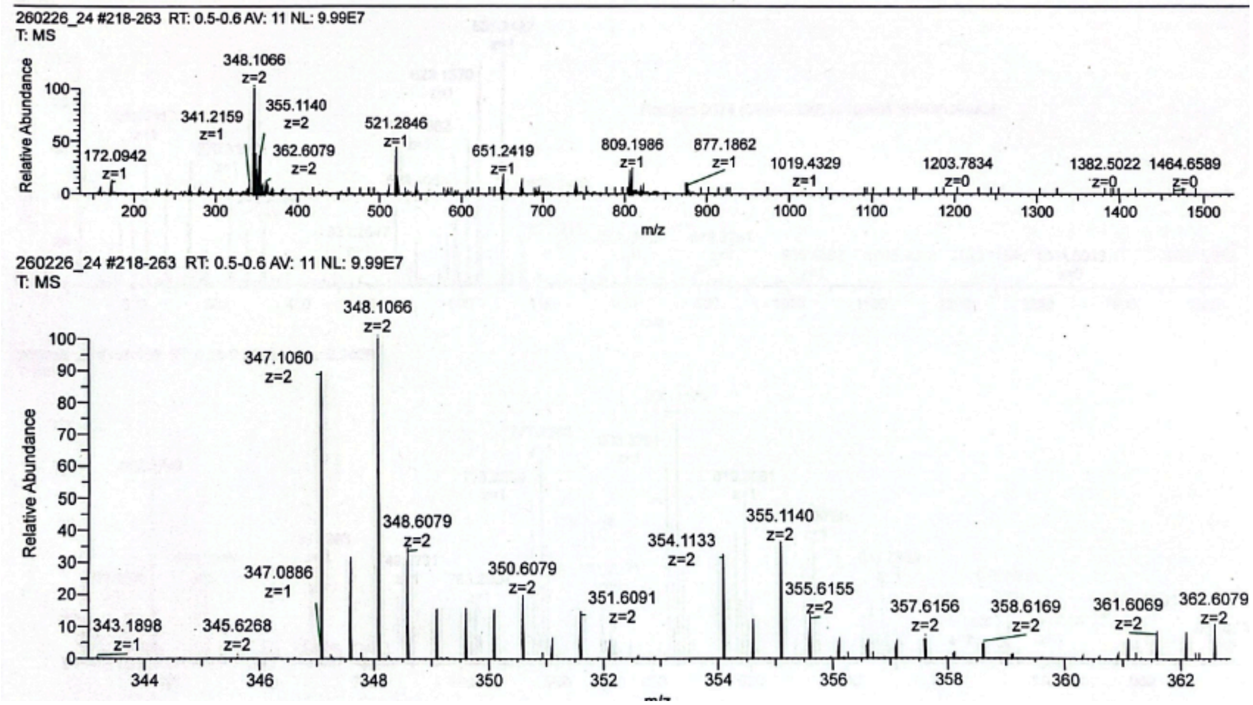

Figure S126 –ESI–HRMS of [Eu(**BipyDMA**)]$^{3+}$ – [EuL · (CHO_2]$^{2+}$ = calc. 348.1072, found: 348.1072.

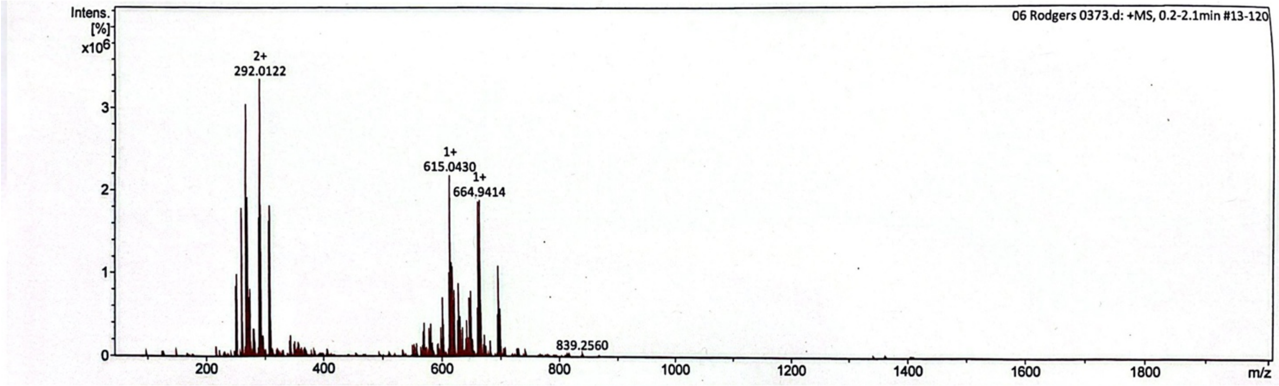

Figure S127 – ESI–HRMS of [Eu(**Phen18c6**)]$^{3+}$; [EuL · 2Br]$^{+}$ = calc. 664.9481, found: 664.9414.

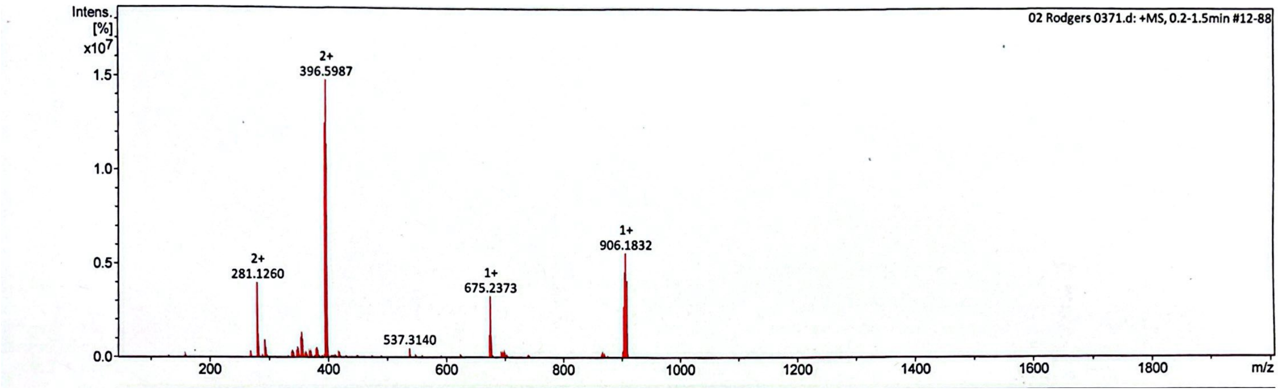

Figure S128 – ESI–HRMS of Gd(**PhenDMA**)$^{3+}$; [GdL · 2($C_2O_2F_3$)]$^{+}$ = calc. 906.1896, found: 906.1832

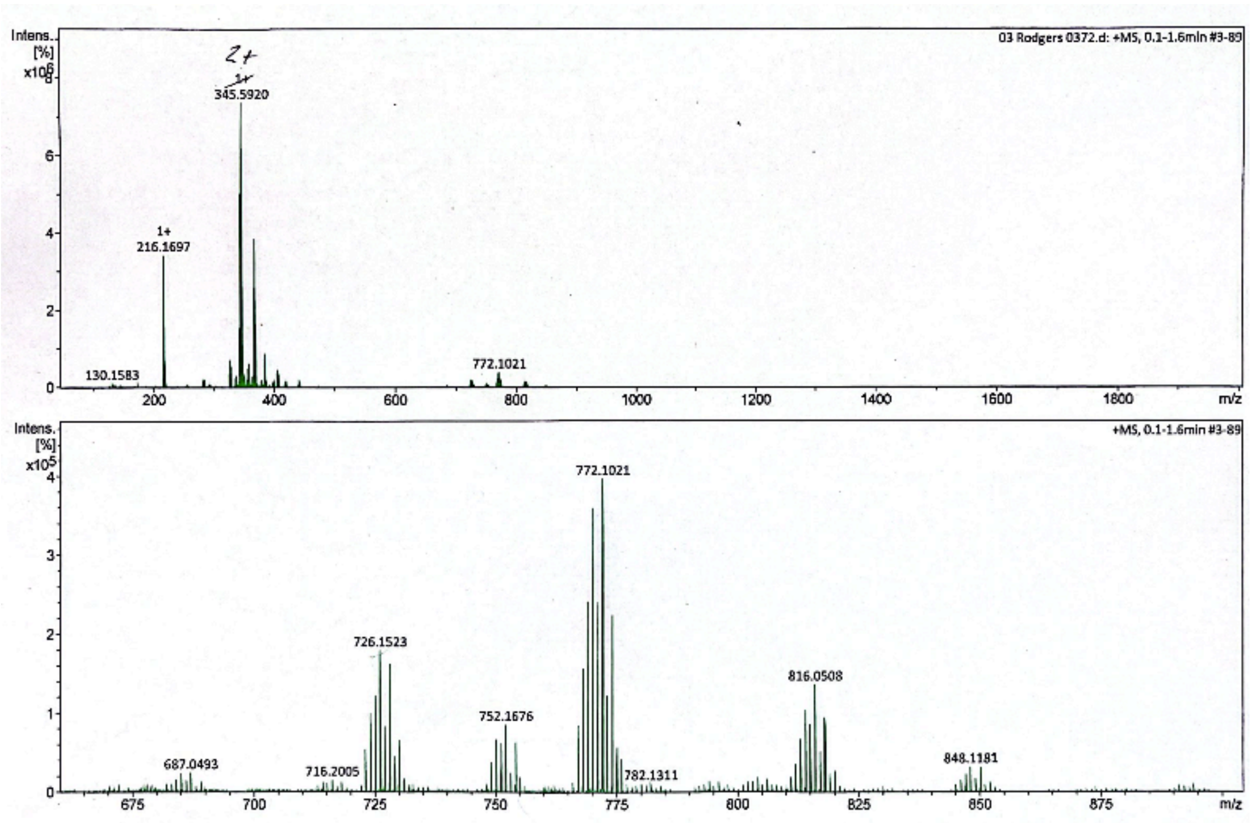

Figure S129 – ESI–HRMS of Gd(**BipyDMA**)$^{3+}$; [GdL · 2Cl]$^{+}$ = calc. 726.1573, found. 726.1523; [GdL· Cl]$^{2+}$ = calc. 345.5942, found. 345.5920.

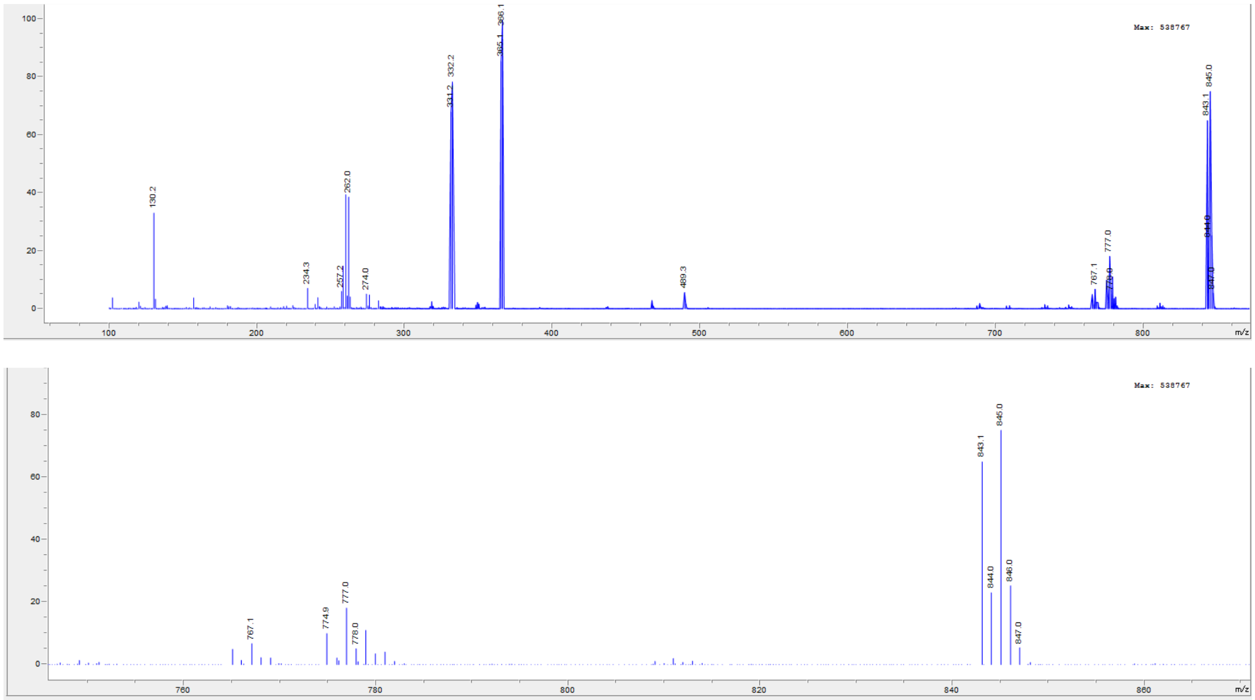

Figure S130 – LR–MS of [Eu(**Phencrypt**)]$^{3+}$ – [EuL · 2Br]$^{+}$ = calc. 777.0, found: 777.0.

References

(1) Crosby, G. A.; Demas, J. N. Measurement of Photoluminescence Quantum Yields. Review. *J. Phys. Chem.* **1971**, *75* (8), 991–1024. https://doi.org/10.1021/j100678a001.

(2) Brouwer, A. M. Standards for Photoluminescence Quantum Yield Measurements in Solution (IUPAC Technical Report). *Pure Appl. Chem.* **2011**, *83* (12), 2213–2228. https://doi.org/10.1351/PAC-REP-10-09-31.

(3) Beeby, A.; Clarkson, I. M.; Dickins, R. S.; Faulkner, S.; Parker, D.; Royle, L.; Sousa, A. S. de; Williams, J. A. G.; Woods, M. Non-Radiative Deactivation of the Excited States of Europium, Terbium and Ytterbium Complexes by Proximate Energy-Matched OH, NH and CH Oscillators: An Improved Luminescence Method for Establishing Solution Hydration States. *J. Chem. Soc. Perkin Trans. 2* **1999**, No. 3, 493–504. https://doi.org/10.1039/A808692C.

(4) Gratal, P. B.; Quero, J. G.; Pérez-Redondo, A.; Gándara, Z.; Gude, L. PhenQE8, a Novel Ligand of the Human Telomeric Quadruplex. *Int. J. Mol. Sci.* **2021**, *22* (2). https://doi.org/10.3390/ijms22020749.

(5) Lüning, U.; Müller, M. Concave Reagents. 2 Macrobicyclic 1,10-Phenanthrolines. *Liebigs Ann. Chem.* **1989**, *1989* (4), 367–374. https://doi.org/10.1002/jlac.198919890163.

(6) Rodriguz-Ubis, J.-C.; Alpha, B.; Plancherel, D.; Lehn, J.-M. Photoactive Cryptands. Synthesis of the Sodium Cryptates of Macrobicyclic Ligands Containing Bipyridine and Phenoanthroline Groups. *Helv. Chim. Acta* **1984**, *67* (8), 2264–2269. https://doi.org/10.1002/hlca.19840670833.

(7) *Dependence of the Photophysical Properties on the Number of 2,2'-Bipyridine Units in a Series of Luminescent Europium and Terbium Cryptates | Inorganic Chemistry*. https://pubs.acs.org/doi/10.1021/ic3010568 (accessed 2025-10-23).
(8) *Dependence of the Photophysical Properties on the Number of 2,2'-Bipyridine Units in a Series of Luminescent Europium and Terbium Cryptates | Inorganic Chemistry*. https://pubs.acs.org/doi/10.1021/ic3010568 (accessed 2026-02-18).
(9) Yi, J.; Bian, K.; Xu, Y.; Liu, D.; Wu, Z.; Liu, C.; Yang, W.; Zeng, W.; Cui, T.; Zhang, B. Small-Molecule Fe^{3+} -Based Magnetic Resonance Imaging Probe Enables Noninvasive Imaging of Pancreatic Zn^{2+} and β-Cell Function In Vivo. *J. Am. Chem. Soc.* **2025**, *147* (51), 47627–47637. https://doi.org/10.1021/jacs.5c17216.
(10) Firbank, M. J.; Coulthard, A.; Harrison, R. M.; Williams, E. D. A Comparison of Two Methods for Measuring the Signal to Noise Ratio on MR Images. *Phys. Med. Biol.* **1999**, *44* (12), N261–N264. https://doi.org/10.1088/0031-9155/44/12/403.
(11) Śmiłowicz, D.; Schlyer, D.; Boros, E.; Meimetis, L. Evaluation of a Radio-IMmunoStimulant (RIMS) in a Syngeneic Model of Murine Prostate Cancer and ImmunoPET Analysis of T-Cell Distribution. *Mol. Pharm.* **2022**, *19* (9), 3217–3227. https://doi.org/10.1021/acs.molpharmaceut.2c00361.
(12) Guzei, I. A.; Wendt, M. An Improved Method for the Computation of Ligand Steric Effects Based on Solid Angles. *Dalton Trans.* **2006**, No. 33, 3991–3999. https://doi.org/10.1039/B605102B.
(13) Casanova, D.; Cirera, J.; Llunell, M.; Alemany, P.; Avnir, D.; Alvarez, S. Minimal Distortion Pathways in Polyhedral Rearrangements. *J. Am. Chem. Soc.* **2004**, *126* (6), 1755–1763. https://doi.org/10.1021/ja036479n.
(14) Straub, L. C.; Adlung, M.; Wickleder, C.; Wickleder, M. S.; Rasche, B. Impact of 1,10-Phenanthroline-Induced Intermediate Valence on the Luminesence of Divalent Europium Halides. *Inorg. Chem.* **2023**, *62* (1), 497–507. https://doi.org/10.1021/acs.inorgchem.2c03647.
(15) Yu, G.; Liu, H.; Yan, W.; Guo, R.; Wu, A.; Zhao, Z.; Liu, Z.; Bian, Z. 4f → 3d Sensitization: A Luminescent EuII–MnII Heteronuclear Complex with a near-Unity Quantum Yield. *Mater. Horiz.* **2023**, *10* (2), 625–631. https://doi.org/10.1039/D2MH01123A.
(16) Bruker AXS LLC (2024). APEX6.
(17) Krause, L.; Herbst-Irmer, R.; Sheldrick, G. M.; Stalke, D. Comparison of Silver and Molybdenum Microfocus X-Ray Sources for Single-Crystal Structure Determination. *J. Appl. Crystallogr.* **2015**, *48* (1), 3–10. https://doi.org/10.1107/S1600576714022985.
(18) Sheldrick, G. M. XPREP, 2013b.
(19) Sheldrick, G. M. *SHELXT* – Integrated Space-Group and Crystal-Structure Determination. *Acta Crystallogr. Sect. Found. Adv.* **2015**, *71* (1), 3–8. https://doi.org/10.1107/S2053273314026370.
(20) Sheldrick, G. M. Crystal Structure Refinement with SHELXL. *Acta Crystallogr. Sect. C Struct. Chem.* **2015**, *71* (1), 3–8. https://doi.org/10.1107/S2053229614024218.
(21) Sheldrick, G. M. The SHELX homepage. http://shelx.uni-ac.gwdg.de/SHELX/.
(22) Dolomanov, O. V.; Bourhis, L. J.; Gildea, R. J.; Howard, J. a. K.; Puschmann, H. OLEX2: A Complete Structure Solution, Refinement and Analysis Program. *J. Appl. Crystallogr.* **2009**, *42* (2), 339–341. https://doi.org/10.1107/S0021889808042726.
(23) Guzei, I. A. Programs Gn. University of Wisconsin-Madison, Madison, Wisconsin, USA. **2007**.
